# The ‘Danse Macabre’—Neutrophils the Interactive Partner Affecting Oral Cancer Outcomes

**DOI:** 10.3389/fimmu.2022.894021

**Published:** 2022-06-16

**Authors:** Sara Hadjigol, Bansari A. Shah, Neil M. O’Brien-Simpson

**Affiliations:** ACTV Research Group, Division of Basic and Clinical Oral Sciences, Centre for Oral Health Research, Melbourne Dental School, Royal Dental Hospital, The University of Melbourne, Carlton, VIC, Australia

**Keywords:** neutrophil, immune cells, oral cancer, tumor microenvironment, myeloid cells, interaction, innate immunity

## Abstract

Over the past few decades, tremendous advances in the prevention, diagnosis, and treatment of cancer have taken place. However for head and neck cancers, including oral cancer, the overall survival rate is below 50% and they remain the seventh most common malignancy worldwide. These cancers are, commonly, aggressive, genetically complex, and difficult to treat and the delay, which often occurs between early recognition of symptoms and diagnosis, and the start of treatment of these cancers, is associated with poor prognosis. Cancer development and progression occurs in concert with alterations in the surrounding stroma, with the immune system being an essential element in this process. Despite neutrophils having major roles in the pathology of many diseases, they were thought to have little impact on cancer development and progression. Recent studies are now challenging this notion and placing neutrophils as central interactive players with other immune and tumor cells in affecting cancer pathology. This review focuses on how neutrophils and their sub-phenotypes, N1, N2, and myeloid-derived suppressor cells, both directly and indirectly affect the anti-tumor and pro-tumor immune responses. Emphasis is placed on what is currently known about the interaction of neutrophils with myeloid innate immune cells (such as dendritic cells and macrophages), innate lymphoid cells, natural killer cells, and fibroblasts to affect the tumor microenvironment and progression of oral cancer. A better understanding of this dialog will allow for improved therapeutics that concurrently target several components of the tumor microenvironment, increasing the possibility of constructive and positive outcomes for oral cancer patients. For this review, PubMed, Web of Science, and Google Scholar were searched for manuscripts using keywords and combinations thereof of “oral cancer, OSCC, neutrophils, TANs, MDSC, immune cells, head and neck cancer, and tumor microenvironment” with a focus on publications from 2018 to 2021.

## Introduction

Head and neck cancers (HNCs) are the seventh most common cancer worldwide and have a high mortality rate, with 177,384 deaths occurring in 2018, and a poor prognosis, with a 5-year relative survival rate of 68%. This survival rate is known to be poorer in developing countries (1–4). Oral cancer is often included in head and neck cancer statistics and represents 48% of HNC cases, with oral squamous cell carcinoma (OSCC) being the most common malignant lesion (approximately 90% of these cases) (5, 6). The OSCC develops in the oral cavity (namely, the lips, gums, lining of the cheeks and lips, front two-thirds of the tongue, floor of the mouth under the tongue, and roof of the mouth) and oropharynx (7). Despite advances in diagnosis and the availability of diverse treatment modalities, the global 5-year OSCC survival rate remains below 50% (8). Generally, the data support that with an earlier diagnosis comes a higher chance of survival with treatment. As patients with early-stage oral cancer have a 75% survival rate at 5 years, this decreases sharply to only a 35% survival rate for patients with advanced stages at diagnosis (9). This makes timely diagnosis and treatment essential for a good prognosis with OSCC. Though the oral cavity can be easily examined and assessed by direct visual inspection, most OSCC cases are diagnosed at an advanced stage (10). This most likely arises from the low rates of dental visits per year by people [e.g., on average, 56% of the Australian population sees a dentist once per year (11)], and most oral cancers commence as a painless surface lesion with erythema, minor elevation, and typically mimics benign processes in the mouth (12). Once lesions become intense masses, symptoms such as altered mucosa lining sensation, persistent sore throat, or ear infection, can appear, which then prompts medical intervention (9).

The etiology of OSCC is complex and is associated with several risk factors involving the interplay of the whole immune system, and recently, neutrophils have become the focus of several investigations as a pivotal cell in cancer development, which is the focus of this review. Identification of these factors has a significant impact on the prevention and early detection of cancer development. Although there are many risk factors associated with OSCC, alcohol and tobacco consumption, namely, smoking cigarettes, cigars, pipes, and chewing tobacco, are associated with 75% of OSCC tumors. Among the different compounds of cigarette smoke, nicotine is well known for its biological effects on the brain and other organs such as the oral cavity (13). Though nicotine is commonly acknowledged as non-carcinogenic, it is always accompanied in tobacco by carcinogens such as nitrosamines [i.e., 4-(methylnitrosamino)-1-(3-pyridyl)-1-butanone (NNK) and N’-nitrosonornicotine (NNN)] (14). It has been shown that binding of nitrosamines to the nicotinic acetylcholine receptor promotes cell proliferation and creates a microenvironment for tumor growth (15). Overall, tobacco users have a five-fold increased risk of developing oral cancer and a 10-fold increase in developing laryngeal cancer in comparison to non-users (16). Significantly, smoking increases the neutrophil to lymphocyte ratio (NLR) a known prognostic marker, in cancer patients, leading to poor prognosis (17). Alcohol is known to decompose the lipid composition of the epithelial cell membrane of the oral mucosa, thus facilitating carcinogen penetration (18). Frequent use of alcohol alone may result in OSCC *via* three mechanisms: (i) DNA adduct formation, (ii) interference with the DNA-repair mechanism, and (iii) generation of ethanol-related reactive oxygen metabolites (19). Alcohol use is known to affect the NLR of HNC patients, leading to poor prognosis (20). The consumption of alcohol alongside tobacco is known to have a multiplicative impact on increasing the risk of oral cancer, especially when both products are used on a regular basis (21).

Chronic viral infections of human cells can induce mutagenesis, potentially commencing cellular transformation and giving rise to malignant disease (22). Human papillomavirus (HPV) infection has recently been associated with the carcinogenesis of OSCC. In particular, HPV-16 is frequently isolated from oropharynx cancers of the tonsils and base of the tongue (23). It is estimated that 15–20% of all OSCC are related to high-risk HPV infection, which is the most common sexually transmitted virus (22). Likewise, HPV-DNA can be found in up to 70% of oropharyngeal squamous cell carcinomas (OPSCCs), particularly localized to the tonsils (24). As a result of poor oral hygiene, gingival inflammation may facilitate HPV penetration through the oral epithelial superficial layers to invade the basal layer (25). The association of HPV status and neutrophil infiltration in OSCC or OPSCC tissues has yet to be fully elucidated. However, one study by Li et al. (26) found that HPV positive OSCC patients had low levels of neutrophils, in part due to HPV positive OSCC cells expression resulting in low levels of the neutrophil chemotactic factor IL-8. Although studies have found that neutrophil levels are lower in HPV positive OSCC/HNC patients, other studies of patients have found the opposite or no significant association, indicating the complexity of this association and the requirement of further investigations to understand this relationship (27–30). In addition to HPV, Epstein–Barr virus (EBV), an oncogenic double-stranded DNA virus, is known to be involved in neoplastic transformation in oral cancers such as nasopharyngeal carcinoma (31). Nearly 60% of OSCCs were EBV genome positive (32) and increased expression of EBV correlates with poor OSCC prognosis (33, 34). Compounding this is that a high EBV DNA titer has been found to correlate with a high NLR and reduce overall survival (35). Most epidemiological studies show that HNC, and specifically oral cancer, typically occurs in the fifty to seventy-year-old age group (36). Nevertheless, there are reports that show 5% of HNC patients are in younger age groups (37). This correlates with higher rates of smoking and use of other drugs in younger age groups (38), and more recently, the increased prevalence of HPV (37).

More than 700 bacterial species are reported to be part of the bacterial flora in the oral cavity. In a healthy oral cavity, bacteria interact with each other and maintain a “good” balance. However, through poor oral hygiene, diet, or infection, this balance is broken, causing dysbiosis, which favors the growth of certain oral pathogens, leading to diseases such as caries and periodontal disease (39). Recent studies have confirmed a close link between OSCC and oral bacteria, which may present a fresh view and new potential targets for diagnosis and treatment of OSCC (40, 41). A study using a 16S rRNA V3-V5 marker gene approach to compare oral bacterial DNA isolated from oropharyngeal and oral cavity squamous cell carcinoma patients and healthy subjects demonstrated the comprehensive relationships between OSCC and specific oral bacteria (42). Also, several studies have shown that oral bacteria such as *Porphyromonas gingivalis* and *Fusobacterium nucleatum* influence the development and progression of OSCC by altering the microbiota, which contributes to cancer development by enhancing cell proliferation, inhibiting apoptosis, and improving tumor invasion and metastasis (43, 44). It has been observed that *P. gingivalis* induces an increase in the oral tumor cell proliferation rate by modifying the expression levels of oncogenic-relevant α-defensin genes (45). Furthermore, *P. gingivalis* infection in OSCC patients has been positively correlated with increased levels of tumor-associated neutrophils and poor prognosis (46).

Many other factors are associated with OSCC, such as gender, previous cancer, prolonged sun exposure, poor oral hygiene, poor diet, family history, and various genetic mutations. Generally, OSCC is 2–5-fold more common in men (47). People who have previously had oral cancer have a greater risk of developing further oral cancer, particularly if alcohol and/or tobacco use is continued. OSCC develops from pre-existing, possibly malignant disorders like oral erythroplakia, lichenoid dysplastic lesions, and leukoplakia (48). Furthermore, combinations of specific genetic mutations and polymorphisms have been associated with an increased risk and development of oral cancers (49, 50). The multitude of risk factors and mechanisms that may lead to OSCC demonstrate the complex nature of the disease and the interplay of neutrophils with these factors and other immune cells and mechanisms in the initiation and development of OSCC is an area of research that requires investigation.

In addition to all the above-mentioned predisposing conditions, there are metabolic factors that can increase the risk of oral cancer. High concentrations of reactive oxygen species (ROS) lead to oxidative stress, which plays a crucial role in the destruction of key cellular components, such as DNA, proteins, and cell membranes. Because of these destructive mechanisms, ROS may contribute to the initiation and progression of multistage carcinogenesis (51). Oxidative stress is a key factor in the pathogenesis of cancer and can arise from poor nutritional habits, mainly a diet low in vegetables and fruits, which are rich sources of antioxidants, and lifestyle choices and practices (52, 53). In one study, patients with HNC had high oxidative stress and reduced antioxidant defense (54). Furthermore, OSCC patients had significantly higher levels of ROS (55). Given that neutrophils are major producers of ROS and neutrophil infiltrate increases in OSCC tissues lead to poor prognosis, this may be a novel therapeutic target.

Following the initiation of oral cancer, its development and progression at specific sites is heavily influenced by the immune system (56). It is now understood that specific cells of the immune system can have anti- and pro-tumor effects (57). With the development of immunotherapies to complement the standard care treatments of surgery, chemotherapy, and radiotherapy, it has become increasingly important to know how specific immune cells influence tumor growth, progression, and metastases in OSCC. It is evident that many of the risk factors for OSCC may be associated with the presence/infiltration of neutrophils in the tumor, but how this cell population interacts in the tumor environment and with other immune cells has received little attention. This review will focus on describing the interplay of neutrophils and major subpopulations of cancer-associated immune cells and other factors, focusing from tumor initiation to metastatic colonization in OSCC.

## Tumor Microenvironment and Immune Evasion

Several studies have supported the synergistic role of the tumor microenvironment (TME) in oral cancer development (58). The TME in HNSCC comprises many different cell populations, such as tumor cells, tumor stromal cells, namely, stromal fibroblasts, endothelial, and various infiltrating immune cells (neutrophils, macrophages, regulatory T cells, myeloid-derived suppressor cells, natural killer cells, platelets, and mast cells), and heightened non-cell components of the extracellular matrix (ECM) such as collagen, fibronectin, hyaluronan, laminin, among others (57, 59–61). Within the TME, malignant cells interact with the surrounding and infiltrating cells synergistically to promote cancer progression and that both the innate and adaptive immune responses contribute to tumorigenesis (62, 63). In the initial stages of tumor development, cytotoxic immune cells such as natural killer (NK) and CD8+ cytotoxic T cell lymphocytes (CTLs) identify and kill the more immunogenic cancer cells (64). Cancer cells that are less immunogenic and go undetected by the immune system are therefore positively selected and the cancer grows. As the neoplastic tissue progresses to a clinically evident tumor, different subsets of inflammatory cells impact the fate of the tumor (57). Although N1 neutrophils, M1 macrophages, dendritic cells (DCs), T helper 1 (Th1), and CTLs are involved in anti-tumor immunity (65, 66), certain immune cells such as N2 neutrophils, myeloid-derived suppressor cells (MDSC), M2 macrophages, tolerogenic DCs, T helper 2 (Th2), and T regulatory cells (Tregs) play an essential role in aiding and supporting cancer cell growth (65).

**Figure 1:**
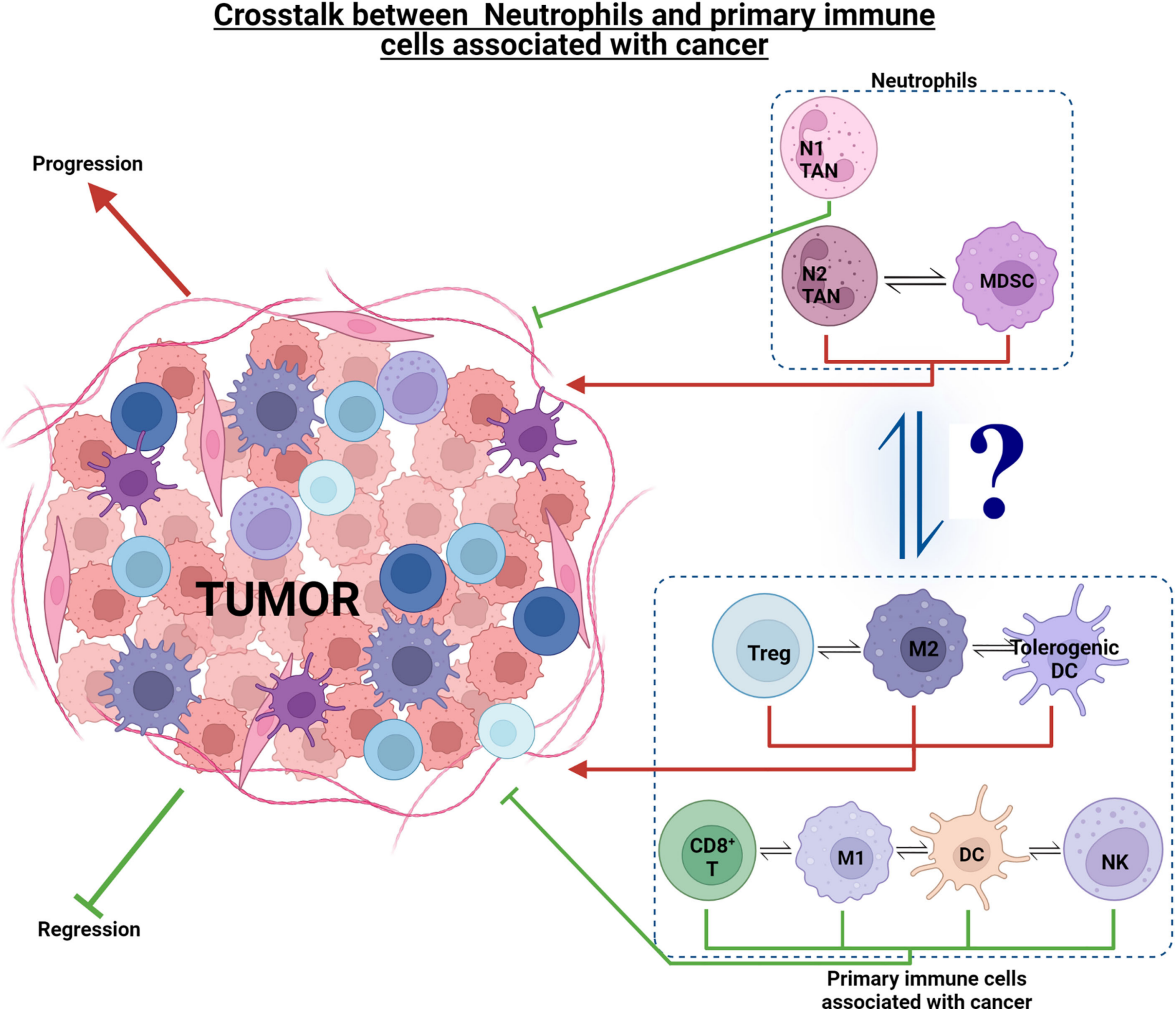
Effect of neutrophils and other immune cell sub-phenotypes on tumorigenesis. Immune cells such as CD8^+^ T, M1 TAMs, DCs, NK, and N1 TANs exhibit an anti-tumor response and aid in tumor regression. On the other hand, tumorigenic cells such as Treg, M2 TAMs, tolerogenic DCs, N2 TANs, and MDSCs, exhibit pro-tumor response and aid in tumor progression. There is complex interplay within the anti- and pro-tumor cell groups, as well as interaction of neutrophils with these cell groups to drive tumorigenesis in the TME ecosystem.

Understanding the mechanisms of how cancer cells avoid the immune system is a significant and on-going challenge in oncology. There are several well described mechanisms, albeit with a T cell and DC focus, by which tumors avoid the immune system and limit effective anti-tumor immunity by the host, these being: (i) induction of Treg cells (CD4^+^ CD25^+^ CTLA4^+^ GITR^+^ FOXP3^+^) that can suppress tumor-specific CD4/CD8 T cells (67); (ii) production of immunosuppressive cytokines, e.g., interleukin (IL)-10 (IL-10) and transforming growth factor beta (TGF-β) (68); (iii) decreased MHC-I expression due to gene loss *via* structural changes or β2-microglobulin synthesis alteration; (iv) induction of dendritic cell (DC) anergy; (v) inhibiting DC maturation *via* producing and releasing granulocyte–macrophage colony-stimulating factor (GM-CSF), IL-6 and IL-10 by tumor cells; and (vi) defective MHC-I antigen presentation *via* attenuation of the costimulatory molecule B7-1 (CD80) (69). It has been shown that CD8^+^ T cell tolerance can be induced by Gr-1^+^ immature myeloid cells (ImC) isolated from tumor-bearing mice (70). IL-6 has a suppressive action on DC maturation, which was attributed to the activation of the transcription factor STAT3 (71). IL-10 is thought to reduce co-stimulatory molecule expression on immature DC, resulting in tolerogenic APCs (72). Although these studies have a lymphocyte focus, these key cytokines (IL-6, IL-10, TGF-β, and GM-CSF) stimulate myeloid cells and activate the STAT3 signaling pathway in neutrophils and other myeloid cells such as macrophages (68, 71–74). All of these aid tumor growth and immune evasion, thus highlighting the complexity in cancer and the need to view the impact of one cell or chemokine/cytokine on the whole immune response and recognize that one cell ‘class,’ e.g., neutrophils, will have many faces/phenotypes in cancer (**Figure 1**) (73).

The type, proportion, and activation state of tumor infiltrating lymphocytes and myeloid cells are becoming increasingly important as prognostic markers for many cancers. A favorable prognosis is associated with a number of differing immune factors such as: high levels of memory CD8^+^ T cells, high expression of Th1 cytokines, i.e., interferon gamma (IFN-γ) and IL-1, the development of a tertiary lymphoid structure (TLS) associated with the tumor, increased levels of cytotoxic mediators (granzymes, granulysin), low neutrophil–lymphocyte Ratio (NLR) and low to moderate vascularization of the tumor (75). Poor prognosis is associated with the lack of TLS, infiltration of neutrophils (particularly N2), M2 macrophages, and extensive vascularization (76). In many solid cancers, high levels of tumor-infiltrated T cells are associated with a good prognosis (77); in contrast, the influx of neutrophils and tumor-associated neutrophils (TANs), and high levels of macrophage infiltration, particularly the phenotype of tumor-associated macrophages (TAMs), are linked with a poor prognosis and a reduction in overall survival (78).

## Neutrophils: Cells With a Multitude of Roles

In the context of cancer, neutrophils have had less attention by comparison to other immune cells because it was thought that the lifespan of neutrophils is too short (7–10-hour circulating half-life in humans) to impact cancer development and progression (79). However, cytokines released by tumor cells, such as G-CSF, IL-1β, IL-6, or tumor necrosis factor (TNF), have been proposed to prolong neutrophil lifespan, indicating that neutrophils may have a significant impact on cancer (80, 81). Recently, it has been shown that uncoupled biological and chronological aging of neutrophils contributes to the progression of cancer and promotes advanced stages of malignant disease (82). Using a mouse squamous cell carcinoma cell line (SCC VII), we showed a noteworthy positive association between the RNA expression levels of formyl peptide receptor 1 (FPR1), an established marker gene of neutrophils, and of C-X-C motif chemokine receptor 4 (CXCR4), whose gene product increases during neutrophil aging on the surface of these immune cells, with higher tumor stages (83, 84). Activation of the NLRP3 inflammasome in perivascular macrophages by tumor-released Damage-associated molecular patterns (DAMPs) such as S100A8/9 induce the synthesis of inflammatory mediators that upregulate adhesion and signaling molecules on the surface of microvascular endothelial cells, in turn promoting the trafficking of aged neutrophils to the perivascular space (84). Following antibody-mediated depletion of aged neutrophils, a significant decrease in the growth of tumors was observed in experimental HNSCC (84). Aged neutrophils are related to a more pro-tumorigenic state. They support cancer cell proliferation *via* the release of neutrophil elastase (84, 85). The neutrophil-to-lymphocyte ratio (NLR) has recently been introduced as a better prognostic factor for survival in several types of solid tumors, including OSCC (86–89), as opposed to neutrophil levels alone (90), although the mechanisms involved in high NLRs (typically >3) are yet to be determined (91, 92). Several studies have found that higher NLR showed higher mortality rates compared with those with lower NLR and was associated with more advanced or aggressive cancer (93–95). In general, neutrophilia appears to be linked with a poor prognosis in cancer. However, the inverse might also be true to some extent in the context of antibody therapy, which has emerged as an important weapon in the anticancer armament (96–99). Recombinant technology presents huge opportunities to design antibodies to meet clinical requirements, including the reduction of immunogenicity (100). For example, antibodies can prevent tumor growth factors or their receptors, trigger immunologic attack on the tumor, or be used to provide payloads, for example, radioisotopes, cytotoxic drugs or toxins, and nanoparticles (101, 102).

It is known that the formation of neutrophil extracellular traps (NETs) activates platelets and stimulates thrombosis (103), and interestingly, an increased risk of cancer-associated venous thromboembolism (VTE) has been reported in numerous types of cancer, including OSCC (104). Previous studies have shown that NETs capture and operate as adhesion substrates for cancer cells, and using this process promotes metastatic dissemination (105). Park et al. (106) have shown that targeting NETs with DNase I-coated nanoparticles efficiently reduces metastasis in an *in vivo* cancer model, confirming that neutrophils and NET production are an important mechanism in cancer progression (106). A recent study investigating the myeloperoxidase (MPO) and histone expression using immunohistochemistry showed NETs in the tumor tissue of patients with OSCC (107). It has been shown, in OSCC patients, that the interaction between cancer cells and neutrophils increases NET formation *via* the PI3K/Akt/PBK pathway (108). This co-existence of NETs and cancer demonstrates that the presence of NETs may be a marker of poor prognosis, highlighting their potential as a target for cancer therapy (109).

A novel prognostic model of HNSCC patients based on six-NET-related genes (Annexin A3 (ANXA3), lactotransferrin (LTF), colony-stimulating factor 2 (CSF2), glyceraldehyde-3-phosphate dehydrogenase (GAPDH), selectin P ligand (SELPLG), and cytochrome b-245 beta chain (CYBB)) was constructed that might be beneficial for developing personalized treatment directed at neutrophils (110). Irregular ANXA3 expression is associated with the development, occurrence, metastasis, and drug resistance of cancers (111). The LTF inhibits the development and release of NETs, which might be associated with the anticancer role of the gene (112). These studies indicate the potential clinical approaches for targeting neutrophils as a therapy in cancer treatment.

Polymorphonuclear granulocytes (PMN) from the peripheral blood of patients with late stage HNC showed a significantly lower inducible production of reactive oxygen species (ROS) and reduced spontaneous apoptosis compared with PMN from healthy donors (113). However, another clinical study showed there was an acceleration in the apoptosis of circulating PMNs of oral cavity cancer patients due to higher caspase-8 activity and elevated activity of the TRAIL-mediated mitochondrial cascade (114). Though this data may seem conflicting, it does indicate that peripheral blood PMN from HNSCC patients and healthy donors show distinct functional differences.

Several studies have shown that neutrophils have various and conflicting roles in cancer. After transmigration into tumor tissue, neutrophils [referred to as tumor-associated neutrophils (TANs)] go through dramatic changes in their activity and phenotype, depending on the cytokines and growth factors they encounter in the TME. Because of the minor size of primary tumors in HNC, data pertaining to TANs are limited (115). In a European gastric cancer cohort study, immunohistochemical staining of myeloperoxidase was used to show a correlation between TAN density and survival in women but not in men. These findings indicate a possible sex-specific prognostic effect of TANs (116, 117). In two independent clinical cohorts, the ratio of CD8^+^ T cells to TANs within the tumor was associated with anti-PD1 monotherapy failure in non-small cell lung cancer (NSCLC) patients, indicating that neutrophil antagonism may be a sustainable secondary therapeutic approach to boost ICI treatment outcomes (118). Recently, an association between the resistance of mismatch repair-deficient tumors to anti-PD-1 monotherapy and abnormal neutrophil accumulation within the tumor was reported (119).

TANs express CD11b^+^ Ly6C^int^ Ly6G^hi^ in mice and CD11b^+^ CD14^−^ CD66b^+^ CD15^hi^ in humans (120). Like the M1/M2 macrophage phenotype, TANs are suggested in cancer to exist in two polarization states, these being the anti-tumor (N1) or pro-tumor (N2) phenotypes (121). Regardless of the growing interest in TANs in recent years, our current understanding of the role of neutrophils in tumor development is primarily based on murine models of cancer. In a human cancer population, low-density neutrophils (LDN) (N2 like) and high-density neutrophils (HDN) (N1 like) both express CD11b, CD66b, and CD15, but LDN express these at a higher level (122). Several studies in OSCC have shown neutrophils to express high levels of one or more of these markers in human cancer patients, consequently leading to poor prognosis (26, 123, 124). Furthermore, it has been shown that it is feasible to polarize blood-derived primary human neutrophils toward N1- and N2-like phenotypes *in vitro* (125). Also, human neutrophils incubated under a tumor-mimicking *in vitro* environment were found to highly express the typical N2 receptor CXCR2 on their surface and secreted elevated amounts of IL-8 (125). Thus, although human neutrophils do not have definitive N1 and N2 markers as in mice, there is, potentially, a N1 and N2 like neutrophil, i.e., HDN and LDN, respectively, which would appear to play the same role in humans. However, there is significant debate around human N1 and N2 neutrophils as a study by Brandau et al. (126) found that in peripheral blood human HNC, lung, bladder, and ureter cancer patients had a CD66+ PMN population but based on their LDN and HDN profile, the CD66+ LDN cells expressed low levels of CD11b and CXCR2. Further studies will be needed to investigate whether humans have N1 and N2 populations, as in mice the N1 and N2 phenotypes have a profound effect on cancer development, and from the few studies in humans that have described a N1-like or N2-like neutrophil phenotype they appear to also have a profound effect on tumor immunity.

At the early stages of tumor development, neutrophils mostly remain located at the edges of the tumor and have an N1 phenotype, eliminating cancerous cells and limiting metastatic seeding. As the tumor progresses, neutrophils are often found deeper within the tumor and transition toward a more aggressive N2 phenotype, enabling tumor growth to be supported (127). A humanized mouse model of hepatocellular carcinoma (HCC) showed that CCL2^+^ and CCL17^+^ chemokines, which are part of the N2 signature, promote macrophage (F4/80+) and regulatory T (Treg) cell (FoxP3+) infiltration into the TME by activating the MAPK and PI3K signaling pathways, which stimulate neovascularization, enhance growth and metastasis, and contribute to sorafenib (a kinase inhibitor drug) resistance (128). This switch of N1 to N2 with the maturation of cancer is very reminiscent of the M1 and M2 switches in TAMS, strongly suggesting that there is synergy between the immune cells and their respective functions and roles in cancer/tumor development. In the earliest stages of cancer, TANs stimulate T-cell proliferation and IFN-γ release (129), while in established tumors, TANs are immunosuppressive and linked with a more pro-tumor phenotype with tumor progression (130). In OSCC, *P. gingivalis* infection contributes to the enhanced CXCL8 and CCL2 secretion in the TME, which in turn recruits CD66b^+^ TANs to the site of neoplastic cells and the promotion of tumor development (131, 132). This strong immune induction of CXCL2, CXCL8 from neutrophils by *P. gingivalis* is well known in oral disease research and may aid in our understanding of how this bacterium is associated with a poor prognosis in OSCC patients (131, 132).

It has been shown that N2 neutrophils contribute to tumor growth by several mechanisms such as increased expression of pro-angiogenic genes (MMP9, VEGF) with absent IFN-β and is acquired by neutrophils following the TGF-β treatment/exposure (121, 133–135). MMP-9 is a protease produced predominantly by neutrophils (N2 neutrophils in mice) and located in its tertiary granules (136) and is involved in elevated cancer cell proliferation, angiogenesis induction, tumor growth, inhibition of cancer cell apoptosis, promotion of neutrophil extravasation, and migration into tissues by the degradation of the extra-cellular matrix (137, 138). An *in vitro* study using two oral squamous cell carcinoma cell lines (UT-SCC-43A and UT-SCC-43B) showed that the expression of MMP-2 and MMP-9 was downregulated in both cell lines after being incubated with human neutrophil peptide (HNP)-1 (a N1 neutrophil produced peptide), indicating a protective role of HNP-1 against the spread of metastatic cells (139). The antitumor mechanisms of another peptide, melatonin (Mel), are linked with anti-proliferation, apoptosis promotion, migration, invasion inhibition, and anti-angiogenesis (140). TANs were suppressed by Mel in a MMP-9-dependent manner in OSCC (141). These studies indicate that targeting MMP-9 expression is a possible therapeutic avenue to explore.

The serine protease neutrophil elastase (NE), located in neutrophil azurophilic granules (142), promotes the detachment of tumor cells through the degradation of the adhesion molecule E-cadherin, decreasing the stability of the tumor and increasing metastasis. Significant elastase expression has been shown to be upregulated in OSCC (143, 144). The elastase and serine protease inhibitor Secretory Leukocyte Protease Inhibitor (SLPI) was considerably reduced in OSCC compared with normal oral epithelium, and cancer cells treated *in vitro* with SLPI had reduced invasive ability, suggesting that SLPI is a therapeutic lead as it may decrease many tumor-promoting events (145). Another, neutrophil targeting therapy may be TGF-β blockade or IFN-β treatment as both promote neutrophil reversion to a cytotoxic N1 subset while expressing high levels of intercellular adhesion molecule 1 (ICAM1) and TNF-α and increasing NET formation (146, 147). Taken together, new novel cancer therapies may involve modulation of neutrophil function through alterations of the tumor microenvironment by blocking TGF-β activity or enhancing IFN-β activity instead of depleting specific neutrophil subsets such mature and immature low-density neutrophils (LDN) that accumulate continuously with cancer progression (148).

It has been shown that an increased neutrophil-to-lymphocyte ratio (NLR) is linked with poor survival in patients undertaking chemoradiotherapy or radiation for nasopharyngeal carcinoma (149). Another study revealed that in patients with nasopharyngeal carcinoma, NLR was a significant predictor of both survival and response to chemoradiotherapy (150). Several retrospective cohort studies have evaluated the prognostic significance of NLR in patients with oral squamous cell carcinoma (95, 151–153). They found that a low NLR was the only independently favorable marker of both overall survival and distant control in patients with OSCC; in contrast, a high NLR was associated with worse overall survival. These studies suggest that preoperative NLR in the peripheral blood is an important prognostic factor for OSCC and is valuable in predicting OSCC development.

## The Anti-Metastatic Role of Neutrophils

Although numerous cancer studies support the pro-tumorigenic role of neutrophils, there is evidence that they also remove cancerous cells and limit metastatic seeding. Cytotoxic action of neutrophils towards cancer cells is mostly evident in early stages of tumor development in the form of N1 TANs, and killing has been shown to require a high level of target specificity (79). To induce tumor cell apoptosis, activated neutrophils are required to identify cancer cells as targets through Receptor for Advanced Glycation End products (RAGE)-Cathepsin G (directly) (154) or in an antibody dependent fashion (ADCC) (155). High expression of RAGE is observed in OSCC patients and is associated with depth of invasion (156, 157). It has been shown that RAGE expression is responsible for migration, invasion, and MMP-9 production in patients with OSCC, thus representing a possible therapeutic candidate in treating OSCC patients by enhancing N1 activity (158). After cancer cell identification, neutrophils then need to have physical contact with the tumor cells in order to release cytotoxic mediators such as myeloperoxidase (MPO), H_2_O_2_, reactive oxygen species (ROS), and proteases (159). Neutrophil cytotoxicity is Ca2^+^ dependent and is mediated by the transient receptor potential cation channel, subfamily M, member 2 (TRPM2), a ubiquitously expressed H_2_O_2_-dependent Ca2^+^ channel (160). TRPM2 expression is increased in cancerous tissues, making tumor cells more susceptible to neutrophil cytotoxicity (161). Using a breast cancer model, it has been shown that reduced expression of TRPM2 in tumor cells allowed neutrophil immune evasion but also led to tumor growth retardation, albeit accompanied by an increase in metastatic potential (162). Inhibiting the overexpression of TRPM2 in human tongue squamous samples with the small interfering RNA technique (shRNA_TRPM2_) resulted in enhanced apoptosis of SCC cells and reduced the migratory abilities of SCC cells (163). Studies have shown that TRPM2 expression is elevated in circulating tumor cells (CTC) compared with the primary tumor, rendering CTC more susceptible to neutrophil cytotoxicity (162). Neutrophils as well as secreting H_2_O_2_ are able to suppress metastasis *via* their expression of thrombospondin 1 (TSP1) (164) and MET proto-oncogene, encoding the tyrosine kinase receptor for Hepatocyte Growth Factor (HGF), which regulates invasive growth (165, 166). TSP1 can be induced in neutrophils by a peptide derived from prosaposin, a precursor of sphingolipid activator proteins (164). It is reported that MET, induced by tumor inflammatory stimuli such as TNF-α, is essential for neutrophil chemoattraction and cytotoxicity in response to its ligand hepatocyte growth factor (HGF). C-MET-HGF stimulation leads to neutrophil transmigration across an activated endothelium and the production of inducible nitric oxide synthase (iNOS). Subsequently, MET/HGF-dependent nitric oxide release by neutrophils assists in cancer cell killing, which greatly dampens tumor growth and metastasis (165). It has been shown that hypoxia activates HGF/c-Met signaling in a hypoxia-inducible factor-1 (HIF-1) dependent manner, leading to the invasive growth of cancer cells through activating the PI3K/Akt pathway (167). These studies indicate that targeting the HIF-1α/c-Met signaling pathway using synthetic small-interfering RNA could be a useful new approach to the treatment of OSCC patients.

## Myeloid-Derived Suppressor Cells (MDSCs)—Pathologically Activated Neutrophils?

**Figure 2:**
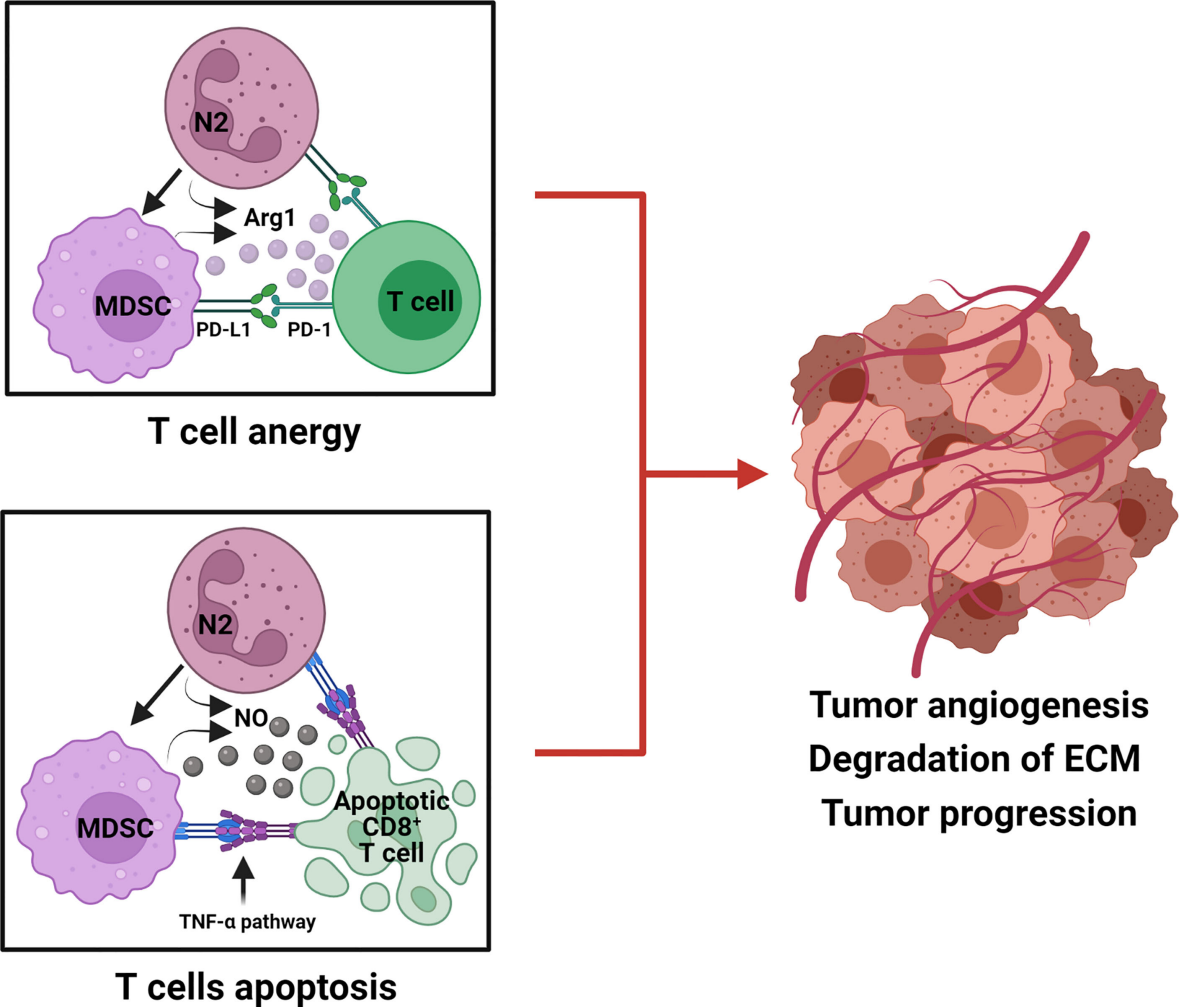
Tumorigenic role of TANs (N2) and MDSCs in the suppression of T-cell responses. N2 TANs differentiate into MDSC, an activated and more immunosuppressive neutrophil phenotype. Both N2 and MDSC produce Arg 1 and upregulate PD-L1 to cause T-cell anergy by modulating PD-L1/PD-1 signaling. N2 and MDSC also produce nitric oxide (NO) which initiates the TNF-α pathway to induce CD8^+^ T-cell death, *via* apoptosis. N2 and MDSC hinder anti-tumor T-cell function by anergy and apoptosis and promote tumor progression.

Myeloid-derived suppressor cells (MDSCs) have been described in humans and mice and occur as two main sub-groups: monocytic MDSCs (Mo-MDSCs), granulocytic or polymorphonuclear MDSCs (G-MDSCs/PMN-MDSCs), and a third sub-type termed early MDSCs (eMDSCs) and their discovery has been recently reviewed (168). Of the two main sub-groups, polymorphonuclear myeloid-derived suppressor cells (PMN-MDSCs) and neutrophils share the same origin cell type, the differentiation pathway, and are phenotypically and morphologically alike, with both being identified in oral cancer patients (169–171). A recognized distinguishing feature of PMN-MDSCs is that they have been reported to be more immunosuppressive than immature neutrophils (172). Currently, several studies have attempted to distinguish PMN-MDSCs from activated immature or mature neutrophils as the heterogeneity of PMN-MDSCs means that they are indistinguishable from activated neutrophils as they share the same phenotypic markers: CD14^−^ CD15^+^ CD66b^+^ CD16^+^ and CD11b^+^ CD33^+^ HLA-DR^−^ (168, 173–176). Recently, for human PMN-MDSCs, the lectin-type oxidized LDL receptor 1 (LOX1) has been suggested as a distinguishing marker (177). In mice, the proportion of MDSCs has been shown to increase significantly within the tumor microenvironment (178) and represents potent suppressors of antitumor immunity (179). To regulate an immunosuppressive response, TANs and MDSCs block T-cell proliferation by releasing ARG1 and modulate PD-L1/PD-1 signaling, a potential tumor escape mechanism (**Figure 2**) (180, 181).

In a murine cancer model granulocytic myeloid-derived suppressor cells (G-MDSCs also known as PMN-MDSCs) and TANs induced CD8 T-cell apoptosis *via* the TNF-α pathway and NO production, thereby promoting a tumor-supportive environment (182). Further, using the established 4-nitroquinoline 1-oxide (4NQO)-induced oral cancer mouse model, Chu et al. (170) showed that there was a significant progressive increase in the proportion of MDSCs in the spleens and peripheral blood of 4NQO-treated mice compared to control mice, suggesting that MDSCs contribute to oral cancer progression (170). MDSCs were initially defined in HNSCC patients as immature CD34^+^ cells presenting the ability to suppress the activity of T cells (183, 184). Another study showed that CD34^+^ cells in HNSCC patients can be differentiated into cells that phenotypically and functionally resemble dendritic cells (185). Though MDSCs (pathogenically activated neutrophils) have been originally recognized for their immune-suppressive function in cancer, lately their presence has been associated with other activities within the TME, including promotion of tumor angiogenesis, degradation of extracellular matrix, and the formation of premetastatic niches (186, 187). The role of PMN-MDSCs in cancer and immunity, how they interact with other immune cells to affect their actions, is currently being defined and thus are probably a major foci of novel therapeutics, treatments and prognostic and diagnostic factors (168).

## Macrophages and Neutrophils: An Immunological Partnership Aiding Cancer Growth

The crosstalk between tumor cells and infiltrated neutrophils and macrophages can promote and drive tumor growth and metastasis (188). Arising from a common progenitor lineage, the multi-layered roles of TANs and TAMs are implicated in almost every step of tumor growth and metastasis. Both TAMs and TANs use multiple overlapping pathways to crosstalk with T cells, including engagement of immune checkpoints and secretion of cytokines, resulting in tumor immune escape, as well as angiogenesis and invasion (189, 190). It is known that activated neutrophils provide signals for the activation, maturation, and recruitment of monocytes/macrophages, NK cells, and DCs by releasing IL-8, TNF-α, macrophage inflammatory protein-1α (MIP-1α), and MIP-1β, indicating that neutrophils play a central role in the involvement of these three major cell phenotypes in immunity (191–194). Murine neutrophils secrete myeloperoxidase (MPO) and have a direct tissue damaging effect. They are also recognized by tissue resident macrophages expressing macrophage mannose receptors (MMRs) (195, 196). This recognition of MPO by MMR^+^ macrophages activates macrophages, which in return overproduce neutrophil survival factors, namely, IL-1, IL-6, IL-8, TNF-α, and GM-CSF, which activate neutrophils and upregulate their survival mechanisms (191, 197). Though limited direct evidence that supports TAN and TAM interaction through MPO and the MMR is available, high density MPO-positive neutrophil infiltration has been reported in colorectal cancer (198). Intriguingly, this neutrophil and macrophage interplay results in an increase in neutrophil half-life/survival, which is a feature of N2 neutrophils, TANs, and PMN-MDSCs. However this dynamic requires further investigation. The importance of murine neutrophil MPO has been shown using a novel tripeptide MPO inhibitor [N-acetyl lysyltyrosylcysteine amide (KYC)], which diminished lung tumor burden, suggesting that targeting neutrophil MPO is a novel cancer treatment (199). A cytokine impacting the activation of macrophages and neutrophils is TGF-β, which as well as being produced by many infiltrating cells in the TME, is also highly expressed by cancer cells, including OSCC cells (200). It is thought that the interplay of TGF-β with macrophages and neutrophils generates M2-like and N2-like cells suggests a close relationship between TAMs and TANs in the same TME and the possibility that recruitment of macrophages by neutrophils may lead their N2-like polarization (201). However, studies must confirm whether the crosstalk between TANs and TAMs in the TME is comparable to the known interactions between neutrophils and macrophages in a non-tumoral chronic inflammatory environment.

Despite the significant role of macrophages in promoting host defenses, their inappropriate or extended activation can lead to immune dysregulation and the promotion of cancer. The role of macrophages in tumor progression and interaction with other infiltrating immune cells is yet to be completely clarified, partly because of the plasticity of macrophages and the conflicting roles of their different phenotypes. In response to malignant cell-derived growth factors and chemokines including colony-stimulating factor-1 (CSF-1) (202), VEGF-A (203), chemokine (C–C motif) ligand such as CCL2 (MCP1) (204), CCL18, CCL20 (MIP3a), and CXCL12 (SDF1), bone-marrow derived monocytes or tissue-resident macrophages are recruited into the tumor site and are then termed Tumor Associated Macrophages (TAMs) (205). CSF-1 in binding to its receptor on monocytes and macrophages ((macrophage colony-stimulating factor receptor (M-CSFR)) is known to have a critical role in differentiating the phenotypes of macrophage subsets in tumors (206, 207). Significantly, murine neutrophils and MDSCs have been shown to be major producers of CSF-1, resulting in macrophage polarization and an immune tolerant/suppression phenotype, further strengthening, albeit yet to be proven in cancer, the interplay of neutrophils and macrophages in the TME (177, 208).

During carcinogenesis, TAMs mainly exhibit an M1-like polarization that results in the elimination of the more immunogenic cancer cells. As the tumor progresses, the changing composition of the TME provokes an M2-like re-polarization of TAMs that is pro-tumorigenic and supports primary tumor growth and metastatic spread (209, 210). The effect of TAMs on tumor progression can depend on the tumor nature, the type of TME, and the intra-tumoral location of TAMs (211). It has been suggested that TAMs can combine the properties of M1 and M2 macrophages (212). Hence, the presence of TAMs by themselves does not have prognostic value, and so an M1/M2 ratio is used. Low and high M1/M2 TAM ratios are associated with poor and good prognosis, respectively (213). The differentiation to M1 or M2 phenotype and the ratio of M1/M2 is heavily aided and/or influenced by the presence and secretion of cytokine/chemokines by neutrophils and it appears vice versa (214).

This neutrophil/macrophage interplay can be considered a crucial factor in cancer growth and prognosis as multiple studies have reported a strong association with the role of M2-like TAM phenotypes and oral cancer aggressiveness (215–219). One of the predominant TAM markers that is correlated with a poor clinical prognosis is CD163 (220). Indeed, an increase in CD163 expression is seen in advanced OSCC compared with premalignant lesions (218, 221) and initial tumor stages (216, 220). The ratio between CD163^+^ and CD68^+^ (pan macrophage marker) increases in oral cancer with lymphogenic metastasis (222). Another TAM marker, CD206, was correlated with cancer aggressiveness and clinical prognosis (219). The significance of CD206 is evident, as a radiotracer specific to CD206 is clinically used to identify sentinel lymph nodes in oral cancer patients to aid in OSCC diagnosis and treatment decisions (223).

TAMs promote tumor progression in several different ways. TAMs not only directly provide structural support for cancer development but also contribute to tumor induction by producing signaling molecules and extracellular vesicles. These vesicles play a significant role in crosstalk between cells by transferring bioactive cargo such as microRNAs (miRNAs) to recipient cells (224). TAMs can also directly communicate with tumor stem cells to support their survival by secreting growth factors, and in return, tumor stem cells provide essential tumor-promoting signals to activate TAMs that promote tumorigenesis (225). Furthermore, TAM-secreted cytokines induce anti-apoptotic programs in cancer cells (226–228). Following activation of the STAT3 pathway due to TAM-derived IL-6, tumor suppressor miR-204-5p expression significantly decreased, increasing in the anti-apoptotic protein RAB22A and B-cell lymphoma 2 (Bcl2) expressions in cancer cells (229–231). Thus, TAMs can enhance cancer cell resistance to chemotherapy and radiotherapy.

Another crucial role of TAMs in cancer is metastasis. TAMs allow tumor cell invasion and migration *via* cathepsins, secreting matrix metalloproteinases (MMP) and serine proteases, which alter cell–cell junctions and disturb basal membranes (232). TAMs either directly or indirectly inactivate T-cell responses or facilitate immune escape within tumors (233). Direct strategies include (i) depletion of metabolites essential for T-cell proliferation such as L-arginine, which is necessary for T-cell fitness and anti-tumor activity, through the expression of arginase-1 (ARG1), (ii) production of reactive oxygen species (ROS), (iii) expression of immune checkpoint ligands such as programmed cell death ligands (PDL1 and PDL2), cytotoxic T-lymphocyte-associated protein 4 (CTLA4) (B7-1 and B7-2) and B7-H4, and (iv) producing anti-inflammatory cytokines such as IL-10 and TGF-β (209, 234). These well-known macrophage mechanisms of T-cell inactivation are present in neutrophils, which are known to express ARG1, ROS, PDL1, and IL-10 (235, 236). It must be noted here that there is debate over whether human neutrophils produce IL-10. However, Lewkowicz et al. (237) have shown that in inflammatory settings, human neutrophils do produce IL-10. Though TAMs predominantly present pro-tumorigenic roles, the plasticity of macrophages has been used in a breast cancer model to re-program pro-tumorigenic TAMs (238). It has been shown that upon treatment with the class IIa histone deacetylase inhibitor, TMP195, TAMs become activated and reprogrammed to an extremely phagocytic phenotype, resulting in a reduction in tumor volume (238).

Clinical studies have indicated a link between the recruitment of TAMs and poor overall survival in OSCC patients and suggest this could be used as a potential prognostic marker (239, 240). Immuno-histochemistry analysis of OSCC indicated considerable TAM infiltration compared to control samples and the existence of CD68^+^CD163^+^(M2) TAMs or CD206^+^ (M2-like) TAMs were linked with poor overall survival (241–243). Additionally, CD163^+^ (M2) TAMs are linked with chemoresistance in esophageal cancer (244) and primary HPV-negative HNSCC (245). TAMs can adopt an extensive range of diverse activation states between M1 (classical) and M2 (non-classical), expressing both M1 and M2 markers, upregulated TNF-α (M1) (246), matrix metallopeptidase 9 (MMP-9) (M1) (246), increased levels of CCL2, CCL5, CXCL9, CXCL10, and CXCL16 chemokines (M1) (247), upregulated IL-10 (M2) (248), arginase-1 (Arg1) (M2) (249), and peroxisome proliferator-activated receptor γ (PPARγ) (M2) (250). Though the overall number of TAMs accumulated within a tumor is not considered in the assessment of clinical prognosis, the ratio of M1/M2 is considered an important prognostic marker (251, 252). A clinical cohort study showed that high expression of receptor for activated C kinase 1 (RACK1) inhibits macrophage recruitment and decreases the M1/M2 ratio (tumor having a higher M2 proportion) *via* the NF-KB pathway, promoting the development of OSCC, indicating RACK1 and the M1/M2 ratio are predictors of a poor prognosis in (253). The increased understanding of the role of TAMs in carcinogenesis is reflected across many immune cells in the TME and, similarly, the N1/N2 ratio is currently being investigated as a prognostic marker and, along with the NLR ratio, there may come a point whereby we can use several cell-based ratios to inform more accurate treatment and prognostic outcomes.

## Crosstalk Between Dendritic Cells and Neutrophils

Upon activation by numerous inflammatory stimuli, neutrophils release several inflammatory proteins (e.g., TNF-α) (254) and different alarmins such as defensins, cathelicidins (LL-37), lactoferrin, and high-mobility group box-1 (HMG-B1), with the ability to stimulate the maturation of immature DCs (255–258). Alarmins induce the maturation of immature DCs and their recruitment at the site of inflammation by acting on Giα-protein-coupled-receptor (GiαPCR) and activating receptors and also by stimulating the production of chemokines by leukocytes (259). It has been shown that neutrophil derived α-Defensins, human neutrophil peptide-1 and -2 (HNP-1 and -2), contribute to adaptive immunity by mobilizing DCs and T cells (260). β defensins secreted by neutrophils bind to TLR-4 receptors expressed on immature DCs, promoting their maturation and the initiation of adaptive immunity (261). HMG-B1 induces the migration and activation of immature DCs, leading to DC stimulation of T-cell proliferation and T helper 1 polarization (262). This DC initiation of a T-cell response is reliant on the binding of neutrophil Mac-1 and CEACAM1 (carcinoembryonic antigen-related cellular adhesion molecule-1 or CD66a) to the DC-specific receptor, DC-SIGN, resulting in the delivery of activation signals and antigenic molecules to DCs and the initiation of a T-cell response (263, 264). This cellular adhesion can also regulate neutrophil proliferation and prolong the survival of neutrophil granulocytes (265, 266).

**Figure 3:**
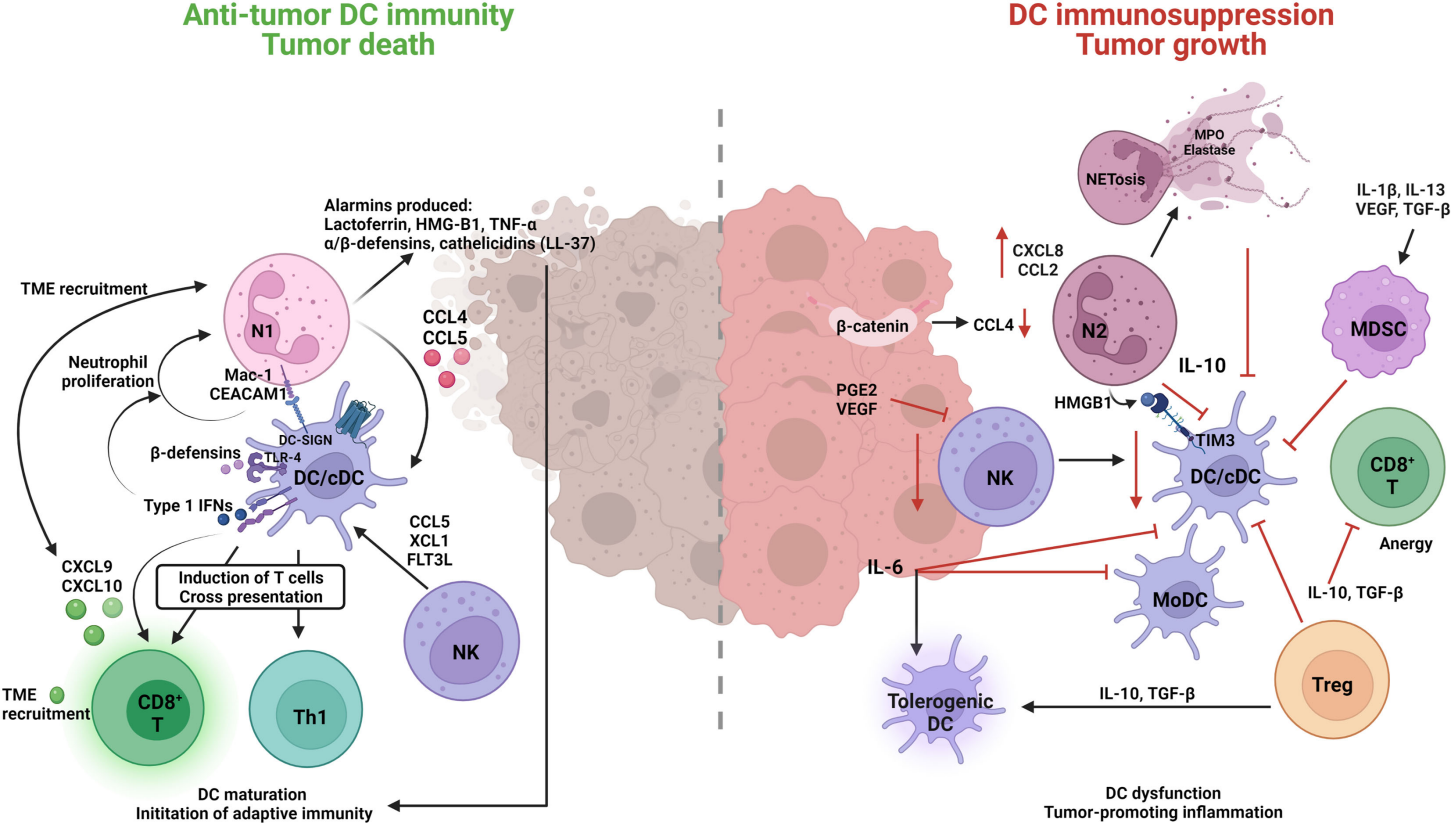
Neutrophil engagement with dendritic cells in the TME can result in immune-suppressive or immune-promotion of cancer pathology. Classical/conventional DC1 cells (cDC1) are the predominant subtype orchestrating an anti-tumor response through the interplay with N1 TAN, CD8^+^ cytotoxic T (CD8+ T) and natural killer (NK) cells. N1s produce several alarmins and cytokines that induce DC maturation, TME recruitment, and the initiation of anti-tumor adaptive immunity. DCs in-turn induce N1 proliferation and survival through cytokine/chemokine release, Type l IFNs, *β*-defensins and direct interaction MAC-CEACAM1/DC-SIGN receptor engagement. N1-induced DCs have a greater propensity to engage with and enhance the functions of anti-tumor CD8^+^ T cells, Th1, and NK cells to induce cytotoxic killing of tumor cells. On the other hand, N2 TANs and MDSCs have a suppressive role on DC functions and promote tumorigenic tolerant DCs. Reduction in N2 produced CCL4 leads to decreased cDC tumor infiltrate, an βincrease in N2 CXCL8 and CLL2 contributes to tumor progression and invasion pathways, N2–DC cell interaction HMGB1-TIM3, and IL-10 production leads to inhibition of cDCs. The inhibition of cDCs further compromises other anti-tumor immune cells (NK, CD8^+^ T) and allows for suppressive cells (N2, MDSC, Treg, tolerogenic DCs) to promote tumor growth.

Accumulating evidence indicates that DCs play a significant role in driving immune suppression against tumor-associated antigens (**Figure 3**) (267). The migration of DCs is critical for tumor immune surveillance (268). This includes DCs migrating to tumor sites, capturing and endocytosing dead tumor cells or cellular debris, and transporting tumor-associated antigens to tumor draining lymph nodes (TDLNs), where they induce tumor-specific T-cell activation (269). DC recruitment to the TME relies on chemokines such as CCL4, CCL5, and XCL1, while CCR7 is required for migration of DCs to TDLNs (268). Neutrophils are known producers of CCL4 and CCL5, so they would contribute to DC recruitment to the TME (270). Generally, it is assumed that informative signals within the TME program DCs into a tolerogenic or immunosuppressive state rather than an inflammatory state (271, 272). The infiltration of BDCA3^+^ cDC1s in the TME has been shown to be associated with greater T-cell infiltration, improved prognosis in cancer patients, and better efficacy of cancer immunotherapies (273), emphasizing the important positive role of cDC1s in generating antitumor immune response in the TME.

Antitumor immunity has been found to be extremely dependent upon expression of the type I IFN receptor (IFNAR1) (274). Thus, administration of type I IFNs (IFN-α, IFN-β) is considered a treatment strategy in cancer (275) as they facilitate DC activation, migration, and cross-presentation, thus enhancing the DC anti-tumor immunity (276). Type I interferon treatment may also have additional benefits as type I IFN treatment will aid neutrophil antitumor activity by polarizing them to the N1 phenotype (146). In an *in vitro* study it was demonstrated that sensing of nucleic acids through the cyclic-GMP-AMP synthase (cGAS)-stimulator of the interferon genes complex (STING) pathway and interferon regulatory factor 3 (IRF3) contributes to DC activation and IFN-β production in antitumor immunity (277). DCs can also facilitate the trafficking of effector T cells into tumors by producing certain chemokines. For instance, CD8^+^ T-cell recruitment into the TME is mediated through the chemokines CXCL9 and CXCL10, which are produced by tumor-infiltrating cDC1s (278). Neutrophil migration has been shown in mice and humans to be induced *via* a CXCR3–CXCL9 and CXCR3–CXCL10 axis (279–281). Thus, cDC1s would also recruit neutrophils, and as neutrophils are also major producers of these two chemokines, there would be positive reinforcement of CD8^+^ T cells and further neutrophil infiltration into the TME (270).

The TME comprises a range of immunosuppressive factors known to inhibit DC antitumor activity and infiltration, promoting immune tolerance and tumor progression (282). A high concentration of cDC1s within the TME has been correlated with good prognosis. However, tumor cell-intrinsic factors can limit cDC1 recruitment (268). It has been shown that active β-catenin in TME induces low CCL4 expression, leading to a significant reduction of cDC1 infiltrate and consequently an increase in tumor growth (283). Additionally, depending on the release of pro-inflammatory mediators, e.g., cytokines and granule contents by neutrophils-through NETosis or degranulation, neutrophils may either suppress or promote T-cell activation in the context of cancer immunity (284). For instance, the release of lactoferrin promotes the recruitment and activation of DC (285), while myeloperoxidase (MPO) and elastase, which are abundantly expressed by neutrophils, have a suppressive impact on DC migration and activation, although the role of neutrophils here is to be elucidated (286). In contrast, tumor-infiltrating NK cells have induced cDC1s recruitment by CCL5 and XCL1 production (282), and promote cDC development and proliferation, with FMS-like tyrosine kinase 3 ligand (FLT3L) (287). However, tumor cells can produce PGE2, which reduces FLT3L-producing NK cells and pro-inflammatory chemokine production. This in turn reduces cDC1 infiltration and the terminal differentiation of pre-DCs, resulting in tumor-promoting inflammation (288).

Cancer cells secrete IL-6. Although a pro-inflammatory cytokine, it reduces cDCs and MoDCs differentiation and promotes tumor DC dysfunction (289, 290). A dual function of IL-6 and M-CSF in tumor promotion is that they inhibit CD34^+^ progenitor differentiation into DCs but then induce their commitment towards CD14^+^ monocytes with an effective phagocytic capability but lacking APC functionality, thus failing to mediate allogeneic T-cell proliferation (291). Tumor-derived IL-6 is reported to be involved in the induction of tolerogenic DC phenotypes (292), but can switch the monocyte differentiation to macrophages rather than DCs (293). Several factors, such as IL-1β, IL-13, vascular endothelial growth factor (VEGF), and transforming growth factor beta (TGF-β) that are secreted by TME tumor cells, inhibit cDC maturation and survival and promote their differentiation into immunosuppressive cells, e.g., tumor-associated macrophages (TAMs) and myeloid-derived suppressor cells (MDSCs) (294). In particular, VEGF can inhibit FLT3 ligand (FL) activity and suppress cDC differentiation (295). Treg cells are commonly found in the TME and produce IL-10 and TGFβ, which are two potent immunosuppressive cytokines resulting in DC dysfunction (296). Additionally, neutrophils have been shown in OSCC patients to express IL-10, indicating they would also contribute to DC dysfunction (297). IL-10 inhibits several aspects of DC biology, including DC maturation, IL-12 production, and antigen presentation to T cells (298). Further, it has been shown that IL-10 provokes a switch from an immunogenic DC profile toward a tolerogenic DC state and the induction of antigen-specific anergy in cytotoxic CD8^+^ T cells (299). Further, Treg produced TGF-β can inactivate DC function by inhibiting DC maturation (300).

The process of apoptotic cell death plays an important role in determining immunogenicity as it induces the activation of cDCs and primes humoral and/or effector T cell-mediated immune responses (immunogenic cell death) (301). Immunogenic cell death depends on the alarmin high mobility group protein B1 (HMGB1) (302) as it binds nucleic acids released from dying tumor cells in the DC endosome, facilitating innate sensing of dead tumor cell nucleic acids (303). However, these processes are often inhibited in tumor-infiltrating cDCs through high expression of the inhibitory receptor T-cell immunoglobulin and mucin domain 3 (TIM3), which interacts with alarmin HMGB1, inhibiting anti-tumor responses and reducing the efficacy of cancer treatments (304, 305). In patients with resectable non-small cell lung cancer, the ratio of CD66b^+^ tumor-infiltrating neutrophils (TINs) to CD8^+^ T cells is reported as an independent prognostic factor for high tumor recurrence and poor overall survival (306). In a recent study conducted in lung adenocarcinoma, it has been shown that CD66b^+^ TIN infiltration significantly correlated with TIM3 expression (307). It has been shown that antibody crosslinking of TIM-3 results in tyrosine phosphorylation and the activation of the nonreceptor tyrosine kinases, Bruton’s tyrosine kinase (Btk) and c-Src, which then suppress DC activation and maturation *via* inhibition of the NF-κβ pathway (308). These studies emulate the diversity and complexity of tumor immunity and how several immune cells are interconnected to produce a single outcome, which needs further work to be fully elucidated.

CD47, a transmembrane protein known as a “do not eat me” signal, which is highly expressed on tumor cells, interacts with signal regulatory protein α (SIRPα) expressed on dendritic cells (309). Engagement of SIRPα by CD47 promotes the phosphorylation of the immunoreceptor tyrosine-based inhibitory motif (ITIMs) in the cytoplasmic tail of SIRPα, which in turn recruits SHP-1 and/or SHP-2 [src homology-2 (SH2)-domain containing protein tyrosine phosphatases] to dephosphorylate motor protein myosin IIA, thus preventing phagocytosis (310). Abundant expression of CD47 has been associated with poor survival in several types of cancers (311). DCs are more dedicated in employing cytosolic DNA sensing pathways to connect innate response to adaptive response following anti-CD47-mediated phagocytosis (310, 312). It has been shown that blockade of CD47 facilitated the activation of NADPH oxidase NOX2 in DCs, which in turn prevented phagosomal acidification and decreased the degradation of tumor mitochondrial DNA (mtDNA) in DCs (312). A recent study has shown that oxidized mtDNA from irradiated cancer cells can translocate to the cytosol of dendritic cells (313), activating the STING (stimulator of interferon genes)-TBK1 (TANK-binding kinase 1)-IRF3 (transcription factor interferon regulatory factor 3)-IFN-β pathway enhancing antigen cross-presentation, CD8^+^ T-cell activation and antitumor immunity and resulting in tumor rejection (312, 313). Tumors are known to produce colony-stimulating factor-1 (CSF-1) which recruits TAMs, which in turn inhibit DC maturation (314). Additionally, it has been shown that CSF1 producing neutrophils mediate immunological tolerance by promoting the development of proliferating Ly6C^lo^ macrophages with suppressive function (208). Tumors can also induce DC dysfunction *via* altering DC metabolism, for example, by increasing the accumulation of truncated fatty acids such as triglycerides in DCs (315). It has also been shown that a high lipid content within DCs reduces their ability to activate allogeneic T cells or present antigens, indicating cancer immune responses can be manipulated, positively or negatively, by altering the lipid levels in DCs (315).

Several signaling pathways such as β-catenin, signal transducer and activator of transcription (STAT), and mitogen-activated protein kinase (MAPK) trigger multiple immunosuppressive cascades in cancer (316). In addition to these signaling pathways, the Wnt signaling pathway is emerging as having a fundamental role in shaping the functions of DCs in the TME (317–319). Currently, nineteen Wnt proteins (lipid-modified cysteine-rich glycoproteins) typically 350–400 amino acids in length and ten cognate Frizzled (Fzd) receptors have been identified in humans (320). It has been reported that the Wnt family of ligands is highly expressed in the TME and that different tumor types have different composition profiles of Wnt proteins (320). For instance, Wnt1 is highly expressed in lung adenocarcinoma (321), while in melanoma (322), and oral carcinogenesis (323), high expression of Wnt3a and Wnt5a are found. In addition to affecting DCs in the TME, Wnt3a and Wnt5a, albeit not directly shown in cancer, affect neutrophil maturation and recruitment, with Wnt5a being shown to act as a chemoattractant and induce CXCL8 and CCL2 from neutrophils, two chemokines recently implicated in OSCC progression and invasion (324–328).

## Extracellular Matrix (ECM) and Cancer-Associated Fibroblasts (CAFs)

In cancer, the extracellular matrix (ECM) is a non-cellular network consisting of macromolecules such as collagen, fibrous structural proteins, glycoproteins, growth factors, and proteoglycans that provide structural and biochemical support to surrounding cells (329). The formation of deregulated and disorganized ECM results in the promotion of malignant cell transformation (330). Several proteases released by neutrophils can contribute to the continuous remodeling process of the ECM and mediate immune responses (331). In the context of cancer, neutrophils release neutrophil elastase (NE) in large quantities that, through its influential protease activity, can cleave not only elastin but also other extracellular matrix proteins such as collagen, laminin, and numerous transmembrane proteins, which devastate the firm junctions between cells and provoke the exudation and migration of neutrophils (332, 333). In addition, boosted NE activity activates matrix-metalloproteinases (MMPs), which may improve the degradation of ECM and cause tissue damage (334). It is assumed that upregulation of neutrophil-derived MMP-8 and MMP-9 can degrade lung structure proteins such as collagen and elastin to produce bioactive peptides that stimulate neutrophil chemotaxis through CXCR1/2 receptor activation, supporting the occurrence of inflammatory cascades (335, 336). In an inducible colon tumor mouse model, neutrophil-secreted MMP-9 stimulates latent TGF-β in the ECM by damaging the ECM, enhancing TGFβ in the TME, and resulting in suppressing the antitumor T-cell response (337). Cathepsin G, a serine protease secreted by activated neutrophils, promotes E-cadherin/catenin complex formation on fibronectin and thereby induces cell–cell adhesion of MCF-7 human breast cancer cells, suggesting that cathepsin G plays a role in tumor development and metastasis (338).

Collagen, laminin, and fibronectin are the main ECM proteins involved in HNSCC development and progression (339). Immunohistological studies of different histological grades of HNSCC indicated a direct relationship between the presence of collagen/or laminin and the degree of differentiation of oral squamous cell carcinoma (340, 341). A decreased distribution of ECM proteins was positively associated with increasing cancer stages, with the deposition of collagen or laminin decreasing with higher histopathological grades and an absence of staining associated with a poor prognosis (342). Coculturing UMSCC47 cells (OSCC cell line) and neutrophils was shown to increase UMSCC47 invasion and matrix degradation (343). In highly invasive primary OSCC tumors, the expressions of laminin, collagen type IV, and vitronectin were decreased. In contrast, the expressions of fibronectin and tenascin were increased, indicating that the composition of ECMs in OSCC is valuable in predicting tumor behavior (344).

**Figure 4:**
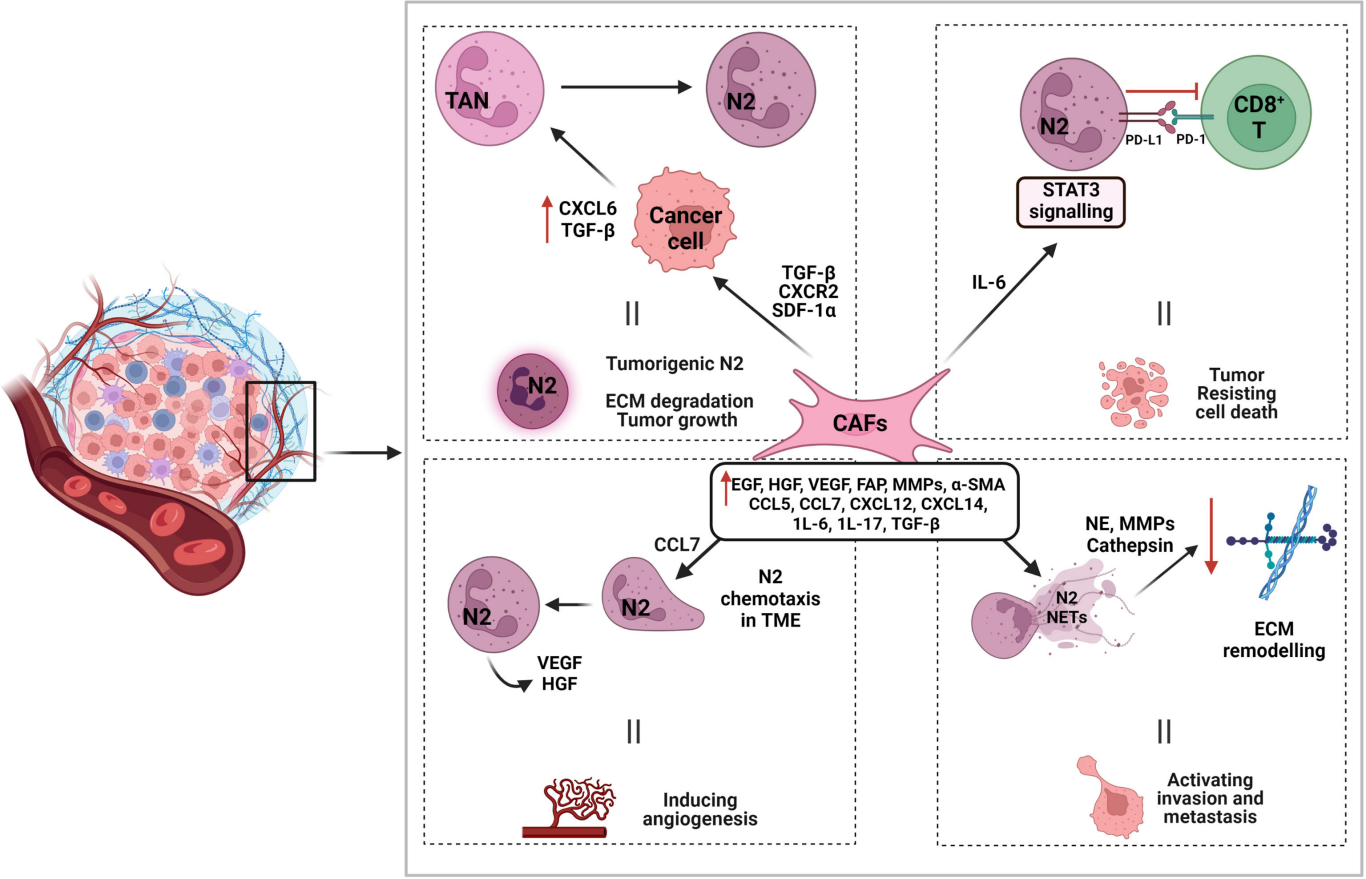
Interaction of cancer-associated fibroblasts in promoting N2 function and tumorigenesis. Cancer-associated fibroblasts (CAFs) induce tumorigenic N2 by various interactions. CAF associated molecules such as TGF-β, CXCR2, and SDF-1*α*, promote cancer cell expression of CXCL6 and TGF-β, which aid in N2 polarization and TME recruitment. CAF produced IL-6 induces STAT3 signaling pathways that modulates PD-L1/PD-1 interaction between N2 and CD8^+^ T cells and aids in tumor cell death resistance. CAF associated chemokine CCL7 also aids in N2 recruitment to TME by chemotaxis. Other CAF associated molecules contribute to N2 NETosis and the production of proteases such as NE, MMPs, and cathepsin to degrade and remodel ECM and promote tumor invasion and metastasis.

The main function of cancer-associated fibroblasts (CAFs) main function has been shown to be in preserving the microenvironment for tumor cell growth and proliferation *via* the secretion of a large variety of autocrine and paracrine cytokines and other tumor-promoting factors such as CCL5, CCL7, CXCL12, CXCL14, epidermal growth factor (EGF), hepatocyte growth factor (HGF), IL-6, IL-17, and VEGF (61, 345–348) (**Figure 4**). It has been shown that CCL5 is an effective inducer of neutrophil recruitment in septic lung injury through the formation of CXCL2 in alveolar macrophages (349). CCL7 generated by CAFs is the key promoter of OSCC cell migration and invasion, guides cytoskeletal transformation, and triggers membrane ruffling and cell dissemination (350). CCL7 exerts its carcinogenesic properties as a chemoattractant for neutrophils involved in the formation of the tumor microenvironment (351). It has been demonstrated that neutrophils are directly angiogenic by releasing VEGF and HGF (352).

It has been shown that CAFs influence the motility of cancer cells by inducing epithelial–mesenchymal transition (EMT) *via* secreted cytokines in endometrial cancer cells (353). The best markers used to identify CAFs in the TME are (i) α-smooth muscle actin (α-SMA), a specific marker of myofibroblasts (354) and (ii) fibroblast activation protein (FAP) (355). In a clinical study, α-SMA was upregulated and correlated with poor prognosis in oral carcinoma (356). Another study found that upregulation of FAP at the mRNA level in human tongue squamous cell carcinoma was also linked to poor prognosis (357). It has been demonstrated that an abundance of myofibroblasts leads to more aggressive behavior of the squamous cell carcinomas and is associated with a worse prognosis in HNSCC patients (358). A strong association between increased CAF density and higher mortality in mobile tongue squamous cell carcinoma has been reported (359). Numerous immunohistochemical studies have shown that HNSCC-derived CAFs express high levels of TGFβ, hepatocyte growth factor, and MMPs compared with healthy fibroblasts (360–362). In the context of the TME, blockade of TGF-β results in the recruitment and activation of TANs with an antitumor phenotype, indicating a major role of TGF-β in tumor promoting N2 polarization (121). This recruitment of neutrophils upregulates the expression of MMP9 and MMP-9^+^ neutrophils play a functional and concomitant role in tumor cell angiogenesis and intravasation (363).

CAFs might be able to modulate the polarization of TANs. A recent study showed that CAF-derived cardiotrophin-like cytokine factor 1 (CLCF1) induces TAN-N2 polarization by increasing the expression of CXCL6 and TGF-β in tumor cells, thereby accelerating tumor progression (364). Another study showed that CAFs recruit neutrophils to tumors by producing stromal cell-derived factor 1 (SDF-1α, known as CXCL12) (365). Furthermore, CAFs enhance TAN recruitment in a CXCR2-dependent manner (366). CAF-derived IL-6 induces the activation of STAT3 pathways in TANs, which are essential for the survival and function of activated neutrophils, subsequently suppressing T-cell immunity and inducing immune tolerance in a PD1/PDL1-dependent manner within the TME (364). This interaction of CAFs and neutrophils needs to be explored to understand how cancer progresses *via* this interaction and the possibilities to disrupt this mechanism through therapeutic targeting.

## Interaction of Natural Killer (NK) Cells and Neutrophils

Natural killer (NK) cells were first identified as a subpopulation of innate lymphoid cells (ILCs) and comprise about 5–15% of the total peripheral blood mononuclear cells (PBMCs) (367). Though NK cells and ILCs are derived from a common progenitor cell, NK cell development depends on IL-15-mediated signaling, whereas IL-7 signaling induces ILC differentiation (368). NK cells are considered the most efficient immune cells involved in immunosurveillance as they can target infected or cancer cells lacking major histocompatibility class I (MHC-I), marking them for programmed cell death (369). In fact, NK cells mainly target cells with low MHC-I expression or cells that express the cell stress markers MIC-A or MIC-B (370). In contrast, in healthy cells, the binding of MHC-I molecules to their receptors on NK cells blocks NK cell function (371). In a large OSCC cohort study, it was found that CD57^+^ NK expression is positively associated with high tertiary lymphoid structures (TLS), indicating higher overall survival rates (372).

**Figure 5:**
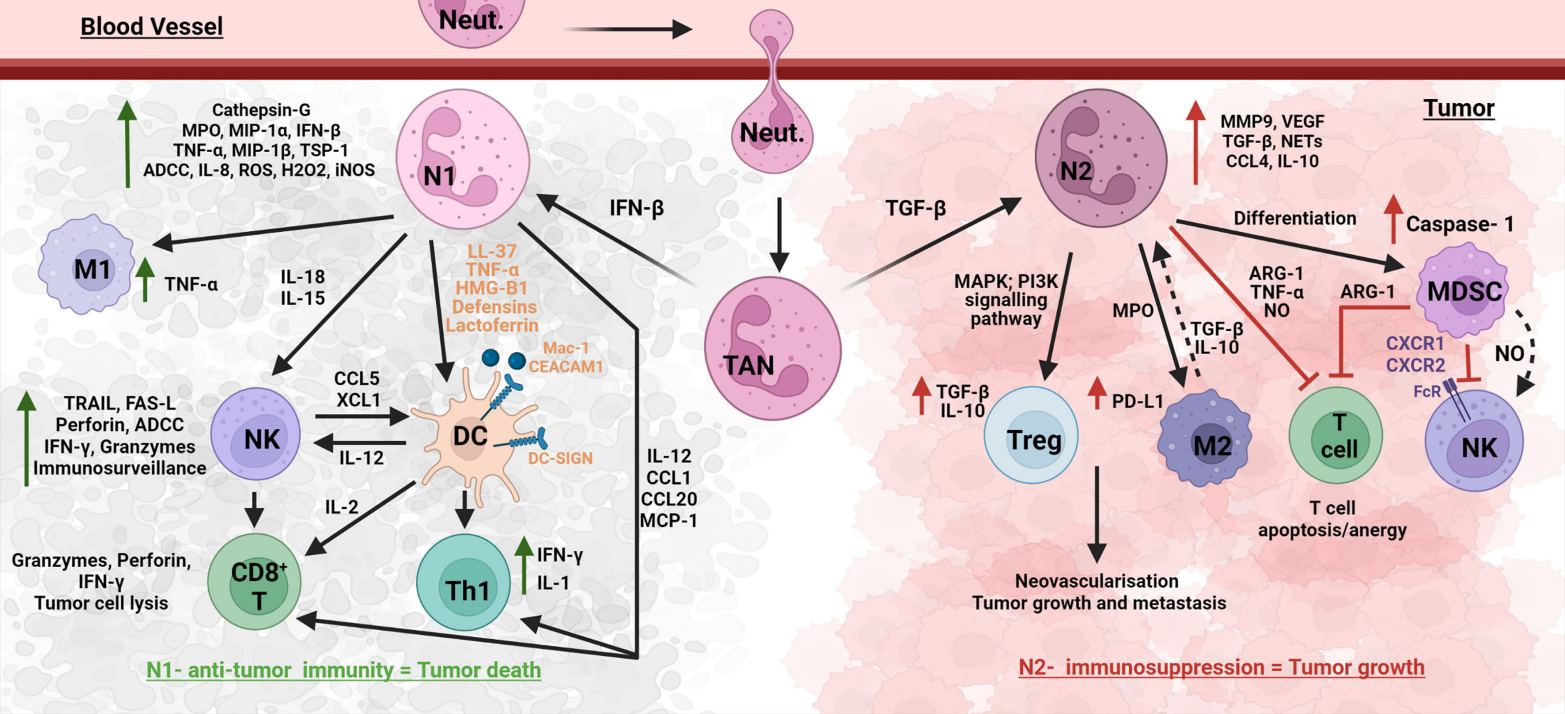
Neutrophil networks affecting oral cancer outcomes. Neutrophils interact with anti-tumor and suppressive immune cells in the complex TME ecosystem. TME recruited neutrophils, TANs (tumor-associated neutrophils) polarize to a N1 anti-tumor phenotype in the presence of IFN-β, and to a N2 tumorigenic phenotype with TGF-β. N1 neutrophils upregulate several molecules such as cathepsin-G, MPO, MIP-1 *α*/β, IFN- β, TNF-*α*, IL-8, and TSP-1, to induce other immune cells and also execute N1 functions such as ADCC, ROS, and iNOS-induced cytotoxicity. N1 induce M1 TAM, NK (IL-18, 1L-15), T cells (IL-12, CCL1, CCL20, MCP-1) and DCs (LL-37, TNF-*α*, HMG-B1, defensins, lactoferrin, cell interaction Mac-1/CEACAM1–DC-SIGN). DCs further aid in N1 induced NK *via* IL-12, and NK aid in N1 induced DC *via* CCL5 and XCL1. N1 induced DCs further promote CD8^+^ T cells and Th1 cells. Interaction of these anti-tumor immune cells with N1 promotes tumor death. On the other hand, N2 neutrophils promote suppressive cells such as MDSC, Treg, and M2 and function *via* molecules such as MMP-9, VEGF, TGF-β, NETs, CLL4, and IL-10. N2 and MDSC inhibit anti-tumor T cells *via* Arg-1, TNF-*α*, and NO; and NK cells *via* NO and CXCR1/2. These lead to tumor growth and metastasis.

NK cells are a heterogeneous population and in mice are recognized as CD3^−^ NKp46^+^ or more commonly as CD3^−^ NK1.1^+^ lymphocytes. In humans, NK cells have been categorized into two distinct subpopulations: immature CD3^−^ CD56^bright^ CD16^−^ cells and mature CD3^−^ CD56^dim^ CD16^+^ cells (373). Immature and mature NK cells differ in their functions and have different sensitivities to activating cytokines. After activation by IL-2, IL-15, and IL-12, immature NK cells can activate systemic antitumor immunity indirectly by modulating the function of other innate and adaptive immune cells *via* the secretion of several cytokines such as IFN-γ, TNF-α, GM-CSF, and chemokines such as CCL1, CCL2, CCL3, CCL4, CCL5, and CXCL8 (374). It has been shown that neutrophil-derived IL-18, along with dendritic cell-produced IL-12, is critical for IFN-γ synthesis by NK cells, indicating that neutrophils are essential activators of NK cells (**Figure 5**) (375). In patients with severe congenital neutropenia, the percentage of responding NK cells is much lower in comparison with healthy control patients, indicating the significant role of neutrophils in NK cell maturation and function (376).

The majority (90%) of NK cells in PBMCs, which show less response to cytokine stimulation, are mature cells. Mature NK cells have several direct cytolytic mechanisms against tumors and pathogen-infected cells, which include (i) lysis by cytolytic granules such as granzyme and perforin, (ii) death receptor (DR) mediated apoptotic processes such as TNF-related-apoptosis-inducing-ligand (TRAIL)/TRAIL receptors or induction of apoptosis by FasL/Fas ligation, and (iii) antibody dependent cell-mediated cytotoxicity (ADCC) (377, 378). In tumor models, the cytotoxic activity of NK cells has been shown to be inhibited by the cell–cell interaction with MDSCs (pathogenically activated neutrophils), reducing NK cell activation by IL-2 and perforin production and a significant decline in the ability of NK cells to attack tumor cells (379). MDSC-derived nitric oxide impairs NK Fc receptor binding, leading to reduced ADCC and impaired signal transduction (**Figure 5**) (380). In HNSCC patients, inhibition of MDSC trafficking with SX-682, a small-molecule inhibitor of CXCR1 and CXCR2, enhances NK cell immunotherapy, indicating the important role MDSCs play in NK cell function in TME (381).

There has been improving evidence that neutrophil‐derived mediators modulate NK cell effector functions in humans and mice, and in return, NK cells can modulate the survival, recruitment, and functional responses of neutrophils (**Figure 5**) (376, 382). In a murine colon cancer model (383), the association between the tumor infiltrate of the neutrophil and NK cell-mediated antitumor immunity was investigated. It was demonstrated that there was crosstalk between neutrophils and NK cells as neutrophil depletion significantly (i) decreased the frequency of IFN-γ^+^ cells within NK cells, (ii) increased the fraction of Ki67^+^ NK cells, and (iii) increased the fraction of dead NK cells, indicating that neutrophil depletion during homeostatic proliferation induced NK cell proliferation accompanied by poor survival of NK cells (383). It has also been reported that some cytokines from neutrophils (such as IL-5 and IL-18) are involved in NK cell activation or support the survival of NK cells. IL-15, expressed in granulocytes including murine and recently human neutrophils (384), mediates a wide range of effects on mouse NK cells and is considered an important cytokine for NK cell maintenance (385) and homeostatic proliferation (386). It is interesting to note here that the Chen et al. study (384) detected IL-15 *via* mRNA expression (rather than secreted protein) from human neutrophils that were under a highly inflammatory condition (sepsis), indicating that neutrophils may have an inflammatory phenotype yet to be elucidated and this may have a significant impact on cell-to-cell engagement. IL-15 and IL-18 in synergy with IL‐12 produced from dendritic cells are also required for IFN-γ expression by NK cells (387–389). The proinflammatory heterodimer S100A8/A9 which is constitutively expressed by myeloid cells, including neutrophils, has been shown to directly enhance the cytotoxic activity of NK cells through binding to the receptor for advanced glycation end products (RAGE) (390).

Neutrophil-derived molecules such as azurocidin, cathepsin G, Defensins, elastase, and lactoferrin enhance NK cytotoxic activity in humans (391–393). NK cell cytolytic activity, instead of using an antigen-specific mechanism, is mediated by a broad repertoire of receptors which are engaged by ligands expressed on putative target cells (394). These receptors can be categorized either by their functions as NK cell-activating receptors and NK cell-inhibitory receptors or by their structure as Killer cell Lectin-like Receptors (KLRs) and Killer cell Immunoglobulin-like Receptors (KIRs) (395). Each NK cell typically expresses only a selection of these receptors, and thus NK-cells are quite heterogeneous and have a diverse repertoire of different MHC class I specificities (396). Stimulatory and inhibitory receptor signaling regulate NK cell activation and the balance between these two signals controls the outcome of the interaction with the target cell (397). Normal cells are shielded from killing by NK cells when signals provided by activating ligands are balanced by inhibitory signals delivered by self-MHC-I. In contrast, cells experiencing stress, such as tumor cells, downregulate their MHC-I expression, a ligand for inhibitory receptors. Simultaneously, they develop stress-associated molecules, which act as ligands for activating receptors. Consequently, the absence of inhibitory signaling along with the induction of activating signaling shifts the balance toward NK cell activation, resulting in cytokine secretion and killing of tumor cells. This process is known as missing-self recognition (397, 398). A clinical study indicated that the rise in the expression of CD57^+^ NK cells in the tumor stroma of OSCC may serve as a good prognostic marker for the patients (399).

Monomorphic MHC-like molecule, CD1d-restricted T cells are known as NKT cells, which can be divided into two subsets based on their TCR repertoire and lipid antigenic profile specificity; type I and type II (400). Type I NKT cells play a significant role in regulating immune responses, including immune surveillance against tumors following stimulation by exogenous factors such as IL-12 or α-GalCer (401, 402). The NKT cell and neutrophil relationship has been investigated in hepatitis (403), renal ischemia–reperfusion injury (404), and pneumonia (405), and these studies showed that excluding or blocking NKT cells relieved the injury and reduced neutrophil infiltration (406). It has been shown that colitis-associated colorectal cancer was suppressed in NKT cell-deficient CD1d^−/−^ mice (406). This study suggested that NKT cells essentially act as an initiator, strongly expressing TNF-α which could stimulate epithelial chemokine secretion (CXCL1, 2, and 3), thereby mediating neutrophil recruitment indirectly. Neutrophils in turn become tissue-damaging through ROS upregulation (406), leading to colon cancer by causing DNA instability (407). It has been shown that iNKT cells can indirectly control tumor growth through targeting tumor-supportive, IL-6-producing, CD1d^+^ CD68^+^ tumor-associated macrophages (TAM) (408). Further, a deficiency in circulating iNKT cells was associated with poor clinical outcome in HNSCC patients, suggesting their critical contribution to antitumor immune responses (409, 410).

## Tumor Microenvironment (TME) in the Pathogenesis of HNSCC/OSCC

Though HNSCC is linked with intense immune suppression, the impact of the premalignant and TME on immune reactivity has yet to be elucidated. Significant infiltration of proinflammatory immune cells, such as CD163^+^ TAMs, CD8^+^ T cells, and NK cells, has been reported in oral leukoplakia and carcinoma (411–413). Using a mouse model of 4-nitroquinoline-1-oxide-induced oral carcinogenesis, De Costa et al. (414) investigated the shift in the immune cell phenotypes at the premalignant and malignant stages of HNSCC/OSCC (414). The development of oral premalignant lesions was shown to be associated with elevated levels of inflammatory Th1 cells, Type 1 CD8^+^ T cells (Tc1) secreting IFN-γ and Th17 cells compared with controls and HNSCC/OSCC-bearing mice, though the number of CD4^+^ regulatory T cells increased in HNSCC/OSCC-bearing mice (414). Regarding the inflammatory cytokine profile, it was shown that premalignant oral lesions are associated with an increased level of IL-17 as well as IL-23, in comparison with controls or HNSCC/OSCC, thus supporting the Th17 phenotype (415). In contrast, HNSCC tissues produce increased levels of TGF-β and skew normal spleen cells toward the Treg phenotype (415). Another study demonstrated that premalignant lesion cells released a panel of proinflammatory mediators including CCL5, G-CSF, monocyte chemoattractant protein 1 (MCP-1), and prostaglandin-E2 (PGE2) in comparison to HNSCC/OSCC cells, indicating that the premalignant microenvironment is more immune stimulatory than the microenvironment of an established HNSCC/OSCC (416). In addition, α-SMA (CAF cell marker) expression was high in premalignant lesions while it is not observed in normal epithelium (417).

The influx, differentiation, and activation of neutrophils in the TME is indicative of a functional interaction between NHSCC/OSCC cells and neutrophils. A study by Trellakis et al. showed that high infiltration of neutrophils in OSCC is associated positively with tumor stage and negatively with overall survival times (418). HNSCC/OSCC cancer cells directly recruit neutrophils, extend their survival, and stimulate their inflammatory activity. HNSCC/OSCC cells are reported to be a crucial trigger for the recruitment of neutrophils as high serum concentrations of the inflammatory/chemotactic chemokines CCL4, CCL5, and CXCL8 in HNSCC/OSCC patients (418). The interaction of neutrophils and HNSCC/OSCC cells was found to enhance the chemotaxis of neutrophils to the TME and secretion of MMP-9 and CCL4 by neutrophils, triggering further recruitment and aiding tumor progression (418). Elevated MMP-9 has been reported to be at the invasive front of squamous cell and verrucous carcinomas in the oral cavity, indicating that MMP-9 expression is a reliable marker for invasive squamous cell carcinoma grading (419).

The abundance of circulating MDSCs is observed in HNSCC/OSCC and is associated with advanced stages of cancer (420). Though inhibiting T-cell activation is a key function of MDSCs, both *in vitro* and *in vivo* studies have demonstrated MDSC-derived caspase-1 promotes the proliferation of HNSCC cancer in a T-cell-independent manner (421). The increase in MDSCs in HNSCC/OSCC patients has led to a few studies investigating methods to target these cells. A study by Weed et al. (422), showed that targeting MDSCs with tadalafil (10 mg/day) promotes antitumor immunity by increasing tumor-specific CD8^+^ T cells in a dose-dependent manner in patients with head and neck squamous cell carcinoma (422). Based on the observation that the expression of B7 homolog 3 protein (B7-H3) is an essential immunosuppressive mechanism in HNSCC, Mao et al. (423) conducted an HNSCC mouse model and showed that the blockade of B7-H3 decreased the levels of MDSCs and TAMs, as well as promoted IFN-γ secretion of cytotoxic T cells, resulting in enhanced antitumor immune activity (423). Another potential therapeutic target that may affect MDSCs is semaphorin 4D (Sema4D), a cytokine expressed by several epithelial malignancies and known to induce tumor angiogenesis produced by MDSC resulting in suppression of T-cell proliferation and IFN-γ production (424). Sema4D acts on immature MDSC and DC by (i) preventing their migration and (ii) inducing considerable increases in the immune-suppressive profile (425, 426). Although the MDSCs or neutrophils were not investigated, Zhou et al. (427) have shown that anti-Sema4D treatment reduced tumor growth and vascularization in an OSCC xenograft model, demonstrating a further possibility of targeting MDSCs.

A main contributor of inflammation in HNSCC is CD68^+^ TAMs, which is correlated with poor clinical outcomes in oral squamous cell carcinoma patients (428). Several studies confirmed that OSCC cells could directly suppress antitumor T-cell immunity through induction of PD-L1 expression on TAMs (429, 430). A high proportion of M2 macrophages express TGF-β and IL-10 in oral squamous cell carcinoma which was associated with a reduced patient survival time (431). TGF-β plays an important role in tumor-associated neutrophil polarization. It is shown that in the presence of TGF-β, neutrophils develop the N2 phenotype, which exhibits immunosuppressive and tumor-promoting activity, whereas blocking this molecule shifts the phenotype toward N1 (366). A recent study suggested that NET formation induced by TGF-β in oral lichen planus has substantial implications for developing oral cancer (432). The enhanced level of IL-10, which is produced by murine neutrophils (433), and inflammatory activated human neutrophils (237), leads to adverse survival in animal models and is associated with a poor prognosis in cancer patients (434). Genetic variation in IL-10, in particular IL-10 gene promote -1082 A/G (rs1800870) polymorphism, has been strongly associated with an increased risk of oral squamous cell carcinoma (435, 436) but has a non-significant association with HNC clinical stages and the association with neutrophils has not been conducted (437).

NK cells are well-known for their strong anti-tumor immunity, which is often compromised in cancer. Several studies investigating NK cells in oropharyngeal squamous cell carcinoma found that high abundance and activity of NK cells predicted improved survival, indicating that NK cells are a good prognostic marker for OSCC patients (399, 438). Tumor cells express NK activating receptor ligands *de novo*, making them susceptible to NK cell killing (439). The upregulation of the NK cell inhibitory ligand NKG2A on tumor-associated NK cells is considered one of the biological mechanisms of immune escape in HNSCC (440). NK cells from the primary tumor present a different phenotype than NK cells from the blood of the same HNSCC patients (399). Tumor-infiltrating NK cells significantly downregulated activating receptors such as NKG2D, DNAM-1, NKp30, CD16, and 2B4, while over-expressing their inhibitory receptors (e.g., NKG2A and PD-1) compared with matched blood NK cells and thereby could not kill target cells and produce cytokines (61, 399, 441). It has been reported that tumor-infiltrating NK cells reduce cytotoxicity and produce significantly less IFN-γ (442). In colitis, NKG2A-expressing NK cells reduce inflammatory neutrophil recruitment and functions. By extension, it could be possible that the presence of NKG2A^+^ NK cells in the TME would dampen N1 neutrophil anti-tumor activity (443, 444). With this phenotype, NK cells in the primary tumor would enhance tumor progression and immune suppression.

## Conclusion

Cancer such as HNSCC/OSCC is a major health issue globally and, with high incidence and mortality, it imposes a significant psychosocial and economic burden on individuals and society. Despite the clinical success of immunotherapies based on immune checkpoint inhibitors (i.e., antibodies against the immune regulators CTLA4 and PD-L1/PD-1), unfortunately, only a subset of patients respond to this treatment, suggesting that cancer immune evasion is a major barrier in current immunotherapy. An immune evasion characteristic that has received less attention is the crosstalk between distinctive immune cells within the TME and how this affects clinical outcomes.

**Figure 6:**
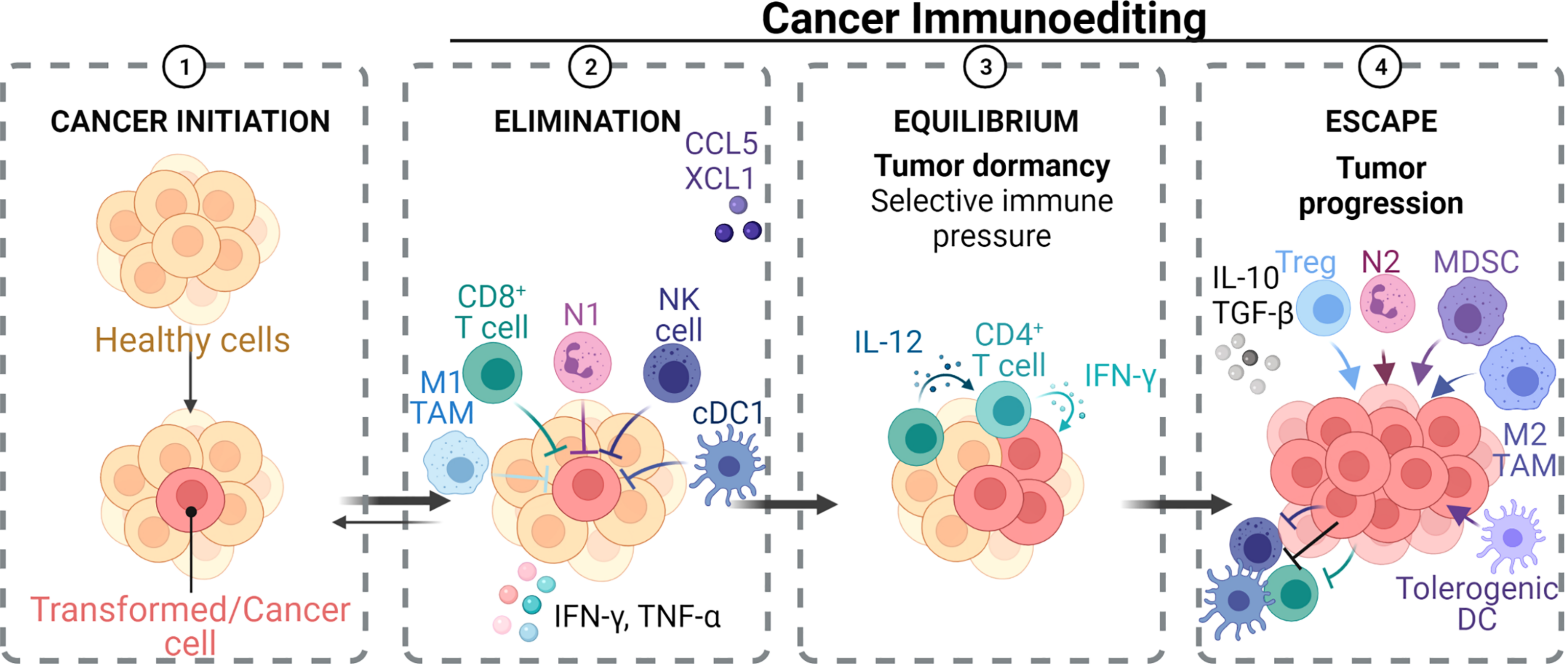
Immunoediting during tumorigenesis. 1. Initiation of cancer with transformation of healthy cells. 2. Robust response from innate and adaptive immune cells such as N1 neutrophils, CD8^+^ T, M1 TAM, NK, and cDC1, producing key cytokines IFNγ and TNFα to eliminate the cancer cells. 3. Equilibrium between the immune cell response and cancer growth in the presence of proinflammatory cytokines. Overtime, more resistant tumor variants arise that can evade the immune response and escape. 4. Tumor growth and progression in the presence of immune suppressive cells such as Tregs, N2 neutrophils, MDSCs, M2 TAMs, and tolerogenic DCs, producing key anti-inflammatory cytokines 1L-10 and TGF-β. Anti-tumor immune cells are suppressed in this highly tumoricidal environment.

Our review highlights that neutrophils do play a significant role in cancer immunology and that there are major holes in our knowledge of how neutrophils affect tumor immune escape mechanisms, and a better understanding of this will determine more appropriate biomarkers for diagnostics and treatment of cancer. However, the TME represents a complex eco-system which alters over time, and determining how tumor cells and other constituents of the TME, such as CAFs, B cells, DC, macrophages, MDSC, NK cells, and T cells interact with each other and neutrophils will be challenging (**Figure 6**). It can be seen that neutrophils have a role throughout cancer initiation and progression, recruitment of DC and NK cells and consequently T cells, N1s then the suppressive N2 and the formation of MDSCs (activated neutrophils) and their effects. Although originally maligned in cancer immunology due to their short half-life, it now appears that neutrophils play a significant if not pivotal role in cancer pathology through their heterogeneity phenotypes, N2, TANs, and MDSCs interacting with other myeloid immune cells to affect disease states and overall cancer survivability and treatment success (**Figure 5**). Thus, there needs to be a focus on neutrophils in cancer, which in turn may yield significant knowledge gain and lead to more effective and lasting treatments. Though current evidence supports a pro-tumor role for neutrophils in OSCC, there may be an anti-tumor role for neutrophils and there needs to be more research to elucidate the complexity of the neutrophil mechanisms involved in cancer. Understanding these immune cell interactions is crucial for a better understanding of the TME and in treatment provided to HNSCC/OSCC patients.

## Author Contributions

Conceptualization, SH and NO’B-S. Writing and original draft preparation, SH. Writing—review and editing, SH, NO’B-S, and BS. Figure drafting and editing, BS. All authors listed have made a substantial, direct, and intellectual contribution to the work and approved it for publication.

## Funding

The National Health and Medical Research Council (NHMRC) of Australia and Australian Research Council (ARC) are thanked for financial support over many years for the immunology, microbiology, peptide chemistry and chemical biology studies reported in the authors’ laboratories. NO’B-S is the recipient of NHMRC funding (APP1142472, APP1158841, APP1185426), ARC funding (DP210102781, DP160101312, LE200100163), Cancer Council Victoria funding (APP1163284), and the Australian Dental Research Foundation funding and research is supported by the Division of Basic and Clinical Oral Sciences and Centre for Oral Health Research at The Melbourne Dental School.

## Conflict of Interest

The authors declare that the research was conducted in the absence of any commercial or financial relationships that could be construed as a potential conflict of interest.

## Publisher’s Note

All claims expressed in this article are solely those of the authors and do not necessarily represent those of their affiliated organizations, or those of the publisher, the editors and the reviewers. Any product that may be evaluated in this article, or claim that may be made by its manufacturer, is not guaranteed or endorsed by the publisher.

## References

[1] Ferlay J Soerjomataram I Dikshit R Eser S Mathers C Rebelo M. Cancer Incidence and Mortality Worldwide: Sources, Methods and Major Patterns in GLOBOCAN 2012. Int J Cancer (2015) 136:E359–86. doi: 10.1002/ijc.29210 25220842

[2] Jou A Hess J. Epidemiology and Molecular Biology of Head and Neck Cancer. Oncol Res Treat (2017) 40:328–32. doi: 10.1159/000477127 28531899

[3] Chaturvedi AK Anderson WF Lortet-Tieulent J Curado MP Ferlay J Franceschi S. Worldwide Trends in Incidence Rates for Oral Cavity and Oropharyngeal Cancers. J Clin Oncol (2013) 31:4550–9. doi: 10.1200/JCO.2013.50.3870 PMC3865341 24248688

[4] Ghani WMN Ramanathan A Prime SS Yang YH Razak IA Abdul Rahman ZA. Survival of Oral Cancer Patients in Different Ethnicities. Cancer Invest (2019) 37:275–87. doi: 10.1080/07357907.2019.1635614 31307249

[5] Nakashima T Tomita H Hirata A Ishida K Hisamatsu K Hatano Y. Promotion of Cell Proliferation by the Proto-Oncogene DEK Enhances Oral Squamous Cell Carcinogenesis Through Field Cancerization. Cancer Med (2017) 6:2424–39. doi: 10.1002/cam4.1157 PMC5633549 28834425

[6] Vigneswaran N Williams MD. Epidemiologic Trends in Head and Neck Cancer and Aids in Diagnosis. Oral Maxillofac Surg Clin North Am (2014) 26:123–41. doi: 10.1016/j.coms.2014.01.001 PMC4040236 24794262

[7] Daraei P Moore CE. Racial Disparity Among the Head and Neck Cancer Population. J Cancer Educ (2015) 30:546–51. doi: 10.1007/s13187-014-0753-4 25398667

[8] Sathiasekar AC Mathew DG Jaish Lal MS Arul Prakash AA Goma Kumar KU. Oral Field Cancerization and Its Clinical Implications in the Management in Potentially Malignant Disorders. J Pharm Bioallied Sci (2017) 9:S23–s5. doi: 10.4103/jpbs.JPBS_109_17 PMC5731019 29284929

[9] Chin D Boyle GM Porceddu S Theile DR Parsons PG Coman WB. Head and Neck Cancer: Past, Present and Future. Expert Rev Anticancer Ther (2006) 6:1111–8. doi: 10.1586/14737140.6.7.1111 16831082

[10] Hadzic S Gojkov-Vukelic M Pasic E Dervisevic A. Importance of Early Detection of Potentially Malignant Lesions in the Prevention of Oral Cancer. Mater Sociomed (2017) 29:129–33. doi: 10.5455/msm.2017.29.129-133 PMC5544450 28883777

[11] Australian-Institute-of-Health-and-Welfare. Oral Health and Dental Care in Australia. Canberra: Australian Institute of Health and Welfare (2021).

[12] Mashberg A Samit AM. Early Detection, Diagnosis, and Management of Oral and Oropharyngeal Cancer. CA Cancer J Clin (1989) 39:67–88. doi: 10.3322/canjclin.39.2.67 2495159

[13] Sheikh MN Hanif S Zia M Qayyum Z. Effects of Nicotine on an *In Vitro* Reconstituted Model Oral Mucosa in Terms of Cytokine Production. J Ayub Med Coll Abbottabad (2011) 23:80–4. 23472421

[14] Hukkanen J Jacob P Benowitz NL. Metabolism and Disposition Kinetics of Nicotine. Pharmacol Rev (2005) 57:79–115. doi: 10.1124/pr.57.1.3 15734728

[15] Xue J Yang S Seng S. Mechanisms of Cancer Induction by Tobacco-Specific NNK and NNN. Cancers (Basel) (2014) 6:1138–56. doi: 10.3390/cancers6021138 PMC4074821 24830349

[16] Vineis P Alavanja M Buffler P Fontham E Franceschi S Gao YT. Tobacco and Cancer: Recent Epidemiological Evidence. J Natl Cancer Inst (2004) 96:99–106. doi: 10.1093/jnci/djh014 14734699

[17] Ogihara K Kikuchi E Yuge K Yanai Y Matsumoto K Miyajima A. The Preoperative Neutrophil-To-Lymphocyte Ratio is a Novel Biomarker for Predicting Worse Clinical Outcomes in Non-Muscle Invasive Bladder Cancer Patients With a Previous History of Smoking. Ann Surg Oncol (2016) 23:1039–47. doi: 10.1245/s10434-016-5578-4 27660257

[18] Feng L Wang L. Effects of Alcohol on the Morphological and Structural Changes in Oral Mucosa. Pak J Med Sci (2013) 29:1046–9. doi: 10.12669/pjms.294.3696 PMC3817782 24353685

[19] Liu Y Chen H Sun Z Chen X. Molecular Mechanisms of Ethanol-Associated Oro-Esophageal Squamous Cell Carcinoma. Cancer Lett (2015) 361:164–73. doi: 10.1016/j.canlet.2015.03.006 PMC4765374 25766659

[20] Muhaxheri G Vucicevic Boras V Fucic A Plavec D Sekerija M Filipovic M. Multivariate Analysis of Preoperative and Postoperative Neutrophil-to-Lymphocyte Ratio as an Indicator of Head and Neck Squamous Cell Carcinoma Outcome. Int J Oral Maxillofac Surg (2018) 47:965–70. doi: 10.1016/j.ijom.2018.02.011 29559186

[21] Hashibe M Brennan P Chuang SC Boccia S Castellsague X Chen C. Interaction Between Tobacco and Alcohol Use and the Risk of Head and Neck Cancer: Pooled Analysis in the International Head and Neck Cancer Epidemiology Consortium. Cancer Epidemiol Biomarkers Prev (2009) 18:541–50. doi: 10.1158/1055-9965.EPI-08-0347 PMC3051410 19190158

[22] Kreimer AR Clifford GM Boyle P Franceschi S. Human Papillomavirus Types in Head and Neck Squamous Cell Carcinomas Worldwide: A Systematic Review. Cancer Epidemiol Biomarkers Prev (2005) 14:467–75. doi: 10.1158/1055-9965.EPI-04-0551 15734974

[23] Gillison ML Koch WM Capone RB Spafford M Westra WH Wu L. Evidence for a Causal Association Between Human Papillomavirus and a Subset of Head and Neck Cancers. J Natl Cancer Inst (2000) 92:709–20. doi: 10.1093/jnci/92.9.709 10793107

[24] Jelihovschi I Bidescu AC Tucaliuc SE Iancu LS. Detection Of Human Papilloma Virus In Head And Neck Squamous Cell Carcinomas: A Literature Review. Rev Med Chir Soc Med Nat Iasi (2015) 119:502–9. 26204659

[25] Dalla Torre D Burtscher D Sölder E Rasse M Puelacher W. The Correlation Between the Quality of Oral Hygiene and Oral HPV Infection in Adults: A Prospective Cross-Sectional Study. Clin Oral Investig (2019) 23:179–85. doi: 10.1007/s00784-018-2425-y 29574499

[26] Li C Zhao L Wang Q Ma S Sun J Ma C. Neutrophils Infiltration and its Correlation With Human Papillomavirus Status in the Oral Squamous Cell Carcinoma. Cancer Manag Res (2019) 11:5171–85. doi: 10.2147/CMAR.S202465 PMC6557188 31239772

[27] So YK Lee G Oh D Byeon S Park W Chung MK. Prognostic Role of Neutrophil-To-Lymphocyte Ratio in Patients With Human Papillomavirus-Positive Oropharyngeal Cancer. Otolaryngol Head Neck Surg (2018) 159:303–9. doi: 10.1177/0194599818764651 29557259

[28] Rosculet N Zhou XC Ha P Tang M Levine MA Neuner G. Neutrophil-To-Lymphocyte Ratio: Prognostic Indicator for Head and Neck Squamous Cell Carcinoma. Head Neck (2017) 39:662–7. doi: 10.1002/hed.24658 28075517

[29] Fanetti G Alterio D Marvaso G Gandini S Rojas DP Gobitti C. Prognostic Significance of Neutrophil-to-Lymphocyte Ratio in HPV Status Era for Oropharyngeal Cancer. Oral Dis (2020) 26:1384–92. doi: 10.1111/odi.13366 32315470

[30] Valdes M Villeda J Mithoowani H Pitre T Chasen M. Inflammatory Markers as Prognostic Factors of Recurrence in Advanced-Stage Squamous Cell Carcinoma of the Head and Neck. Curr Oncol (2020) 27:135–41. doi: 10.3747/co.27.5731 PMC7339834 32669922

[31] Shimakage M Horii K Tempaku A Kakudo K Shirasaka T Sasagawa T. Association of Epstein-Barr Virus With Oral Cancers. Hum Pathol (2002) 33:608–14. doi: 10.1053/hupa.2002.129786 12152159

[32] Horiuchi K Mishima K Ichijima K Sugimura M Ishida T Kirita T. Epstein-Barr Virus in the Proliferative Diseases of Squamous Epithelium in the Oral Cavity. Oral Surg Oral Med Oral Pathol Oral Radiol Endod (1995) 79:57–63. doi: 10.1016/S1079-2104(05)80075-7 7614163

[33] González-Moles MA Scully C Ruiz-Ávila I Plaza-Campillo JJ. The Cancer Stem Cell Hypothesis Applied to Oral Carcinoma. Oral Oncol (2013) 49:738–46. doi: 10.1016/j.oraloncology.2013.04.002 23642758

[34] González-Moles M Gutiérrez J Ruiz I Fernández JA Rodriguez M Aneiros J. Epstein-Barr Virus and Oral Squamous Cell Carcinoma in Patients Without HIV Infection: Viral Detection by Polymerase Chain Reaction. Microbios (1998) 96:23–31. 10347899

[35] Chua ML Tan SH Kusumawidjaja G Shwe MT Cheah SL Fong KW. Neutrophil-To-Lymphocyte Ratio as a Prognostic Marker in Locally Advanced Nasopharyngeal Carcinoma: A Pooled Analysis of Two Randomised Controlled Trials. Eur J Cancer (2016) 67:119–29. doi: 10.1016/j.ejca.2016.08.006 27640138

[36] Fan Y Zheng L Mao MH Huang MW Liu SM Zhang J. Survival Analysis of Oral Squamous Cell Carcinoma in a Subgroup of Young Patients. Asian Pac J Cancer Prev (2014) 15:8887–91. doi: 10.7314/APJCP.2014.15.20.8887 25374224

[37] Al-Amad SH Awad MA Nimri O. Oral Cancer in Young Jordanians: Potential Association With Frequency of Narghile Smoking. Oral Surg Oral Med Oral Pathol Oral Radiol (2014) 118:560–5. doi: 10.1016/j.oooo.2014.08.002 25442492

[38] Gawecki W Kostrzewska-Poczekaj M Gajecka M Milecki P Szyfter K Szyfter W. The Role of Genetic Factor in Etiopathogenesis of Squamous Cell Carcinoma of the Head and Neck in Young Adults. Eur Arch Otorhinolaryngol (2007) 264:1459–65. doi: 10.1007/s00405-007-0386-x 17653748

[39] Baker JL Bor B Agnello M Shi W He X. Ecology of the Oral Microbiome: Beyond Bacteria. Trends Microbiol (2017) 25:362–74. doi: 10.1016/j.tim.2016.12.012 PMC5687246 28089325

[40] Zhang WL Wang SS Wang HF Tang YJ Tang YL Liang XH. Who is Who in Oral Cancer? Exp Cell Res (2019) 384:111634. doi: 10.1016/j.yexcr.2019.111634 31541617

[41] Lafuente Ibáñez de Mendoza I Maritxalar Mendia X García de la Fuente AM Quindós Andrés G Aguirre Urizar JM. Role of Porphyromonas Gingivalis in Oral Squamous Cell Carcinoma Development: A Systematic Review. J Periodontal Res (2020) 55:13–22. doi: 10.1111/jre.12691 31529626

[42] Guerrero-Preston R Godoy-Vitorino F Jedlicka A Rodríguez-Hilario A González H Bondy J. 16s rRNA Amplicon Sequencing Identifies Microbiota Associated With Oral Cancer, Human Papilloma Virus Infection and Surgical Treatment. Oncotarget (2016) 7:51320–34. doi: 10.18632/oncotarget.9710 PMC5239478 27259999

[43] Groeger S Jarzina F Domann E Meyle J. Porphyromonas Gingivalis Activates Nfκb and MAPK Pathways in Human Oral Epithelial Cells. BMC Immunol (2017) 18:1. doi: 10.1186/s12865-016-0185-5 28056810 PMC5217430

[44] Zhao H Chu M Huang Z Yang X Ran S Hu B. Variations in Oral Microbiota Associated With Oral Cancer. Sci Rep (2017) 7:11773. doi: 10.1038/s41598-017-11779-9 28924229 PMC5603520

[45] Hoppe T Kraus D Novak N Probstmeier R Frentzen M Wenghoefer M. Oral Pathogens Change Proliferation Properties of Oral Tumor Cells by Affecting Gene Expression of Human Defensins. Tumour Biol (2016) 37:13789–98. doi: 10.1007/s13277-016-5281-x 27481514

[46] Guo ZC Jumatai S Jing SL Hu LL Jia XY Gong ZC. Bioinformatics and Immunohistochemistry Analyses of Expression Levels and Clinical Significance of CXCL2 and TANs in an Oral Squamous Cell Carcinoma Tumor Microenvironment of Prophyromonas Gingivalis Infection. Oncol Lett (2021) 21:189. doi: 10.3892/ol.2021.12450 33574928 PMC7816391

[47] Simard EP Torre LA Jemal A. International Trends in Head and Neck Cancer Incidence Rates: Differences by Country, Sex and Anatomic Site. Oral Oncol (2014) 50:387–403. doi: 10.1016/j.oraloncology.2014.01.016 24530208

[48] van der Waal I. Potentially Malignant Disorders of the Oral and Oropharyngeal Mucosa; Terminology, Classification and Present Concepts of Management. Oral Oncol (2009) 45:317–23. doi: 10.1016/j.oraloncology.2008.05.016 18674954

[49] Brunotto M Zarate AM Bono A Barra JL Berra S. Risk Genes in Head and Neck Cancer: A Systematic Review and Meta-Analysis of Last 5 Years. Oral Oncol (2014) 50:178–88. doi: 10.1016/j.oraloncology.2013.12.007 24370206

[50] González MV Pello MF López-Larrea C Suárez C Menéndez MJ Coto E. Loss of Heterozygosity and Mutation Analysis of the P16 (9p21) and P53 (17p13) Genes in Squamous Cell Carcinoma of the Head and Neck. Clin Cancer Res (1995) 1:1043–9. 9816078

[51] Valko M Leibfritz D Moncol J Cronin MT Mazur M Telser J. Free Radicals and Antioxidants in Normal Physiological Functions and Human Disease. Int J Biochem Cell Biol (2007) 39:44–84. doi: 10.1016/j.biocel.2006.07.001 16978905

[52] Chaitanya NC Muthukrishnan A Babu DBG Kumari CS Lakshmi MA Palat G. Role of Vitamin E and Vitamin A in Oral Mucositis Induced by Cancer Chemo/Radiotherapy- A Meta-Analysis. J Clin Diagn Res (2017) 11:Ze06–ze9. doi: 10.7860/JCDR/2017/26845.9905 PMC5483828 28658926

[53] Bouayed J Bohn T. Exogenous Antioxidants–Double-Edged Swords in Cellular Redox State: Health Beneficial Effects at Physiologic Doses Versus Deleterious Effects at High Doses. Oxid Med Cell Longev (2010) 3:228–37. doi: 10.4161/oxim.3.4.12858 PMC2952083 20972369

[54] Singh AK Pandey P Tewari M Pandey HP Gambhir IS Shukla HS. Free Radicals Hasten Head and Neck Cancer Risk: A Study of Total Oxidant, Total Antioxidant, DNA Damage, and Histological Grade. J Postgrad Med (2016) 62:96–101. doi: 10.4103/0022-3859.180555 27089108 PMC4944358

[55] Malik UU Siddiqui IA Hashim Z Zarina S. Measurement of Serum Paraoxonase Activity and MDA Concentrations in Patients Suffering With Oral Squamous Cell Carcinoma. Clin Chim Acta (2014) 430:38–42. doi: 10.1016/j.cca.2013.12.033 24389054

[56] Mirza AH Thomas G Ottensmeier CH King EV. Importance of the Immune System in Head and Neck Cancer. Head Neck (2019) 41:2789–800. doi: 10.1002/hed.25716 30821023

[57] Gonzalez H Hagerling C Werb Z. Roles of the Immune System in Cancer: From Tumor Initiation to Metastatic Progression. Genes Dev (2018) 32:1267–84. doi: 10.1101/gad.314617.118 PMC6169832 30275043

[58] Elmusrati A Wang J Wang CY. Tumor Microenvironment and Immune Evasion in Head and Neck Squamous Cell Carcinoma. Int J Oral Sci (2021) 13:24. doi: 10.1038/s41368-021-00131-7 34341329 PMC8329257

[59] Jahanban-Esfahlan R Seidi K Banimohamad-Shotorbani B Jahanban-Esfahlan A Yousefi B. Combination of Nanotechnology With Vascular Targeting Agents for Effective Cancer Therapy. J Cell Physiol (2018) 233:2982–92. doi: 10.1002/jcp.26051 28608554

[60] Jahanban-Esfahlan R Seidi K Zarghami N. Tumor Vascular Infarction: Prospects and Challenges. Int J Hematol (2017) 105:244–56. doi: 10.1007/s12185-016-2171-3 28044258

[61] Peltanova B Raudenska M Masarik M. Effect of Tumor Microenvironment on Pathogenesis of the Head and Neck Squamous Cell Carcinoma: A Systematic Review. Mol Cancer (2019) 18:63. doi: 10.1186/s12943-019-0983-5 30927923 PMC6441173

[62] Belli C Trapani D Viale G D’Amico P Duso BA Della Vigna P. Targeting the Microenvironment in Solid Tumors. Cancer Treat Rev (2018) 65:22–32. doi: 10.1016/j.ctrv.2018.02.004 29502037

[63] Baghban R Roshangar L Jahanban-Esfahlan R Seidi K Ebrahimi-Kalan A Jaymand M. Tumor Microenvironment Complexity and Therapeutic Implications at a Glance. Cell Commun Signal (2020) 18:59. doi: 10.1186/s12964-020-0530-4 32264958 PMC7140346

[64] Ostroumov D Fekete-Drimusz N Saborowski M Kühnel F Woller N. CD4 and CD8 T Lymphocyte Interplay in Controlling Tumor Growth. Cell Mol Life Sci (2018) 75:689–713. doi: 10.1007/s00018-017-2686-7 29032503 PMC5769828

[65] Emens LA. Breast Cancer Immunobiology Driving Immunotherapy: Vaccines and Immune Checkpoint Blockade. Expert Rev Anticancer Ther (2012) 12:1597–611. doi: 10.1586/era.12.147 PMC3587160 23253225

[66] Becht E Giraldo NA Dieu-Nosjean MC Sautes-Fridman C Fridman WH. Cancer Immune Contexture and Immunotherapy. Curr Opin Immunol (2016) 39:7–13. doi: 10.1016/j.coi.2015.11.009 26708937

[67] Yamaguchi T Sakaguchi S. Regulatory T Cells in Immune Surveillance and Treatment of Cancer. Semin Cancer Biol (2006) 16:115–23. doi: 10.1016/j.semcancer.2005.11.005 16376102

[68] Zagury D Gallo RC. Anti-Cytokine Ab Immune Therapy: Present Status and Perspectives. Drug Discov Today (2004) 9:72–81. doi: 10.1016/S1359-6446(03)02955-6 15012931

[69] Bergman PJ. Cancer Immunotherapies. Vet Clin North Am Small Anim Pract (2019) 49:881–902. doi: 10.1016/j.cvsm.2019.04.010 31186125

[70] Kusmartsev S Nagaraj S Gabrilovich DI. Tumor-Associated CD8+ T Cell Tolerance Induced by Bone Marrow-Derived Immature Myeloid Cells. J Immunol (2005) 175:4583–92. doi: 10.4049/jimmunol.175.7.4583 PMC1350970 16177103

[71] Park SJ Nakagawa T Kitamura H Atsumi T Kamon H Sawa S. IL-6 Regulates *In Vivo* Dendritic Cell Differentiation Through STAT3 Activation. J Immunol (2004) 173:3844–54. doi: 10.4049/jimmunol.173.6.3844 15356132

[72] Steinbrink K Wölfl M Jonuleit H Knop J Enk AH. Induction of Tolerance by IL-10-Treated Dendritic Cells. J Immunol (1997) 159:4772–80. 9366401

[73] Polak KL Chernosky NM Smigiel JM Tamagno I Jackson MW. Balancing STAT Activity as a Therapeutic Strategy. Cancers (Basel) (2019) 11:1716–45. doi: 10.3390/cancers11111716 PMC6895889 31684144

[74] Tang LY Heller M Meng Z Yu LR Tang Y Zhou M. Transforming Growth Factor-β (TGF-β) Directly Activates the JAK1-STAT3 Axis to Induce Hepatic Fibrosis in Coordination With the SMAD Pathway. J Biol Chem (2017) 292:4302–12. doi: 10.1074/jbc.M116.773085 PMC5354477 28154170

[75] Galon J Costes A Sanchez-Cabo F Kirilovsky A Mlecnik B Lagorce-Pagès C. Type, Density, and Location of Immune Cells Within Human Colorectal Tumors Predict Clinical Outcome. Science (2006) 313:1960–4. doi: 10.1126/science.1129139 17008531

[76] Becht E Giraldo NA Dieu-Nosjean MC Sautès-Fridman C Fridman WH. Cancer Immune Contexture and Immunotherapy. Curr Opin Immunol (2016) 39:7–13. doi: 10.1016/j.coi.2015.11.009 26708937

[77] Dieu-Nosjean MC Antoine M Danel C Heudes D Wislez M Poulot V. Long-Term Survival for Patients With non-Small-Cell Lung Cancer With Intratumoral Lymphoid Structures. J Clin Oncol (2008) 26:4410–7. doi: 10.1200/JCO.2007.15.0284 18802153

[78] Zhang QW Liu L Gong CY Shi HS Zeng YH Wang XZ. Prognostic Significance of Tumor-Associated Macrophages in Solid Tumor: A Meta-Analysis of the Literature. PLoS One (2012) 7:e50946. doi: 10.1371/journal.pone.0050946 23284651 PMC3532403

[79] Coffelt SB Wellenstein MD de Visser KE. Neutrophils in Cancer: Neutral No More. Nat Rev Cancer (2016) 16:431–46. doi: 10.1038/nrc.2016.52 27282249

[80] Colotta F Re F Polentarutti N Sozzani S Mantovani A. Modulation of Granulocyte Survival and Programmed Cell Death by Cytokines and Bacterial Products. Blood (1992) 80:2012–20. doi: 10.1182/blood.V80.8.2012.2012 1382715

[81] van Raam BJ Drewniak A Groenewold V van den Berg TK Kuijpers TW. Granulocyte Colony-Stimulating Factor Delays Neutrophil Apoptosis by Inhibition of Calpains Upstream of Caspase-3. Blood (2008) 112:2046–54. doi: 10.1182/blood-2008-04-149575 PMC2518906 18524991

[82] Mackey JBG Coffelt SB Carlin LM. Neutrophil Maturity in Cancer. Front Immunol (2019) 10.1912 doi: 10.3389/fimmu.2019.01912 31474989 PMC6702268

[83] Martin C Burdon PC Bridger G Gutierrez-Ramos JC Williams TJ Rankin SM. Chemokines Acting *via* CXCR2 and CXCR4 Control the Release of Neutrophils From the Bone Marrow and Their Return Following Senescence. Immunity (2003) 19:583–93. doi: 10.1016/S1074-7613(03)00263-2 14563322

[84] Mittmann LA Haring F Schaubächer JB Hennel R Smiljanov B Zuchtriegel G. Uncoupled Biological and Chronological Aging of Neutrophils in Cancer Promotes Tumor Progression. J Immunother Cancer (2021) 9:1–13. doi: 10.1136/jitc-2021-003495 PMC8655594 34876407

[85] Houghton AM Rzymkiewicz DM Ji H Gregory AD Egea EE Metz HE. Neutrophil Elastase-Mediated Degradation of IRS-1 Accelerates Lung Tumor Growth. Nat Med (2010) 16:219–23. doi: 10.1038/nm.2084 PMC2821801 20081861

[86] Yu Y Wang H Yan A Wang H Li X Liu J. Pretreatment Neutrophil to Lymphocyte Ratio in Determining the Prognosis of Head and Neck Cancer: A Meta-Analysis. BMC Cancer (2018) 18:383. doi: 10.1186/s12885-018-4230-z 29618336 PMC5885417

[87] Valero C Zanoni DK McGill MR Ganly I Morris LGT Quer M. Pretreatment Peripheral Blood Leukocytes are Independent Predictors of Survival in Oral Cavity Cancer. Cancer (2020) 126:994–1003. doi: 10.1002/cncr.32591 31809562 PMC7971350

[88] Orditura M Galizia G Diana A Saccone C Cobellis L Ventriglia J. Neutrophil to Lymphocyte Ratio (NLR) for Prediction of Distant Metastasis-Free Survival (DMFS) in Early Breast Cancer: A Propensity Score-Matched Analysis. ESMO Open (2016) 1:e000038. doi: 10.1136/esmoopen-2016-000038 27843594 PMC5070254

[89] Fang LP Xu XY Ji Y Huang PW. The Prognostic Value of Preoperative Neutrophil-To-Lymphocyte Ratio in Resected Patients With Pancreatic Adenocarcinoma. World J Surg (2018) 42:3736–45. doi: 10.1007/s00268-018-4686-7 30014292

[90] Terashima T Yamashita T Iida N Yamashita T Nakagawa H Arai K. Blood Neutrophil to Lymphocyte Ratio as a Predictor in Patients With Advanced Hepatocellular Carcinoma Treated With Hepatic Arterial Infusion Chemotherapy. Hepatol Res (2015) 45:949–59. doi: 10.1111/hepr.12436 25319848

[91] Cho H Hur HW Kim SW Kim SH Kim JH Kim YT. Pre-Treatment Neutrophil to Lymphocyte Ratio is Elevated in Epithelial Ovarian Cancer and Predicts Survival After Treatment. Cancer Immunol Immunother (2009) 58:15–23. doi: 10.1007/s00262-008-0516-3 18414853 PMC11029845

[92] Guthrie GJ Charles KA Roxburgh CS Horgan PG McMillan DC Clarke SJ. The Systemic Inflammation-Based Neutrophil-Lymphocyte Ratio: Experience in Patients With Cancer. Crit Rev Oncol Hematol (2013) 88:218–30. doi: 10.1016/j.critrevonc.2013.03.010 23602134

[93] Geng SK Fu SM Fu YP Zhang HW. Neutrophil to Lymphocyte Ratio is a Prognostic Factor for Disease Free Survival in Patients With Breast Cancer Underwent Curative Resection. Med (Baltimore) (2018) 97:e11898. doi: 10.1097/MD.0000000000011898 PMC6392639 30170382

[94] Cupp MA Cariolou M Tzoulaki I Aune D Evangelou E Berlanga-Taylor AJ. Neutrophil to Lymphocyte Ratio and Cancer Prognosis: An Umbrella Review of Systematic Reviews and Meta-Analyses of Observational Studies. BMC Med (2020) 18:360. doi: 10.1186/s12916-020-01817-1 33213430 PMC7678319

[95] Mishra V Giri R Hota S Senapati U Sahu SK. Neutrophil-To-Lymphocyte Ratio as a Prognostic Factor in Oral Squamous Cell Carcinoma - A Single-Institutional Experience From a Developing Country. J Oral Maxillofac Pathol (2021) 25:322–6. doi: 10.4103/0973-029X.325235 PMC8491359 34703128

[96] van Egmond M Bakema JE. Neutrophils as Effector Cells for Antibody-Based Immunotherapy of Cancer. Semin Cancer Biol (2013) 23:190–9. doi: 10.1016/j.semcancer.2012.12.002 23287459

[97] Zhu EF Gai SA Opel CF Kwan BH Surana R Mihm MC. Synergistic Innate and Adaptive Immune Response to Combination Immunotherapy With Anti-Tumor Antigen Antibodies and Extended Serum Half-Life IL-2. Cancer Cell (2015) 27:489–501. doi: 10.1016/j.ccell.2015.03.004 25873172 PMC4398916

[98] Zanoni DK Valero C McGill MR Montero PH Shah JP Wong RJ. Distant Metastasis in Oral Squamous Cell Carcinoma: Does the Neutrophil-to-Lymphocyte Ratio Act as a Surrogate of the Host Immune Status? Oral Oncol (2021) 124:105641. doi: 10.1016/j.oraloncology.2021.105641 34864297 PMC9377276

[99] Smaglo BG Aldeghaither D Weiner LM. Antibody Therapy. In: Ratcliffe MJH, editor. Encyclopedia of Immunobiology. Oxford: Academic Press (2016). p. 550–9.

[100] Mendelsohn J Powis G. Chapter 43 - From Bench to Bedside With Targeted Therapies. In: Mendelsohn J Howley PM Israel MA Gray JW Thompson CB, editors. The Molecular Basis of Cancer, 3rd ed. Philadelphia: W.B. Saunders (2008). p. 521–30.

[101] Stegmaier K Sellers WR. 4 - Targeted Approaches to Drug Development. In: Orkin SH Fisher DE Look AT Lux SE Ginsburg D Nathan DG, editors. Oncology of Infancy and Childhood. Philadelphia: W.B. Saunders (2009). p. 57–98.

[102] Xin-Yuan L Huang W-L Qian Q-J Zou W-G Zhang Z-L Chu L. 2 - Cancer Targeting Gene–Viro–Therapy and its Promising Future: A Trend in Both Cancer Gene Therapy and Cancer Virotherapy. In: Liu X-Y Pestka S Shi Y-F, editors. Recent Advances in Cancer Research and Therapy. Oxford: Elsevier (2012). p. 33–83.

[103] Demers M Krause DS Schatzberg D Martinod K Voorhees JR Fuchs TA. Cancers Predispose Neutrophils to Release Extracellular DNA Traps That Contribute to Cancer-Associated Thrombosis. Proc Natl Acad Sci U S A (2012) 109:13076–81. doi: 10.1073/pnas.1200419109 PMC3420209 22826226

[104] Paneesha S McManus A Arya R Scriven N Farren T Nokes T. Frequency, Demographics and Risk (According to Tumour Type or Site) of Cancer-Associated Thrombosis Among Patients Seen at Outpatient DVT Clinics. Thromb Haemost (2010) 103:338–43. doi: 10.1160/TH09-06-0397 20024496

[105] Monti M De Rosa V Iommelli F Carriero MV Terlizzi C Camerlingo R. Neutrophil Extracellular Traps as an Adhesion Substrate for Different Tumor Cells Expressing RGD-Binding Integrins. Int J Mol Sci (2018) 19:2350–60. doi: 10.3390/ijms19082350 PMC6121671 30096958

[106] Park J Wysocki RW Amoozgar Z Maiorino L Fein MR Jorns J. Cancer Cells Induce Metastasis-Supporting Neutrophil Extracellular DNA Traps. Sci Transl Med (2016) 8:361ra138. doi: 10.1126/scitranslmed.aag1711 PMC5550900 27798263

[107] Garley M Dziemiańczyk-Pakieła D Ratajczak-Wrona W Pryczynicz A Nowak K Łazarczyk B. NETs Biomarkers in Saliva and Serum OSCC Patients: One Hypothesis, Two Conclusions. Adv Med Sci (2021) 67:45–54. doi: 10.1016/j.advms.2021.12.004 34971930

[108] Garley M Jabłońska E Miltyk W Grubczak K Ratajczak-Wrona W Grudzińska M. Cancers Cells in Traps? The Pathways of NETs Formation in Response to OSCC in Humans-A Pilot Study. Cancer Control (2020) 27:1073274820960473. doi: 10.1177/1073274820960473 33073595 PMC7791464

[109] Masucci MT Minopoli M Del Vecchio S Carriero MV. The Emerging Role of Neutrophil Extracellular Traps (NETs) in Tumor Progression and Metastasis. Front Immunol (2020) 11.1749 doi: 10.3389/fimmu.2020.01749 33042107 PMC7524869

[110] Chen N He D Cui J. A Neutrophil Extracellular Traps Signature Predicts the Clinical Outcomes and Immunotherapy Response in Head and Neck Squamous Cell Carcinoma. Front Mol Biosci (2022) 9:833771. doi: 10.3389/fmolb.2022.833771 35252353 PMC8894649

[111] Park JE Lee DH Lee JA Park SG Kim NS Park BC. Annexin A3 is a Potential Angiogenic Mediator. Biochem Biophys Res Commun (2005) 337:1283–7. doi: 10.1016/j.bbrc.2005.10.004 16236264

[112] Okubo K Kamiya M Urano Y Nishi H Herter JM Mayadas T. Lactoferrin Suppresses Neutrophil Extracellular Traps Release in Inflammation. EBioMedicine (2016) 10:204–15. doi: 10.1016/j.ebiom.2016.07.012 PMC5006695 27453322

[113] Trellakis S Farjah H Bruderek K Dumitru CA Hoffmann TK Lang S. Peripheral Blood Neutrophil Granulocytes From Patients With Head and Neck Squamous Cell Carcinoma Functionally Differ From Their Counterparts in Healthy Donors. Int J Immunopathol Pharmacol (2011) 24:683–93. doi: 10.1177/039463201102400314 21978700

[114] Jabłońska E Garley M Jabłoński J. The Expressions of Intrinsic and Extrinsic Apoptotic Pathway Proteins in Neutrophils of Oral Cavity Cancer Patients: A Preliminary Study. Arch Immunol Ther Exp (Warsz) (2009) 57:229–34. doi: 10.1007/s00005-009-0023-z 19479204

[115] Piazza C Incandela F Giannini L. Unknown Primary of the Head and Neck: A New Entry in the TNM Staging System With Old Dilemmas for Everyday Practice. Curr Opin Otolaryngol Head Neck Surg (2019) 27:73–9. doi: 10.1097/MOO.0000000000000528 30694915

[116] Clausen F Behrens HM Krüger S Röcken C. Sexual Dimorphism in Gastric Cancer: Tumor-Associated Neutrophils Predict Patient Outcome Only for Women. J Cancer Res Clin Oncol (2020) 146:53–66. doi: 10.1007/s00432-019-03082-z 31741042 PMC6942031

[117] Quaas A Pamuk A Klein S Quantius J Rehkaemper J Barutcu AG. Sex-Specific Prognostic Effect of CD66b-Positive Tumor-Infiltrating Neutrophils (TANs) in Gastric and Esophageal Adenocarcinoma. Gastric Cancer (2021) 24:1213–26. doi: 10.1007/s10120-021-01197-2 PMC8502159 34009535

[118] Kargl J Zhu X Zhang H Yang GHY Friesen TJ Shipley M. Neutrophil Content Predicts Lymphocyte Depletion and Anti-PD1 Treatment Failure in NSCLC. JCI Insight (2019) 4:1–17. doi: 10.1172/jci.insight.130850 PMC6975266 31852845

[119] Nebot AY L Aoufouchi S De Forceville L Danjou M Scoazec J Vuagnat P. Neutrophils are Associated With Resistance to Anti-PD-1 Monotherapy in Mismatch Repair-Deficient Tumors. Ann Oncol (2021) 32(suppl_5):S1227–36. doi: 10.1016/annonc/annonc681

[120] Treffers LW Hiemstra IH Kuijpers TW van den Berg TK Matlung HL. Neutrophils in Cancer. Immunol Rev (2016) 273:312–28. doi: 10.1111/imr.12444 27558343

[121] Fridlender ZG Sun J Kim S Kapoor V Cheng G Ling L. Polarization of Tumor-Associated Neutrophil Phenotype by TGF-Beta: “N1” versus “N2” TAN Cancer Cell (2009) 16:183–94. doi: 10.1016/j.ccr.2009.06.017 PMC2754404 19732719

[122] Saraiva DP Correia BF Salvador R de Sousa N Jacinto A Braga S. Circulating Low Density Neutrophils of Breast Cancer Patients are Associated With Their Worse Prognosis Due to the Impairment of T Cell Responses. Oncotarget (2021) 12:2388–403. doi: 10.18632/oncotarget.28135 PMC8629401 34853660

[123] Lonardi S Missale F Calza S Bugatti M Vescovi R Debora B. Tumor-Associated Neutrophils (TANs) in Human Carcinoma-Draining Lymph Nodes: A Novel TAN Compartment. Clin Transl Immunol (2021) 10:e1252. doi: 10.1002/cti2.1252 PMC7886597 33643653

[124] Takakura K Ito Z Suka M Kanai T Matsumoto Y Odahara S. Comprehensive Assessment of the Prognosis of Pancreatic Cancer: Peripheral Blood Neutrophil-Lymphocyte Ratio and Immunohistochemical Analyses of the Tumour Site. Scand J Gastroenterol (2016) 51:610–7. doi: 10.3109/00365521.2015.1121515 26679084

[125] Ohms M Möller S Laskay T. An Attempt to Polarize Human Neutrophils Toward N1 and N2 Phenotypes *In Vitro* . Front Immunol (2020) 11:532. doi: 10.3389/fimmu.2020.00532 32411122 PMC7198726

[126] Brandau S Trellakis S Bruderek K Schmaltz D Steller G Elian M. Myeloid-Derived Suppressor Cells in the Peripheral Blood of Cancer Patients Contain a Subset of Immature Neutrophils With Impaired Migratory Properties. J Leukoc Biol (2011) 89:311–7. doi: 10.1189/jlb.0310162 21106641

[127] Masucci MT Minopoli M Carriero MV. Tumor Associated Neutrophils. Their Role in Tumorigenesis, Metastasis, Prognosis and Therapy. Front Oncol (2019) 9:1146–. doi: 10.3389/fonc.2019.01146 PMC6874146 31799175

[128] Zhou SL Zhou ZJ Hu ZQ Huang XW Wang Z Chen EB. Tumor-Associated Neutrophils Recruit Macrophages and T-Regulatory Cells to Promote Progression of Hepatocellular Carcinoma and Resistance to Sorafenib. Gastroenterology (2016) 150:1646–58.e17. doi: 10.1053/j.gastro.2016.02.040 26924089

[129] Eruslanov EB Bhojnagarwala PS Quatromoni JG Stephen TL Ranganathan A Deshpande C. Tumor-Associated Neutrophils Stimulate T Cell Responses in Early-Stage Human Lung Cancer. J Clin Invest (2014) 124:5466–80. doi: 10.1172/JCI77053 PMC4348966 25384214

[130] Wu P Wu D Ni C Ye J Chen W Hu G. γδt17 Cells Promote the Accumulation and Expansion of Myeloid-Derived Suppressor Cells in Human Colorectal Cancer. Immunity (2014) 40:785–800. doi: 10.1016/j.immuni.2014.03.013 24816404 PMC4716654

[131] Damgaard C Kantarci A Holmstrup P Hasturk H Nielsen CH Van Dyke TE. Porphyromonas Gingivalis-Induced Production of Reactive Oxygen Species, Tumor Necrosis Factor-α, Interleukin-6, CXCL8 and CCL2 by Neutrophils From Localized Aggressive Periodontitis and Healthy Donors: Modulating Actions of Red Blood Cells and Resolvin E1. J Periodontal Res (2017) 52:246–54. doi: 10.1111/jre.12388 PMC5097708 27146665

[132] Wen L Mu W Lu H Wang X Fang J Jia Y. Porphyromonas Gingivalis Promotes Oral Squamous Cell Carcinoma Progression in an Immune Microenvironment. J Dent Res (2020) 99:666–75. doi: 10.1177/0022034520909312 32298192

[133] Flavell RA Sanjabi S Wrzesinski SH Licona-Limón P. The Polarization of Immune Cells in the Tumour Environment by TGFbeta. Nat Rev Immunol (2010) 10:554–67. doi: 10.1038/nri2808 PMC3885992 20616810

[134] Masucci MT Minopoli M Carriero MV. Tumor Associated Neutrophils. Their Role in Tumorigenesis, Metastasis, Prognosis and Therapy. Front Oncol (2019) 9. doi: 10.3389/fonc.2019.01146 PMC6874146 31799175

[135] Mishalian I Bayuh R Levy L Zolotarov L Michaeli J Fridlender ZG. Tumor-Associated Neutrophils (TAN) Develop Pro-Tumorigenic Properties During Tumor Progression. Cancer Immunol Immunother (2013) 62:1745–56. doi: 10.1007/s00262-013-1476-9 PMC11028422 24092389

[136] Bergers G Brekken R McMahon G Vu TH Itoh T Tamaki K. Matrix Metalloproteinase-9 Triggers the Angiogenic Switch During Carcinogenesis. Nat Cell Biol (2000) 2:737–44. doi: 10.1038/35036374 PMC2852586 11025665

[137] Hamada T Duarte S Tsuchihashi S Busuttil RW Coito AJ. Inducible Nitric Oxide Synthase Deficiency Impairs Matrix Metalloproteinase-9 Activity and Disrupts Leukocyte Migration in Hepatic Ischemia/Reperfusion Injury. Am J Pathol (2009) 174:2265–77. doi: 10.2353/ajpath.2009.080872 PMC2684191 19443702

[138] Ardi VC Kupriyanova TA Deryugina EI Quigley JP. Human Neutrophils Uniquely Release TIMP-Free MMP-9 to Provide a Potent Catalytic Stimulator of Angiogenesis. Proc Natl Acad Sci U S A (2007) 104:20262–7. doi: 10.1073/pnas.0706438104 PMC2154419 18077379

[139] Musrati AA Tervahartiala T Gürsoy M Könönen E Fteita D Sorsa T. Human Neutrophil Peptide-1 Affects Matrix Metalloproteinase-2, -8 and -9 Secretions of Oral Squamous Cell Carcinoma Cell Lines *In Vitro* . Arch Oral Biol (2016) 66:1–7. doi: 10.1016/j.archoralbio.2016.02.003 26872095

[140] Reiter RJ. Mechanisms of Cancer Inhibition by Melatonin. J Pineal Res (2004) 37:213–4. doi: 10.1111/j.1600-079X.2004.00165.x 15357667

[141] Lu H Wu B Ma G Zheng D Song R Huang E. Melatonin Represses Oral Squamous Cell Carcinoma Metastasis by Inhibiting Tumor-Associated Neutrophils. Am J Transl Res (2017) 9:5361–74. PMC5752887 29312489

[142] Pham CT. Neutrophil Serine Proteases: Specific Regulators of Inflammation. Nat Rev Immunol (2006) 6:541–50. doi: 10.1038/nri1841 16799473

[143] Gaida MM Steffen TG Günther F Tschaharganeh DF Felix K Bergmann F. Polymorphonuclear Neutrophils Promote Dyshesion of Tumor Cells and Elastase-Mediated Degradation of E-Cadherin in Pancreatic Tumors. Eur J Immunol (2012) 42:3369–80. doi: 10.1002/eji.201242628 23001948

[144] Deryugina E Carré A Ardi V Muramatsu T Schmidt J Pham C. Neutrophil Elastase Facilitates Tumor Cell Intravasation and Early Metastatic Events. iScience (2020) 23:101799. doi: 10.1016/j.isci.2020.101799 33299970 PMC7702017

[145] Wen J Nikitakis NG Chaisuparat R Greenwell-Wild T Gliozzi M Jin W. Secretory Leukocyte Protease Inhibitor (SLPI) Expression and Tumor Invasion in Oral Squamous Cell Carcinoma. Am J Pathol (2011) 178:2866–78. doi: 10.1016/j.ajpath.2011.02.017 PMC3124294 21641406

[146] Andzinski L Kasnitz N Stahnke S Wu CF Gereke M von Köckritz-Blickwede M. Type I IFNs Induce Anti-Tumor Polarization of Tumor Associated Neutrophils in Mice and Human. Int J Cancer (2016) 138:1982–93. doi: 10.1002/ijc.29945 26619320

[147] Nauseef WM. How Human Neutrophils Kill and Degrade Microbes: An Integrated View. Immunol Rev (2007) 219:88–102. doi: 10.1111/j.1600-065X.2007.00550.x 17850484

[148] Sagiv JY Michaeli J Assi S Mishalian I Kisos H Levy L. Phenotypic Diversity and Plasticity in Circulating Neutrophil Subpopulations in Cancer. Cell Rep (2015) 10:562–73. doi: 10.1016/j.celrep.2014.12.039 25620698

[149] He JR Shen GP Ren ZF Qin H Cui C Zhang Y. Pretreatment Levels of Peripheral Neutrophils and Lymphocytes as Independent Prognostic Factors in Patients With Nasopharyngeal Carcinoma. Head Neck (2012) 34:1769–76. doi: 10.1002/hed.22008 22318781

[150] An X Ding PR Wang FH Jiang WQ Li YH. Elevated Neutrophil to Lymphocyte Ratio Predicts Poor Prognosis in Nasopharyngeal Carcinoma. Tumour Biol (2011) 32:317–24. doi: 10.1007/s13277-010-0124-7 21052888

[151] Tachinami H Tomihara K Ikeda A Sekido K Sakurai K Imaue S. [Neutrophil-To-Lymphocyte Ratio(NLR)as a Predictive Indicator of the Response to Nivolumab in Patients With Oral Squamous Cell Carcinoma]. Gan To Kagaku Ryoho (2021) 48:1485–90. 34911916

[152] Huang CH Chen PR Lue KH Hsieh TC Chou YF. Evaluation of Sarcopenia, Frailty, and Inflammation on Adverse Events and Survival Outcomes in Patients With Oral Cavity Squamous Cell Carcinoma Under Adjuvant Chemoradiotherapy. J Pers Med (2021) 11:936–47. doi: 10.3390/jpm11090936 PMC8464994 34575713

[153] Ding M Song Y Jing J Tian M Ding L Li Q. The Ratio of Preoperative Serum Biomarkers Predicts Prognosis in Patients With Oral Squamous Cell Carcinoma. Front Oncol (2021) 11:719513. doi: 10.3389/fonc.2021.719513 34552873 PMC8452155

[154] Sionov RV Fainsod-Levi T Zelter T Polyansky L Pham CT Granot Z. Neutrophil Cathepsin G and Tumor Cell RAGE Facilitate Neutrophil Anti-Tumor Cytotoxicity. Oncoimmunology (2019) 8:e1624129. doi: 10.1080/2162402X.2019.1624129 31428521 PMC6685517

[155] Sionov RV Assi S Gershkovitz M Sagiv JY Polyansky L Mishalian I. Isolation and Characterization of Neutrophils With Anti-Tumor Properties. J Vis Exp (2015) (100):e52933. doi: 10.3791/52933 26132785 PMC4544930

[156] Sasahira T Kirita T Bhawal UK Yamamoto K Ohmori H Fujii K. Receptor for Advanced Glycation End Products (RAGE) is Important in the Prediction of Recurrence in Human Oral Squamous Cell Carcinoma. Histopathology (2007) 51:166–72. doi: 10.1111/j.1365-2559.2007.02739.x 17593216

[157] Landesberg R Woo V Huang L Cozin M Lu Y Bailey C. The Expression of the Receptor for Glycation Endproducts (RAGE) in Oral Squamous Cell Carcinomas. Oral Surg Oral Med Oral Pathol Oral Radiol Endod (2008) 105:617–24. doi: 10.1016/j.tripleo.2007.08.006 18206396

[158] Bhawal UK Ozaki Y Nishimura M Sugiyama M Sasahira T Nomura Y. Association of Expression of Receptor for Advanced Glycation End Products and Invasive Activity of Oral Squamous Cell Carcinoma. Oncology (2005) 69:246–55. doi: 10.1159/000087910 16127291

[159] Granot Z Henke E Comen EA King TA Norton L Benezra R. Tumor Entrained Neutrophils Inhibit Seeding in the Premetastatic Lung. Cancer Cell (2011) 20:300–14. doi: 10.1016/j.ccr.2011.08.012 PMC3172582 21907922

[160] Gershkovitz M Caspi Y Fainsod-Levi T Katz B Michaeli J Khawaled S. TRPM2 Mediates Neutrophil Killing of Disseminated Tumor Cells. Cancer Res (2018) 78:2680–90. doi: 10.1158/0008-5472.CAN-17-3614 29490946

[161] Hara Y Wakamori M Ishii M Maeno E Nishida M Yoshida T. LTRPC2 Ca2+-Permeable Channel Activated by Changes in Redox Status Confers Susceptibility to Cell Death. Mol Cell (2002) 9:163–73. doi: 10.1016/S1097-2765(01)00438-5 11804595

[162] Gershkovitz M Fainsod-Levi T Khawaled S Shaul ME Sionov RV Cohen-Daniel L. Microenvironmental Cues Determine Tumor Cell Susceptibility to Neutrophil Cytotoxicity. Cancer Res (2018) 78:5050–9. doi: 10.1158/0008-5472.CAN-18-0540 29967257

[163] Zhao LY Xu WL Xu ZQ Qi C Li Y Cheng J. The Overexpressed Functional Transient Receptor Potential Channel TRPM2 in Oral Squamous Cell Carcinoma. Sci Rep (2016) 6:38471. doi: 10.1038/srep38471 28008929 PMC5180100

[164] Catena R Bhattacharya N El Rayes T Wang S Choi H Gao D. Bone Marrow-Derived Gr1+ Cells can Generate a Metastasis-Resistant Microenvironment *via* Induced Secretion of Thrombospondin-1. Cancer Discov (2013) 3:578–89. doi: 10.1158/2159-8290.CD-12-0476 PMC3672408 23633432

[165] Finisguerra V Di Conza G Di Matteo M Serneels J Costa S Thompson AA. MET is Required for the Recruitment of Anti-Tumoural Neutrophils. Nature (2015) 522:349–53. doi: 10.1038/nature14407 PMC4594765 25985180

[166] Luraghi P Schelter F Krüger A Boccaccio C. The MET Oncogene as a Therapeutical Target in Cancer Invasive Growth. Front Pharmacol (2012) 3:164. doi: 10.3389/fphar.2012.00164 22973229 PMC3438853

[167] Hara S Nakashiro K Klosek SK Ishikawa T Shintani S Hamakawa H. Hypoxia Enhances C-Met/HGF Receptor Expression and Signaling by Activating HIF-1alpha in Human Salivary Gland Cancer Cells. Oral Oncol (2006) 42:593–8. doi: 10.1016/j.oraloncology.2005.10.016 16469527

[168] Bergenfelz C Leandersson K. The Generation and Identity of Human Myeloid-Derived Suppressor Cells. Front Oncol (2020) 10:109. doi: 10.3389/fonc.2020.00109 32117758 PMC7025543

[169] Gabrilovich DI. Myeloid-Derived Suppressor Cells. Cancer Immunol Res (2017) 5:3–8. doi: 10.1158/2326-6066.CIR-16-0297 28052991 PMC5426480

[170] Chu M Su YX Wang L Zhang TH Liang YJ Liang LZ. Myeloid-Derived Suppressor Cells Contribute to Oral Cancer Progression in 4NQO-Treated Mice. Oral Dis (2012) 18:67–73. doi: 10.1111/j.1601-0825.2011.01846.x 21883708

[171] Zhou J Nefedova Y Lei A Gabrilovich D. Neutrophils and PMN-MDSC: Their Biological Role and Interaction With Stromal Cells. Semin Immunol (2018) 35:19–28. doi: 10.1016/j.smim.2017.12.004 29254756 PMC5866202

[172] Lang S Bruderek K Kaspar C Höing B Kanaan O Dominas N. Clinical Relevance and Suppressive Capacity of Human Myeloid-Derived Suppressor Cell Subsets. Clin Cancer Res (2018) 24:4834–44. doi: 10.1158/1078-0432.CCR-17-3726 29914893

[173] Abeles RD McPhail MJ Sowter D Antoniades CG Vergis N Vijay GK. CD14, CD16 and HLA-DR Reliably Identifies Human Monocytes and Their Subsets in the Context of Pathologically Reduced HLA-DR Expression by CD14(hi)/CD16(neg) Monocytes: Expansion of CD14(hi)/CD16(pos) and Contraction of CD14(lo)/CD16(pos) Monocytes in Acute Liver Failure. Cytometry A (2012) 81:823–34. doi: 10.1002/cyto.a.22104 22837127

[174] Damuzzo V Pinton L Desantis G Solito S Marigo I Bronte V. Complexity and Challenges in Defining Myeloid-Derived Suppressor Cells. Cytometry B Clin Cytom (2015) 88:77–91. doi: 10.1002/cytob.21206 25504825 PMC4405078

[175] Dumitru CA Moses K Trellakis S Lang S Brandau S. Neutrophils and Granulocytic Myeloid-Derived Suppressor Cells: Immunophenotyping, Cell Biology and Clinical Relevance in Human Oncology. Cancer Immunol Immunother (2012) 61:1155–67. doi: 10.1007/s00262-012-1294-5 PMC11028504 22692756

[176] Gustafson MP Lin Y Maas ML Van Keulen VP Johnston PB Peikert T. A Method for Identification and Analysis of non-Overlapping Myeloid Immunophenotypes in Humans. PLoS One (2015) 10:e0121546. doi: 10.1371/journal.pone.0121546 25799053 PMC4370675

[177] Condamine T Dominguez GA Youn JI Kossenkov AV Mony S Alicea-Torres K. Lectin-Type Oxidized LDL Receptor-1 Distinguishes Population of Human Polymorphonuclear Myeloid-Derived Suppressor Cells in Cancer Patients. Sci Immunol (2016) 1:1–32. doi: 10.1126/sciimmunol.aaf8943 PMC5391495 28417112

[178] Sekido K Tomihara K Tachinami H Heshiki W Sakurai K Moniruzzaman R. Alterations in Composition of Immune Cells and Impairment of Anti-Tumor Immune Response in Aged Oral Cancer-Bearing Mice. Oral Oncol (2019) 99:104462. doi: 10.1016/j.oraloncology.2019.104462 31683168

[179] Lechner MG Liebertz DJ Epstein AL. Characterization of Cytokine-Induced Myeloid-Derived Suppressor Cells From Normal Human Peripheral Blood Mononuclear Cells. J Immunol (2010) 185:2273–84. doi: 10.4049/jimmunol.1000901 PMC2923483 20644162

[180] Rodriguez PC Ernstoff MS Hernandez C Atkins M Zabaleta J Sierra R. Arginase I-Producing Myeloid-Derived Suppressor Cells in Renal Cell Carcinoma are a Subpopulation of Activated Granulocytes. Cancer Res (2009) 69:1553–60. doi: 10.1158/0008-5472.CAN-08-1921 PMC2900845 19201693

[181] Rotondo R Barisione G Mastracci L Grossi F Orengo AM Costa R. IL-8 Induces Exocytosis of Arginase 1 by Neutrophil Polymorphonuclears in Nonsmall Cell Lung Cancer. Int J Cancer (2009) 125:887–93. doi: 10.1002/ijc.24448 19431148

[182] Michaeli J Shaul ME Mishalian I Hovav AH Levy L Zolotriov L. Tumor-Associated Neutrophils Induce Apoptosis of non-Activated CD8 T-Cells in a Tnfα and NO-Dependent Mechanism, Promoting a Tumor-Supportive Environment. Oncoimmunology (2017) 6:e1356965. doi: 10.1080/2162402X.2017.1356965 29147615 PMC5674962

[183] Pak AS Wright MA Matthews JP Collins SL Petruzzelli GJ Young MR. Mechanisms of Immune Suppression in Patients With Head and Neck Cancer: Presence of CD34(+) Cells Which Suppress Immune Functions Within Cancers That Secrete Granulocyte-Macrophage Colony-Stimulating Factor. Clin Cancer Res (1995) 1:95–103. 9815891

[184] Young MR Wright MA Lozano Y Prechel MM Benefield J Leonetti JP. Increased Recurrence and Metastasis in Patients Whose Primary Head and Neck Squamous Cell Carcinomas Secreted Granulocyte-Macrophage Colony-Stimulating Factor and Contained CD34+ Natural Suppressor Cells. Int J Cancer (1997) 74:69–74. doi: 10.1002/(SICI)1097-0215(19970220)74:1<69::AID-IJC12>3.0.CO;2-D 9036872

[185] Garrity T Pandit R Wright MA Benefield J Keni S Young MR. Increased Presence of CD34+ Cells in the Peripheral Blood of Head and Neck Cancer Patients and Their Differentiation Into Dendritic Cells. Int J Cancer (1997) 73:663–9. doi: 10.1002/(SICI)1097-0215(19971127)73:5<663::AID-IJC9>3.0.CO;2-V 9398043

[186] Du R Lu KV Petritsch C Liu P Ganss R Passegué E. HIF1alpha Induces the Recruitment of Bone Marrow-Derived Vascular Modulatory Cells to Regulate Tumor Angiogenesis and Invasion. Cancer Cell (2008) 13:206–20. doi: 10.1016/j.ccr.2008.01.034 PMC2643426 18328425

[187] Najafi M Farhood B Mortezaee K. Extracellular Matrix (ECM) Stiffness and Degradation as Cancer Drivers. J Cell Biochem (2019) 120:2782–90. doi: 10.1002/jcb.27681 30321449

[188] Rahat MA Coffelt SB Granot Z Muthana M Amedei A. Macrophages and Neutrophils: Regulation of the Inflammatory Microenvironment in Autoimmunity and Cancer. Mediators Inflammation (2016) 2016:5894347. doi: 10.1155/2016/5894347 PMC5048028 27725789

[189] Wu L Zhang XH. Tumor-Associated Neutrophils and Macrophages-Heterogenous But Not Chaotic. Front Immunol (2020) 11:553967. doi: 10.3389/fimmu.2020.553967 33343560 PMC7738476

[190] Tu S Lin X Qiu J Zhou J Wang H Hu S. Crosstalk Between Tumor-Associated Microglia/Macrophages and CD8-Positive T Cells Plays a Key Role in Glioblastoma. Front Immunol (2021) 12:650105. doi: 10.3389/fimmu.2021.650105 34394072 PMC8358794

[191] Kumar V Sharma A. Neutrophils: Cinderella of Innate Immune System. Int Immunopharmacol (2010) 10:1325–34. doi: 10.1016/j.intimp.2010.08.012 20828640

[192] Bennouna S Bliss SK Curiel TJ Denkers EY. Cross-Talk in the Innate Immune System: Neutrophils Instruct Recruitment and Activation of Dendritic Cells During Microbial Infection. J Immunol (2003) 171:6052–8. doi: 10.4049/jimmunol.171.11.6052 14634118

[193] Kasama T Strieter RM Standiford TJ Burdick MD Kunkel SL. Expression and Regulation of Human Neutrophil-Derived Macrophage Inflammatory Protein 1 Alpha. J Exp Med (1993) 178:63–72. doi: 10.1084/jem.178.1.63 8315395 PMC2191098

[194] Kasama T Strieter RM Lukacs NW Burdick MD Kunkel SL. Regulation of Neutrophil-Derived Chemokine Expression by IL-10. J Immunol (1994) 152:3559–69. 8144935

[195] Shepherd VL Hoidal JR. Clearance of Neutrophil-Derived Myeloperoxidase by the Macrophage Mannose Receptor. Am J Respir Cell Mol Biol (1990) 2:335–40. doi: 10.1165/ajrcmb/2.4.335 2157473

[196] Lefkowitz DL Lefkowitz SS. Macrophage-Neutrophil Interaction: A Paradigm for Chronic Inflammation Revisited. Immunol Cell Biol (2001) 79:502–6. doi: 10.1046/j.1440-1711.2001.01020.x 11564158

[197] Takano T Azuma N Satoh M Toda A Hashida Y Satoh R. Neutrophil Survival Factors (TNF-Alpha, GM-CSF, and G-CSF) Produced by Macrophages in Cats Infected With Feline Infectious Peritonitis Virus Contribute to the Pathogenesis of Granulomatous Lesions. Arch Virol (2009) 154:775–81. doi: 10.1007/s00705-009-0371-3 PMC7086964 19343474

[198] Droeser RA Hirt C Eppenberger-Castori S Zlobec I Viehl CT Frey DM. High Myeloperoxidase Positive Cell Infiltration in Colorectal Cancer is an Independent Favorable Prognostic Factor. PLoS One (2013) 8:e64814. doi: 10.1371/journal.pone.0064814 23734221 PMC3667167

[199] Rymaszewski AL Tate E Yimbesalu JP Gelman AE Jarzembowski JA Zhang H. The Role of Neutrophil Myeloperoxidase in Models of Lung Tumor Development. Cancers (Basel) (2014) 6:1111–27. doi: 10.3390/cancers6021111 PMC4074819 24821130

[200] Kondo Y Suzuki S Takahara T Ono S Goto M Miyabe S. Improving Function of Cytotoxic T-Lymphocytes by Transforming Growth Factor-β Inhibitor in Oral Squamous Cell Carcinoma. Cancer Sci (2021) 112:4037–49. doi: 10.1111/cas.15081 PMC8486191 34309966

[201] Kim J Bae JS. Tumor-Associated Macrophages and Neutrophils in Tumor Microenvironment. Mediators Inflammation (2016) 2016:6058147. doi: 10.1155/2016/6058147 PMC4757693 26966341

[202] Abraham D Zins K Sioud M Lucas T Schäfer R Stanley ER. Stromal Cell-Derived CSF-1 Blockade Prolongs Xenograft Survival of CSF-1-Negative Neuroblastoma. Int J Cancer (2010) 126:1339–52. doi: 10.1002/ijc.24859 PMC3222589 19711348

[203] Quail DF Joyce JA. Microenvironmental Regulation of Tumor Progression and Metastasis. Nat Med (2013) 19:1423–37. doi: 10.1038/nm.3394 PMC3954707 24202395

[204] Nakatsumi H Matsumoto M Nakayama KI. Noncanonical Pathway for Regulation of CCL2 Expression by an Mtorc1-FOXK1 Axis Promotes Recruitment of Tumor-Associated Macrophages. Cell Rep (2017) 21:2471–86. doi: 10.1016/j.celrep.2017.11.014 29186685

[205] Franklin RA Liao W Sarkar A Kim MV Bivona MR Liu K. The Cellular and Molecular Origin of Tumor-Associated Macrophages. Science (2014) 344:921–5. doi: 10.1126/science.1252510 PMC4204732 24812208

[206] Tymoszuk P Evens H Marzola V Wachowicz K Wasmer MH Datta S. *In Situ* Proliferation Contributes to Accumulation of Tumor-Associated Macrophages in Spontaneous Mammary Tumors. Eur J Immunol (2014) 44:2247–62. doi: 10.1002/eji.201344304 24796276

[207] Van Overmeire E Stijlemans B Heymann F Keirsse J Morias Y Elkrim Y. M-CSF and GM-CSF Receptor Signaling Differentially Regulate Monocyte Maturation and Macrophage Polarization in the Tumor Microenvironment. Cancer Res (2016) 76:35–42. doi: 10.1158/0008-5472.CAN-15-0869 26573801

[208] Braza MS Conde P Garcia M Cortegano I Brahmachary M Pothula V. Neutrophil Derived CSF1 Induces Macrophage Polarization and Promotes Transplantation Tolerance. Am J Transplant (2018) 18:1247–55. doi: 10.1111/ajt.14645 PMC5910259 29314558

[209] DeNardo DG Ruffell B. Macrophages as Regulators of Tumour Immunity and Immunotherapy. Nat Rev Immunol (2019) 19:369–82. doi: 10.1038/s41577-019-0127-6 PMC7339861 30718830

[210] Sica A Mantovani A. Macrophage Plasticity and Polarization: *In Vivo* Veritas. J Clin Invest (2012) 122:787–95. doi: 10.1172/JCI59643 PMC3287223 22378047

[211] Mitrofanova I Zavyalova M Telegina N Buldakov M Riabov V Cherdyntseva N. Tumor-Associated Macrophages in Human Breast Cancer Parenchyma Negatively Correlate With Lymphatic Metastasis After Neoadjuvant Chemotherapy. Immunobiology (2017) 222:101–9. doi: 10.1016/j.imbio.2016.08.001 27510849

[212] Allavena P Mantovani A. Immunology in the Clinic Review Series; Focus on Cancer: Tumour-Associated Macrophages: Undisputed Stars of the Inflammatory Tumour Microenvironment. Clin Exp Immunol (2012) 167:195–205. doi: 10.1111/j.1365-2249.2011.04515.x 22235995 PMC3278685

[213] Pinto ML Rios E Durães C Ribeiro R Machado JC Mantovani A. The Two Faces of Tumor-Associated Macrophages and Their Clinical Significance in Colorectal Cancer. Front Immunol (2019) 10.1875 doi: 10.3389/fimmu.2019.01875 31481956 PMC6710360

[214] Selders GS Fetz AE Radic MZ Bowlin GL. An Overview of the Role of Neutrophils in Innate Immunity, Inflammation and Host-Biomaterial Integration. Regener Biomater (2017) 4:55–68. doi: 10.1093/rb/rbw041 PMC5274707 28149530

[215] Suárez-Sánchez FJ Lequerica-Fernández P Suárez-Canto J Rodrigo JP Rodriguez-Santamarta T Domínguez-Iglesias F. Macrophages in Oral Carcinomas: Relationship With Cancer Stem Cell Markers and PD-L1 Expression. Cancers (2020) 12.1764 doi: 10.3390/cancers12071764 PMC7408350 32630659

[216] Mori K Hiroi M Shimada J Ohmori Y. Infiltration of M2 Tumor-Associated Macrophages in Oral Squamous Cell Carcinoma Correlates With Tumor Malignancy. Cancers (2011) 3:3726–39. doi: 10.3390/cancers3043726 PMC3763393 24213108

[217] Wang S Sun M Gu C Wang X Chen D Zhao E. Expression of CD163, Interleukin-10, and Interferon-Gamma in Oral Squamous Cell Carcinoma: Mutual Relationships and Prognostic Implications. Eur J Oral Sci (2014) 122:202–9. doi: 10.1111/eos.12131 24796206

[218] Ye X Zhang J Lu R Zhou G. Signal Regulatory Protein α Associated With the Progression of Oral Leukoplakia and Oral Squamous Cell Carcinoma Regulates Phenotype Switch of Macrophages. Oncotarget (2016) 7:81305–21. doi: 10.18632/oncotarget.12874 PMC5348394 27793032

[219] Haque ASMR Moriyama M Kubota K Ishiguro N Sakamoto M Chinju A. CD206(+) Tumor-Associated Macrophages Promote Proliferation and Invasion in Oral Squamous Cell Carcinoma *via* EGF Production. Sci Rep (2019) 9:14611–. doi: 10.1038/s41598-019-51149-1 PMC6787225 31601953

[220] Fujii N Shomori K Shiomi T Nakabayashi M Takeda C Ryoke K. Cancer-Associated Fibroblasts and CD163-Positive Macrophages in Oral Squamous Cell Carcinoma: Their Clinicopathological and Prognostic Significance. J Oral Pathol Med (2012) 41:444–51. doi: 10.1111/j.1600-0714.2012.01127.x 22296275

[221] Kouketsu A Sato I Oikawa M Shimizu Y Saito H Tashiro K. Regulatory T Cells and M2-Polarized Tumour-Associated Macrophages are Associated With the Oncogenesis and Progression of Oral Squamous Cell Carcinoma. Int J Oral Maxillofac Surg (2019) 48:1279–88. doi: 10.1016/j.ijom.2019.04.004 31053518

[222] Weber M Büttner-Herold M Hyckel P Moebius P Distel L Ries J. Small Oral Squamous Cell Carcinomas With Nodal Lymphogenic Metastasis Show Increased Infiltration of M2 Polarized Macrophages–an Immunohistochemical Analysis. J Craniomaxillofac Surg (2014) 42:1087–94. doi: 10.1016/j.jcms.2014.01.035 24556525

[223] den Toom IJ Mahieu R van Rooij R van Es RJJ Hobbelink MGG Krijger GC. Sentinel Lymph Node Detection in Oral Cancer: A Within-Patient Comparison Between [(99m)Tc]Tc-Tilmanocept and [(99m)Tc]Tc-Nanocolloid. Eur J Nucl Med Mol Imaging (2021) 48:851–8. doi: 10.1007/s00259-020-04984-8 PMC8036184 32839855

[224] Kogure A Kosaka N Ochiya T. Cross-Talk Between Cancer Cells and Their Neighbors *via* miRNA in Extracellular Vesicles: An Emerging Player in Cancer Metastasis. J BioMed Sci (2019) 26:7. doi: 10.1186/s12929-019-0500-6 30634952 PMC6330499

[225] Raghavan S Mehta P Xie Y Lei YL Mehta G. Ovarian Cancer Stem Cells and Macrophages Reciprocally Interact Through the WNT Pathway to Promote Pro-Tumoral and Malignant Phenotypes in 3D Engineered Microenvironments. J Immunother Cancer (2019) 7:190. doi: 10.1186/s40425-019-0666-1 31324218 PMC6642605

[226] Ruffell B Chang-Strachan D Chan V Rosenbusch A Ho CM Pryer N. Macrophage IL-10 Blocks CD8+ T Cell-Dependent Responses to Chemotherapy by Suppressing IL-12 Expression in Intratumoral Dendritic Cells. Cancer Cell (2014) 26:623–37. doi: 10.1016/j.ccell.2014.09.006 PMC4254570 25446896

[227] Baghdadi M Wada H Nakanishi S Abe H Han N Putra WE. Chemotherapy-Induced IL34 Enhances Immunosuppression by Tumor-Associated Macrophages and Mediates Survival of Chemoresistant Lung Cancer Cells. Cancer Res (2016) 76:6030–42. doi: 10.1158/0008-5472.CAN-16-1170 27550451

[228] Shiao SL Ruffell B DeNardo DG Faddegon BA Park CC Coussens LM. TH2-Polarized CD4(+) T Cells and Macrophages Limit Efficacy of Radiotherapy. Cancer Immunol Res (2015) 3:518–25. doi: 10.1158/2326-6066.CIR-14-0232 PMC4420686 25716473

[229] Xu X Ye J Huang C Yan Y Li J. M2 Macrophage-Derived IL6 Mediates Resistance of Breast Cancer Cells to Hedgehog Inhibition. Toxicol Appl Pharmacol (2019) 364:77–82. doi: 10.1016/j.taap.2018.12.013 30578886

[230] Yin Y Yao S Hu Y Feng Y Li M Bian Z. The Immune-Microenvironment Confers Chemoresistance of Colorectal Cancer Through Macrophage-Derived Il6. Clin Cancer Res (2017) 23:7375–87. doi: 10.1158/1078-0432.CCR-17-1283 28928161

[231] Zhu X Shen H Yin X Long L Chen X Feng F. IL-6r/STAT3/miR-204 Feedback Loop Contributes to Cisplatin Resistance of Epithelial Ovarian Cancer Cells. Oncotarget (2017) 8:39154–66. doi: 10.18632/oncotarget.16610 PMC5503602 28388577

[232] Ngambenjawong C Gustafson HH Pun SH. Progress in Tumor-Associated Macrophage (TAM)-Targeted Therapeutics. Adv Drug Delivery Rev (2017) 114:206–21. doi: 10.1016/j.addr.2017.04.010 PMC5581987 28449873

[233] Laviron M Boissonnas A. Ontogeny of Tumor-Associated Macrophages. Front Immunol (2019) 10:1799. doi: 10.3389/fimmu.2019.01799 31417566 PMC6684758

[234] Poh AR Ernst M. Targeting Macrophages in Cancer: From Bench to Bedside. Front Oncol (2018) 8:49. doi: 10.3389/fonc.2018.00049 29594035 PMC5858529

[235] Castell SD Harman MF Morón G Maletto BA Pistoresi-Palencia MC. Neutrophils Which Migrate to Lymph Nodes Modulate CD4(+) T Cell Response by a PD-L1 Dependent Mechanism. Front Immunol (2019) 10:105. doi: 10.3389/fimmu.2019.00105 30761151 PMC6362305

[236] Sun R Xiong Y Liu H Gao C Su L Weng J. Tumor-Associated Neutrophils Suppress Antitumor Immunity of NK Cells Through the PD-L1/PD-1 Axis. Transl Oncol (2020) 13:100825. doi: 10.1016/j.tranon.2020.100825 32698059 PMC7372151

[237] Lewkowicz N Mycko MP Przygodzka P Ćwiklińska H Cichalewska M Matysiak M. Induction of Human IL-10-Producing Neutrophils by LPS-Stimulated Treg Cells and IL-10. Mucosal Immunol (2016) 9:364–78. doi: 10.1038/mi.2015.66 26220165

[238] Guerriero JL Sotayo A Ponichtera HE Castrillon JA Pourzia AL Schad S. Class IIa HDAC Inhibition Reduces Breast Tumours and Metastases Through Anti-Tumour Macrophages. Nature (2017) 543:428–32. doi: 10.1038/nature21409 PMC8170529 28273064

[239] Hu Y He MY Zhu LF Yang CC Zhou ML Wang Q. Tumor-Associated Macrophages Correlate With the Clinicopathological Features and Poor Outcomes *via* Inducing Epithelial to Mesenchymal Transition in Oral Squamous Cell Carcinoma. J Exp Clin Cancer Res (2016) 35:12. doi: 10.1186/s13046-015-0281-z 26769084 PMC4714460

[240] Seminerio I Kindt N Descamps G Bellier J Lechien JR Mat Q. High Infiltration of CD68+ Macrophages is Associated With Poor Prognoses of Head and Neck Squamous Cell Carcinoma Patients and is Influenced by Human Papillomavirus. Oncotarget (2018) 9:11046–59. doi: 10.18632/oncotarget.24306 PMC5834277 29541395

[241] Bagul N Roy S Ganjre A Kathariya R Meher A Singh P. Quantitative Assessment of Tumor Associated Macrophages in Head and Neck Squamous Cell Carcinoma Using CD68 Marker: An Immunohistochemical Study. J Clin Diagn Res (2016) 10:Zc81–4. doi: 10.7860/JCDR/2016/13924.7670 PMC4866257 27190959

[242] He KF Zhang L Huang CF Ma SR Wang YF Wang WM. CD163+ Tumor-Associated Macrophages Correlated With Poor Prognosis and Cancer Stem Cells in Oral Squamous Cell Carcinoma. BioMed Res Int (2014) 2014:838632. doi: 10.1155/2014/838632 24883329 PMC4032721

[243] Haque A Moriyama M Kubota K Ishiguro N Sakamoto M Chinju A. CD206(+) Tumor-Associated Macrophages Promote Proliferation and Invasion in Oral Squamous Cell Carcinoma *via* EGF Production. Sci Rep (2019) 9:14611. doi: 10.1038/s41598-019-51149-1 31601953 PMC6787225

[244] Sugimura K Miyata H Tanaka K Takahashi T Kurokawa Y Yamasaki M. High Infiltration of Tumor-Associated Macrophages is Associated With a Poor Response to Chemotherapy and Poor Prognosis of Patients Undergoing Neoadjuvant Chemotherapy for Esophageal Cancer. J Surg Oncol (2015) 111:752–9. doi: 10.1002/jso.23881 25752960

[245] Balermpas P Rodel F Liberz R Oppermann J Wagenblast J Ghanaati S. Head and Neck Cancer Relapse After Chemoradiotherapy Correlates With CD163+ Macrophages in Primary Tumour and CD11b+ Myeloid Cells in Recurrences. Br J Cancer (2014) 111:1509–18. doi: 10.1038/bjc.2014.446 PMC4200089 25093488

[246] Kratochvill F Neale G Haverkamp JM Van de Velde LA Smith AM Kawauchi D. TNF Counterbalances the Emergence of M2 Tumor Macrophages. Cell Rep (2015) 12:1902–14. doi: 10.1016/j.celrep.2015.08.033 PMC4581986 26365184

[247] Biswas SK Gangi L Paul S Schioppa T Saccani A Sironi M. and Unique Transcriptional Program Expressed by Tumor-Associated Macrophages (Defective NF-kappaB and Enhanced IRF-3/STAT1 Activation). Blood (2006) 107:2112–22. doi: 10.1182/blood-2005-01-0428 16269622

[248] Liu CY Xu JY Shi XY Huang W Ruan TY Xie P. M2-Polarized Tumor-Associated Macrophages Promoted Epithelial-Mesenchymal Transition in Pancreatic Cancer Cells, Partially Through TLR4/IL-10 Signaling Pathway. Lab Invest (2013) 93:844–54. doi: 10.1038/labinvest.2013.69 23752129

[249] Rodriguez PC Hernandez CP Quiceno D Dubinett SM Zabaleta J Ochoa JB. Arginase I in Myeloid Suppressor Cells is Induced by COX-2 in Lung Carcinoma. J Exp Med (2005) 202:931–9. doi: 10.1084/jem.20050715 PMC2213169 16186186

[250] Van Ginderachter JA Meerschaut S Liu Y Brys L De Groeve K Hassanzadeh Ghassabeh G. Peroxisome Proliferator-Activated Receptor Gamma (PPARgamma) Ligands Reverse CTL Suppression by Alternatively Activated (M2) Macrophages in Cancer. Blood (2006) 108:525–35. doi: 10.1182/blood-2005-09-3777 16527895

[251] Zhang M He Y Sun X Li Q Wang W Zhao A. A High M1/M2 Ratio of Tumor-Associated Macrophages is Associated With Extended Survival in Ovarian Cancer Patients. J Ovarian Res (2014) 7:19. doi: 10.1186/1757-2215-7-19 24507759 PMC3939626

[252] Hourani T Holden JA Li W Lenzo JC Hadjigol S O’Brien-Simpson NM. Tumor Associated Macrophages: Origin, Recruitment, Phenotypic Diversity, and Targeting. Front Oncol (2021) 11:788365. doi: 10.3389/fonc.2021.788365 34988021 PMC8722774

[253] Dan H Liu S Liu J Liu D Yin F Wei Z. RACK1 Promotes Cancer Progression by Increasing the M2/M1 Macrophage Ratio *via* the NF-κb Pathway in Oral Squamous Cell Carcinoma. Mol Oncol (2020) 14:795–807. doi: 10.1002/1878-0261.12644 31997535 PMC7138402

[254] Bennouna S Denkers EY. Microbial Antigen Triggers Rapid Mobilization of TNF-Alpha to the Surface of Mouse Neutrophils Transforming Them Into Inducers of High-Level Dendritic Cell TNF-Alpha Production. J Immunol (2005) 174:4845–51. doi: 10.4049/jimmunol.174.8.4845 15814711

[255] Borregaard N Sørensen OE Theilgaard-Mönch K. Neutrophil Granules: A Library of Innate Immunity Proteins. Trends Immunol (2007) 28:340–5. doi: 10.1016/j.it.2007.06.002 17627888

[256] Yang D Oppenheim JJ. Antimicrobial Proteins Act as “Alarmins”. Joint Immune defense Arthritis Rheum (2004) 50:3401–3. doi: 10.1002/art.20604 15529365

[257] Oppenheim JJ Yang D. Alarmins: Chemotactic Activators of Immune Responses. Curr Opin Immunol (2005) 17:359–65. doi: 10.1016/j.coi.2005.06.002 15955682

[258] Yang D de la Rosa G Tewary P Oppenheim JJ. Alarmins Link Neutrophils and Dendritic Cells. Trends Immunol (2009) 30:531–7. doi: 10.1016/j.it.2009.07.004 PMC2767430 19699678

[259] Yang D Biragyn A Hoover DM Lubkowski J Oppenheim JJ. Multiple Roles of Antimicrobial Defensins, Cathelicidins, and Eosinophil-Derived Neurotoxin in Host Defense. Annu Rev Immunol (2004) 22:181–215. doi: 10.1146/annurev.immunol.22.012703.104603 15032578

[260] Yang D Chen Q Chertov O Oppenheim JJ. Human Neutrophil Defensins Selectively Chemoattract Naive T and Immature Dendritic Cells. J Leukoc Biol (2000) 68:9–14. 10914484

[261] Biragyn A Ruffini PA Leifer CA Klyushnenkova E Shakhov A Chertov O. Toll-Like Receptor 4-Dependent Activation of Dendritic Cells by Beta-Defensin 2. Science (2002) 298:1025–9. doi: 10.1126/science.1075565 12411706

[262] Yang D Chen Q Yang H Tracey KJ Bustin M Oppenheim JJ. High Mobility Group Box-1 Protein Induces the Migration and Activation of Human Dendritic Cells and Acts as an Alarmin. J Leukoc Biol (2007) 81:59–66. doi: 10.1189/jlb.0306180 16966386

[263] Megiovanni AM Sanchez F Robledo-Sarmiento M Morel C Gluckman JC Boudaly S. Polymorphonuclear Neutrophils Deliver Activation Signals and Antigenic Molecules to Dendritic Cells: A New Link Between Leukocytes Upstream of T Lymphocytes. J Leukoc Biol (2006) 79:977–88. doi: 10.1189/jlb.0905526 16501052

[264] van Gisbergen KP Ludwig IS Geijtenbeek TB van Kooyk Y. Interactions of DC-SIGN With Mac-1 and CEACAM1 Regulate Contact Between Dendritic Cells and Neutrophils. FEBS Lett (2005) 579:6159–68. doi: 10.1016/j.febslet.2005.09.089 16246332

[265] van Gisbergen KP Sanchez-Hernandez M Geijtenbeek TB van Kooyk Y. Neutrophils Mediate Immune Modulation of Dendritic Cells Through Glycosylation-Dependent Interactions Between Mac-1 and DC-SIGN. J Exp Med (2005) 201:1281–92. doi: 10.1084/jem.20041276 PMC2213143 15837813

[266] Singer BB Klaile E Scheffrahn I Müller MM Kammerer R Reutter W. CEACAM1 (CD66a) Mediates Delay of Spontaneous and Fas Ligand-Induced Apoptosis in Granulocytes. Eur J Immunol (2005) 35:1949–59. doi: 10.1002/eji.200425691 15909305

[267] Gardner A Ruffell B. Dendritic Cells and Cancer Immunity. Trends Immunol (2016) 37:855–65. doi: 10.1016/j.it.2016.09.006 PMC5135568 27793569

[268] Böttcher JP Reis e Sousa C. The Role of Type 1 Conventional Dendritic Cells in Cancer Immunity. Trends Cancer (2018) 4:784–92. doi: 10.1016/j.trecan.2018.09.001 PMC6207145 30352680

[269] Engelhardt JJ Boldajipour B Beemiller P Pandurangi P Sorensen C Werb Z. Marginating Dendritic Cells of the Tumor Microenvironment Cross-Present Tumor Antigens and Stably Engage Tumor-Specific T Cells. Cancer Cell (2012) 21:402–17. doi: 10.1016/j.ccr.2012.01.008 PMC3311997 22439936

[270] Tecchio C Cassatella MA. Neutrophil-Derived Chemokines on the Road to Immunity. Semin Immunol (2016) 28:119–28. doi: 10.1016/j.smim.2016.04.003 PMC7129466 27151246

[271] Dudek AM Martin S Garg AD Agostinis P. Immature, Semi-Mature, and Fully Mature Dendritic Cells: Toward a DC-Cancer Cells Interface That Augments Anticancer Immunity. Front Immunol (2013) 4:438. doi: 10.3389/fimmu.2013.00438 24376443 PMC3858649

[272] Suryawanshi A Hussein MS Prasad PD Manicassamy S. Wnt Signaling Cascade in Dendritic Cells and Regulation of Anti-Tumor Immunity. Front Immunol (2020) 11:122. doi: 10.3389/fimmu.2020.00122 32132993 PMC7039855

[273] Broz ML Binnewies M Boldajipour B Nelson AE Pollack JL Erle DJ. Dissecting the Tumor Myeloid Compartment Reveals Rare Activating Antigen-Presenting Cells Critical for T Cell Immunity. Cancer Cell (2014) 26:638–52. doi: 10.1016/j.ccell.2014.09.007 PMC4254577 25446897

[274] Fuertes MB Woo SR Burnett B Fu YX Gajewski TF. Type I Interferon Response and Innate Immune Sensing of Cancer. Trends Immunol (2013) 34:67–73. doi: 10.1016/j.it.2012.10.004 23122052 PMC3565059

[275] Parker BS Rautela J Hertzog PJ. Antitumour Actions of Interferons: Implications for Cancer Therapy. Nat Rev Cancer (2016) 16:131–44. doi: 10.1038/nrc.2016.14 26911188

[276] Fuertes MB Kacha AK Kline J Woo SR Kranz DM Murphy KM. Host Type I IFN Signals are Required for Antitumor CD8+ T Cell Responses Through CD8{alpha}+ Dendritic Cells. J Exp Med (2011) 208:2005–16. doi: 10.1084/jem.20101159 PMC3182064 21930765

[277] Woo SR Fuertes MB Corrales L Spranger S Furdyna MJ Leung MY. STING-Dependent Cytosolic DNA Sensing Mediates Innate Immune Recognition of Immunogenic Tumors. Immunity (2014) 41:830–42. doi: 10.1016/j.immuni.2014.10.017 PMC4384884 25517615

[278] Spranger S Dai D Horton B Gajewski TF. Tumor-Residing Batf3 Dendritic Cells Are Required for Effector T Cell Trafficking and Adoptive T Cell Therapy. Cancer Cell (2017) 31:711–23.e4. doi: 10.1016/j.ccell.2017.04.003 28486109 PMC5650691

[279] Metzemaekers M Vanheule V Janssens R Struyf S Proost P. Overview of the Mechanisms That May Contribute to the Non-Redundant Activities of Interferon-Inducible CXC Chemokine Receptor 3 Ligands. Front Immunol (2017) 8.1970 doi: 10.3389/fimmu.2017.01970 29379506 PMC5775283

[280] Ichikawa A Kuba K Morita M Chida S Tezuka H Hara H. CXCL10-CXCR3 Enhances the Development of Neutrophil-Mediated Fulminant Lung Injury of Viral and Nonviral Origin. Am J Respir Crit Care Med (2013) 187:65–77. doi: 10.1164/rccm.201203-0508OC 23144331 PMC3927876

[281] Tan P He L Xing C Mao J Yu X Zhu M. Myeloid Loss of Beclin 1 Promotes PD-L1hi Precursor B Cell Lymphoma Development. J Clin Invest (2019) 129:5261–77. doi: 10.1172/JCI127721 PMC6877338 31503548

[282] Tran Janco JM Lamichhane P Karyampudi L Knutson KL. Tumor-Infiltrating Dendritic Cells in Cancer Pathogenesis. J Immunol (2015) 194:2985–91. doi: 10.4049/jimmunol.1403134 PMC4369768 25795789

[283] Spranger S Bao R Gajewski TF. Melanoma-Intrinsic β-Catenin Signalling Prevents Anti-Tumour Immunity. Nature (2015) 523:231–5. doi: 10.1038/nature14404 25970248

[284] Minns D Smith KJ Findlay EG. Orchestration of Adaptive T Cell Responses by Neutrophil Granule Contents. Mediators Inflammation (2019) 2019:8968943. doi: 10.1155/2019/8968943 PMC6431490 30983883

[285] de la Rosa G Yang D Tewary P Varadhachary A Oppenheim JJ. Lactoferrin Acts as an Alarmin to Promote the Recruitment and Activation of APCs and Antigen-Specific Immune Responses. J Immunol (2008) 180:6868–76. doi: 10.4049/jimmunol.180.10.6868 PMC2408856 18453607

[286] Odobasic D Kitching AR Yang Y O’Sullivan KM Muljadi RC Edgtton KL. Neutrophil Myeloperoxidase Regulates T-Cell-Driven Tissue Inflammation in Mice by Inhibiting Dendritic Cell Function. Blood (2013) 121:4195–204. doi: 10.1182/blood-2012-09-456483 23509155

[287] Barry KC Hsu J Broz ML Cueto FJ Binnewies M Combes AJ. A Natural Killer-Dendritic Cell Axis Defines Checkpoint Therapy-Responsive Tumor Microenvironments. Nat Med (2018) 24:1178–91. doi: 10.1038/s41591-018-0085-8 PMC6475503 29942093

[288] Zelenay S van der Veen AG Böttcher JP Snelgrove KJ Rogers N Acton SE. Cyclooxygenase-Dependent Tumor Growth Through Evasion of Immunity. Cell (2015) 162:1257–70. doi: 10.1016/j.cell.2015.08.015 PMC4597191 26343581

[289] Tang M Diao J Cattral MS. Molecular Mechanisms Involved in Dendritic Cell Dysfunction in Cancer. Cell Mol Life Sci (2017) 74:761–76. doi: 10.1007/s00018-016-2317-8 PMC11107728 27491428

[290] Zong J Keskinov AA Shurin GV Shurin MR. Tumor-Derived Factors Modulating Dendritic Cell Function. Cancer Immunol Immunother (2016) 65:821–33. doi: 10.1007/s00262-016-1820-y PMC11028482 26984847

[291] Menetrier-Caux C Montmain G Dieu MC Bain C Favrot MC Caux C. Inhibition of the Differentiation of Dendritic Cells From CD34(+) Progenitors by Tumor Cells: Role of Interleukin-6 and Macrophage Colony-Stimulating Factor. Blood (1998) 92:4778–91. doi: 10.1182/blood.V92.12.4778.424k14_4778_4791 9845545

[292] Pahne-Zeppenfeld J Schröer N Walch-Rückheim B Oldak M Gorter A Hegde S. Cervical Cancer Cell-Derived Interleukin-6 Impairs CCR7-Dependent Migration of MMP-9-Expressing Dendritic Cells. Int J Cancer (2014) 134:2061–73. doi: 10.1002/ijc.28549 24136650

[293] Chomarat P Banchereau J Davoust J Palucka AK. IL-6 Switches the Differentiation of Monocytes From Dendritic Cells to Macrophages. Nat Immunol (2000) 1:510–4. doi: 10.1038/82763 11101873

[294] Hargadon KM. Tumor-Altered Dendritic Cell Function: Implications for Anti-Tumor Immunity. Front Immunol (2013) 4:192. doi: 10.3389/fimmu.2013.00192 23874338 PMC3708450

[295] Ohm JE Shurin MR Esche C Lotze MT Carbone DP Gabrilovich DI. Effect of Vascular Endothelial Growth Factor and FLT3 Ligand on Dendritic Cell Generation *In Vivo* . J Immunol (1999) 163:3260–8. 10477595

[296] Larmonier N Marron M Zeng Y Cantrell J Romanoski A Sepassi M. Tumor-Derived CD4(+)CD25(+) Regulatory T Cell Suppression of Dendritic Cell Function Involves TGF-Beta and IL-10. Cancer Immunol Immunother (2007) 56:48–59. doi: 10.1007/s00262-006-0160-8 16612596 PMC11030031

[297] Caldeira PC Vieira É LM Sousa AA Teixeira AL Aguiar MCF. Immunophenotype of Neutrophils in Oral Squamous Cell Carcinoma Patients. J Oral Pathol Med (2017) 46:703–9. doi: 10.1111/jop.12575 28370402

[298] Yang AS Lattime EC. Tumor-Induced Interleukin 10 Suppresses the Ability of Splenic Dendritic Cells to Stimulate CD4 and CD8 T-Cell Responses. Cancer Res (2003) 63:2150–7. 12727833

[299] Steinbrink K Jonuleit H Müller G Schuler G Knop J Enk AH. Interleukin-10-Treated Human Dendritic Cells Induce a Melanoma-Antigen-Specific Anergy in CD8(+) T Cells Resulting in a Failure to Lyse Tumor Cells. Blood (1999) 93:1634–42. doi: 10.1182/blood.V93.5.1634 10029592

[300] Kel JM Girard-Madoux MJ Reizis B Clausen BE. TGF-Beta is Required to Maintain the Pool of Immature Langerhans Cells in the Epidermis. J Immunol (2010) 185:3248–55. doi: 10.4049/jimmunol.1000981 20713882

[301] Obeid M Tesniere A Ghiringhelli F Fimia GM Apetoh L Perfettini JL. Calreticulin Exposure Dictates the Immunogenicity of Cancer Cell Death. Nat Med (2007) 13:54–61. doi: 10.1038/nm1523 17187072

[302] Yang H Wang H Chavan SS Andersson U. High Mobility Group Box Protein 1 (HMGB1): The Prototypical Endogenous Danger Molecule. Mol Med (Cambridge Mass.) (2015) 21 Suppl 1:S6–S12. doi: 10.2119/molmed.2015.00087 26605648 PMC4661054

[303] Yanai H Ban T Wang Z Choi MK Kawamura T Negishi H. HMGB Proteins Function as Universal Sentinels for Nucleic-Acid-Mediated Innate Immune Responses. Nature (2009) 462:99–103. doi: 10.1038/nature08512 19890330

[304] Chiba S Baghdadi M Akiba H Yoshiyama H Kinoshita I Dosaka-Akita H. Tumor-Infiltrating DCs Suppress Nucleic Acid-Mediated Innate Immune Responses Through Interactions Between the Receptor TIM-3 and the Alarmin HMGB1. Nat Immunol (2012) 13:832–42. doi: 10.1038/ni.2376 PMC3622453 22842346

[305] Solinas C De Silva P Bron D Willard-Gallo K Sangiolo D. Significance of TIM3 Expression in Cancer: From Biology to the Clinic. Semin Oncol (2019) 46:372–9. doi: 10.1053/j.seminoncol.2019.08.005 31733828

[306] Ilie M Hofman V Ortholan C Bonnetaud C Coëlle C Mouroux J. Predictive Clinical Outcome of the Intratumoral CD66b-Positive Neutrophil-to-CD8-Positive T-Cell Ratio in Patients With Resectable Nonsmall Cell Lung Cancer. Cancer (2012) 118:1726–37. doi: 10.1002/cncr.26456 21953630

[307] Shen M Jiang K Sui Y Xu Z Cui H Wang Y. Characterization of CD66b and its Relationship Between Immune Checkpoints and Their Synergistic Impact in the Prognosis of Surgically Resected Lung Adenocarcinoma. Lung Cancer (2021) 160:84–91. doi: 10.1016/j.lungcan.2021.08.012 34479175

[308] Maurya N Gujar R Gupta M Yadav V Verma S Sen P. Immunoregulation of Dendritic Cells by the Receptor T Cell Ig and Mucin Protein-3 *via* Bruton’s Tyrosine Kinase and C-Src. J Immunol (2014) 193:3417–25. doi: 10.4049/jimmunol.1400395 25172495

[309] Barclay AN Van den Berg TK. The Interaction Between Signal Regulatory Protein Alpha (Sirpα) and CD47: Structure, Function, and Therapeutic Target. Annu Rev Immunol (2014) 32:25–50. doi: 10.1146/annurev-immunol-032713-120142 24215318

[310] Blazar BR Lindberg FP Ingulli E Panoskaltsis-Mortari A Oldenborg PA Iizuka K. CD47 (Integrin-Associated Protein) Engagement of Dendritic Cell and Macrophage Counterreceptors is Required to Prevent the Clearance of Donor Lymphohematopoietic Cells. J Exp Med (2001) 194:541–9. doi: 10.1084/jem.194.4.541 PMC2193501 11514609

[311] Majeti R Chao MP Alizadeh AA Pang WW Jaiswal S Gibbs KD Jr.. CD47 is an Adverse Prognostic Factor and Therapeutic Antibody Target on Human Acute Myeloid Leukemia Stem Cells. Cell (2009) 138:286–99. doi: 10.1016/j.cell.2009.05.045 PMC2726837 19632179

[312] Xu MM Pu Y Han D Shi Y Cao X Liang H. Dendritic Cells But Not Macrophages Sense Tumor Mitochondrial DNA for Cross-Priming Through Signal Regulatory Protein α Signaling. Immunity (2017) 47:363–73.e5. doi: 10.1016/j.immuni.2017.07.016 28801234 PMC5564225

[313] Fang C Mo F Liu L Du J Luo M Men K. Oxidized Mitochondrial DNA Sensing by STING Signaling Promotes the Antitumor Effect of an Irradiated Immunogenic Cancer Cell Vaccine. Cell Mol Immunol (2021) 18:2211–23. doi: 10.1038/s41423-020-0456-1 PMC8429462 32398808

[314] Cannarile MA Weisser M Jacob W Jegg AM Ries CH Rüttinger D. Colony-Stimulating Factor 1 Receptor (CSF1R) Inhibitors in Cancer Therapy. J Immunother Cancer (2017) 5:53. doi: 10.1186/s40425-017-0257-y 28716061 PMC5514481

[315] Herber DL Cao W Nefedova Y Novitskiy SV Nagaraj S Tyurin VA. Lipid Accumulation and Dendritic Cell Dysfunction in Cancer. Nat Med (2010) 16:880–6. doi: 10.1038/nm.2172 PMC2917488 20622859

[316] Kawakami Y Yaguchi T Sumimoto H Kudo-Saito C Iwata-Kajihara T Nakamura S. Improvement of Cancer Immunotherapy by Combining Molecular Targeted Therapy. Front Oncol (2013) 3:. doi: 10.3389/fonc.2013.00136 PMC3664832 23755373

[317] Kerdidani D Chouvardas P Arjo AR Giopanou I Ntaliarda G Guo YA. Wnt1 Silences Chemokine Genes in Dendritic Cells and Induces Adaptive Immune Resistance in Lung Adenocarcinoma. Nat Commun (2019) 10:1405. doi: 10.1038/s41467-019-09370-z 30926812 PMC6441097

[318] Hong Y Manoharan I Suryawanshi A Shanmugam A Swafford D Ahmad S. Deletion of LRP5 and LRP6 in Dendritic Cells Enhances Antitumor Immunity. Oncoimmunology (2016) 5:e1115941. doi: 10.1080/2162402X.2015.1115941 27141399 PMC4839371

[319] Oderup C LaJevic M Butcher EC. Canonical and Noncanonical Wnt Proteins Program Dendritic Cell Responses for Tolerance. J Immunol (2013) 190:6126–34. doi: 10.4049/jimmunol.1203002 PMC3698971 23677472

[320] Clevers H Nusse R. Wnt/β-Catenin Signaling and Disease. Cell (2012) 149:1192–205. doi: 10.1016/j.cell.2012.05.012 22682243

[321] Rapp J Jaromi L Kvell K Miskei G Pongracz JE. WNT Signaling - Lung Cancer is No Exception. Respir Res (2017) 18:167–. doi: 10.1186/s12931-017-0650-6 PMC5584342 28870231

[322] Asem MS Buechler S Wates RB Miller DL Stack MS. Wnt5a Signaling in Cancer. Cancers (2016) 8:79. doi: 10.3390/cancers8090079 PMC5040981 27571105

[323] Reyes M Flores T Betancur D Peña-Oyarzún D Torres VA. Wnt/β-Catenin Signaling in Oral Carcinogenesis. Int J Mol Sci (2020) 21:4682–704. doi: 10.3390/ijms21134682 PMC7369957 32630122

[324] Yang D Li S Duan X Ren J Liang S Yakoumatos L. TLR4 Induced Wnt3a-Dvl3 Restrains the Intensity of Inflammation and Protects Against Endotoxin-Driven Organ Failure Through GSK3β/β-Catenin Signaling. Mol Immunol (2020) 118:153–64. doi: 10.1016/j.molimm.2019.12.013 PMC7035959 31884387

[325] Guo Y Mishra A Howland E Zhao C Shukla D Weng T. Platelet-Derived Wnt Antagonist Dickkopf-1 is Implicated in ICAM-1/VCAM-1-Mediated Neutrophilic Acute Lung Inflammation. Blood (2015) 126:2220–9. doi: 10.1182/blood-2015-02-622233 PMC4635118 26351298

[326] Wang Y Sano S Oshima K Sano M Watanabe Y Katanasaka Y. Wnt5a-Mediated Neutrophil Recruitment Has an Obligatory Role in Pressure Overload-Induced Cardiac Dysfunction. Circulation (2019) 140:487–99. doi: 10.1161/CIRCULATIONAHA.118.038820 PMC6684855 31170826

[327] Jung YS Lee HY Kim SD Park JS Kim JK Suh PG. Wnt5a Stimulates Chemotactic Migration and Chemokine Production in Human Neutrophils. Exp Mol Med (2013) 45:e27. doi: 10.1038/emm.2013.48 23764954 PMC3701286

[328] Shan Q Takabatake K Omori H Kawai H Oo MW Nakano K. Stromal Cells in the Tumor Microenvironment Promote the Progression of Oral Squamous Cell Carcinoma. Int J Oncol (2021) 59:1–17. doi: 10.3892/ijo.2021.5252 PMC8360621 34368860

[329] Provenzano PP Eliceiri KW Campbell JM Inman DR White JG Keely PJ. Collagen Reorganization at the Tumor-Stromal Interface Facilitates Local Invasion. BMC Med (2006) 4:38. doi: 10.1186/1741-7015-4-38 17190588 PMC1781458

[330] Levental KR Yu H Kass L Lakins JN Egeblad M Erler JT. Matrix Crosslinking Forces Tumor Progression by Enhancing Integrin Signaling. Cell (2009) 139:891–906. doi: 10.1016/j.cell.2009.10.027 19931152 PMC2788004

[331] Zhu Y Huang Y Ji Q Fu S Gu J Tai N. Interplay Between Extracellular Matrix and Neutrophils in Diseases. J Immunol Res (2021) 2021:8243378. doi: 10.1155/2021/8243378 34327245 PMC8302397

[332] Moroy G Alix AJ Sapi J Hornebeck W Bourguet E. Neutrophil Elastase as a Target in Lung Cancer. Anticancer Agents Med Chem (2012) 12:565–79. doi: 10.2174/187152012800617696 22263788

[333] Albrengues J Shields MA Ng D Park CG Ambrico A Poindexter ME. Neutrophil Extracellular Traps Produced During Inflammation Awaken Dormant Cancer Cells in Mice. Science (2018) 361:1–30. doi: 10.1126/science.aao4227 PMC6777850 30262472

[334] Jones PL Cowan KN Rabinovitch M. Tenascin-C, Proliferation and Subendothelial Fibronectin in Progressive Pulmonary Vascular Disease. Am J Pathol (1997) 150:1349–60. PMC1858188 9094991

[335] Ong CW Elkington PT Brilha S Ugarte-Gil C Tome-Esteban MT Tezera LB. Neutrophil-Derived MMP-8 Drives AMPK-Dependent Matrix Destruction in Human Pulmonary Tuberculosis. PLoS Pathog (2015) 11:e1004917. doi: 10.1371/journal.ppat.1004917 25996154 PMC4440706

[336] Ong CWM Fox K Ettorre A Elkington PT Friedland JS. Hypoxia Increases Neutrophil-Driven Matrix Destruction After Exposure to Mycobacterium Tuberculosis. Sci Rep (2018) 8:11475. doi: 10.1038/s41598-018-29659-1 30065292 PMC6068197

[337] Germann M Zangger N Sauvain MO Sempoux C Bowler AD Wirapati P. Neutrophils Suppress Tumor-Infiltrating T Cells in Colon Cancer *via* Matrix Metalloproteinase-Mediated Activation of Tgfβ. EMBO Mol Med (2020) 12:e10681. doi: 10.15252/emmm.201910681 31793740 PMC6949488

[338] Kudo T Kigoshi H Hagiwara T Takino T Yamazaki M Yui S. a Neutrophil Protease, Induces Compact Cell-Cell Adhesion in MCF-7 Human Breast Cancer Cells. Mediators Inflammation (2009) 2009:850940. doi: 10.1155/2009/850940 PMC2775934 19920860

[339] Ziober AF Falls EM Ziober BL. The Extracellular Matrix in Oral Squamous Cell Carcinoma: Friend or Foe? Head Neck (2006) 28:740–9. doi: 10.1002/hed.20382 16649214

[340] Agarwal P Ballabh R. Expression of Type IV Collagen in Different Histological Grades of Oral Squamous Cell Carcinoma: An Immunohistochemical Study. J Cancer Res Ther (2013) 9:272–5. doi: 10.4103/0973-1482.113382 23771372

[341] Shruthy R Sharada P Swaminathan U Nagamalini B. Immunohistochemical Expression of Basement Membrane Laminin in Histological Grades of Oral Squamous Cell Carcinoma: A Semiquantitative Analysis. J Oral Maxillofac Pathol (2013) 17:185–9. doi: 10.4103/0973-029X.119755 PMC3830224 24250076

[342] Firth NA Reade PC. The Prognosis of Oral Mucosal Squamous Cell Carcinomas: A Comparison of Clinical and Histopathological Grading and of Laminin and Type IV Collagen Staining. Aust Dent J (1996) 41:83–6. doi: 10.1111/j.1834-7819.1996.tb05918.x 8670039

[343] Glogauer JE Sun CX Bradley G Magalhaes MA. Neutrophils Increase Oral Squamous Cell Carcinoma Invasion Through an Invadopodia-Dependent Pathway. Cancer Immunol Res (2015) 3:1218–26. doi: 10.1158/2326-6066.CIR-15-0017 26112922

[344] Harada T Shinohara M Nakamura S Oka M. An Immunohistochemical Study of the Extracellular Matrix in Oral Squamous Cell Carcinoma and its Association With Invasive and Metastatic Potential. Virchows Arch (1994) 424:257–66. doi: 10.1007/BF00194609 7514477

[345] Jia CC Wang TT Liu W Fu BS Hua X Wang GY. Cancer-Associated Fibroblasts From Hepatocellular Carcinoma Promote Malignant Cell Proliferation by HGF Secretion. PLoS One (2013) 8:e63243. doi: 10.1371/journal.pone.0063243 23667593 PMC3647063

[346] Luker KE Lewin SA Mihalko LA Schmidt BT Winkler JS Coggins NL. Scavenging of CXCL12 by CXCR7 Promotes Tumor Growth and Metastasis of CXCR4-Positive Breast Cancer Cells. Oncogene (2012) 31:4750–8. doi: 10.1038/onc.2011.633 PMC3337948 22266857

[347] Augsten M Sjöberg E Frings O Vorrink SU Frijhoff J Olsson E. Cancer-Associated Fibroblasts Expressing CXCL14 Rely Upon NOS1-Derived Nitric Oxide Signaling for Their Tumor-Supporting Properties. Cancer Res (2014) 74:2999–3010. doi: 10.1158/0008-5472.CAN-13-2740 24710408

[348] Wang X Zhang W Sun X Lin Y Chen W. Cancer-Associated Fibroblasts Induce Epithelial-Mesenchymal Transition Through Secreted Cytokines in Endometrial Cancer Cells. Oncol Lett (2018) 15:5694–702. doi: 10.3892/ol.2018.8000 PMC5858056 29563996

[349] Hwaiz R Rahman M Syk I Zhang E Thorlacius H. Rac1-Dependent Secretion of Platelet-Derived CCL5 Regulates Neutrophil Recruitment *via* Activation of Alveolar Macrophages in Septic Lung Injury. J Leukoc Biol (2015) 97:975–84. doi: 10.1189/jlb.4A1214-603R 25717148

[350] Jung DW Che ZM Kim J Kim K Kim KY Williams D. Tumor-Stromal Crosstalk in Invasion of Oral Squamous Cell Carcinoma: A Pivotal Role of CCL7. Int J Cancer (2010) 127:332–44. doi: 10.1002/ijc.25060 19937793

[351] Michalec L Choudhury BK Postlethwait E Wild JS Alam R Lett-Brown M. CCL7 and CXCL10 Orchestrate Oxidative Stress-Induced Neutrophilic Lung Inflammation. J Immunol (2002) 168:846–52. doi: 10.4049/jimmunol.168.2.846 11777981

[352] McCourt M Wang JH Sookhai S Redmond HP. Activated Human Neutrophils Release Hepatocyte Growth Factor/Scatter Factor. Eur J Surg Oncol (2001) 27:396–403. doi: 10.1053/ejso.2001.1133 11417987

[353] Kim SH Choe C Shin YS Jeon MJ Choi SJ Lee J. Human Lung Cancer-Associated Fibroblasts Enhance Motility of non-Small Cell Lung Cancer Cells in Co-Culture. Anticancer Res (2013) 33:2001–9. 23645749

[354] Skalli O Ropraz P Trzeciak A Benzonana G Gillessen D Gabbiani G. A Monoclonal Antibody Against Alpha-Smooth Muscle Actin: A New Probe for Smooth Muscle Differentiation. J Cell Biol (1986) 103:2787–96. doi: 10.1083/jcb.103.6.2787 PMC2114627 3539945

[355] Wonganu B Berger BW. A Specific, Transmembrane Interface Regulates Fibroblast Activation Protein (FAP) Homodimerization, Trafficking and Exopeptidase Activity. Biochim Biophys Acta (2016) 1858:1876–82. doi: 10.1016/j.bbamem.2016.05.001 27155568

[356] Lim KP Cirillo N Hassona Y Wei W Thurlow JK Cheong SC. Fibroblast Gene Expression Profile Reflects the Stage of Tumour Progression in Oral Squamous Cell Carcinoma. J Pathol (2011) 223:459–69. doi: 10.1002/path.2841 21294120

[357] Zhou B Chen WL Wang YY Lin ZY Zhang DM Fan S. A Role for Cancer-Associated Fibroblasts in Inducing the Epithelial-to-Mesenchymal Transition in Human Tongue Squamous Cell Carcinoma. J Oral Pathol Med (2014) 43:585–92. doi: 10.1111/jop.12172 24645915

[358] Kellermann MG Sobral LM da Silva SD Zecchin KG Graner E Lopes MA. Myofibroblasts in the Stroma of Oral Squamous Cell Carcinoma are Associated With Poor Prognosis. Histopathology (2007) 51:849–53. doi: 10.1111/j.1365-2559.2007.02873.x 18042073

[359] Bello IO Vered M Dayan D Dobriyan A Yahalom R Alanen K. Cancer-Associated Fibroblasts, a Parameter of the Tumor Microenvironment, Overcomes Carcinoma-Associated Parameters in the Prognosis of Patients With Mobile Tongue Cancer. Oral Oncol (2011) 47:33–8. doi: 10.1016/j.oraloncology.2010.10.013 21112238

[360] Rosenthal E McCrory A Talbert M Young G Murphy-Ullrich J Gladson C. Elevated Expression of TGF-Beta1 in Head and Neck Cancer-Associated Fibroblasts. Mol Carcinog (2004) 40:116–21. doi: 10.1002/mc.20024 15170816

[361] Knowles LM Stabile LP Egloff AM Rothstein ME Thomas SM Gubish CT. HGF and C-Met Participate in Paracrine Tumorigenic Pathways in Head and Neck Squamous Cell Cancer. Clin Cancer Res (2009) 15:3740–50. doi: 10.1158/1078-0432.CCR-08-3252 PMC3159511 19470725

[362] Johansson AC Ansell A Jerhammar F Lindh MB Grénman R Munck-Wikland E. Cancer-Associated Fibroblasts Induce Matrix Metalloproteinase-Mediated Cetuximab Resistance in Head and Neck Squamous Cell Carcinoma Cells. Mol Cancer Res (2012) 10:1158–68. doi: 10.1158/1541-7786.MCR-12-0030 22809838

[363] Bekes EM Schweighofer B Kupriyanova TA Zajac E Ardi VC Quigley JP. Tumor-Recruited Neutrophils and Neutrophil TIMP-Free MMP-9 Regulate Coordinately the Levels of Tumor Angiogenesis and Efficiency of Malignant Cell Intravasation. Am J Pathol (2011) 179:1455–70. doi: 10.1016/j.ajpath.2011.05.031 PMC3157227 21741942

[364] Song M He J Pan QZ Yang J Zhao J Zhang YJ. Cancer-Associated Fibroblast-Mediated Cellular Crosstalk Supports Hepatocellular Carcinoma Progression. Hepatology (2021) 73:1717–35. doi: 10.1002/hep.31792 33682185

[365] Cheng Y Li H Deng Y Tai Y Zeng K Zhang Y. Cancer-Associated Fibroblasts Induce PDL1+ Neutrophils Through the IL6-STAT3 Pathway That Foster Immune Suppression in Hepatocellular Carcinoma. Cell Death Dis (2018) 9:422. doi: 10.1038/s41419-018-0458-4 29556041 PMC5859264

[366] Fridlender ZG Albelda SM. Tumor-Associated Neutrophils: Friend or Foe? Carcinogenesis (2012) 33:949–55. doi: 10.1093/carcin/bgs123 22425643

[367] Miller JS Lanier LL. Natural Killer Cells in Cancer Immunotherapy. Annu Rev Cancer Biol (2019) 3:77–103. doi: 10.1146/annurev-cancerbio-030518-055653

[368] Kim N Lee HH Lee HJ Choi WS Lee J Kim HS. Natural Killer Cells as a Promising Therapeutic Target for Cancer Immunotherapy. Arch Pharm Res (2019) 42:591–606. doi: 10.1007/s12272-019-01143-y 30895524

[369] Li Y Sun R. Tumor Immunotherapy: New Aspects of Natural Killer Cells. Chin J Cancer Res (2018) 30:173–96. doi: 10.21147/j.issn.1000-9604.2018.02.02 PMC5953955 29861604

[370] Zhang C Hu Y Shi C. Targeting Natural Killer Cells for Tumor Immunotherapy. Front Immunol (2020) 11:60. doi: 10.3389/fimmu.2020.00060 32140153 PMC7042203

[371] Marcus A Gowen BG Thompson TW Iannello A Ardolino M Deng W. Recognition of Tumors by the Innate Immune System and Natural Killer Cells. Adv Immunol (2014) 122:91–128. doi: 10.1016/B978-0-12-800267-4.00003-1 24507156 PMC4228931

[372] Li Q Liu X Wang D Wang Y Lu H Wen S. Prognostic Value of Tertiary Lymphoid Structure and Tumour Infiltrating Lymphocytes in Oral Squamous Cell Carcinoma. Int J Oral Sci (2020) 12:24. doi: 10.1038/s41368-020-00092-3 32934197 PMC7493903

[373] Chambers AM Lupo KB Matosevic S. Tumor Microenvironment-Induced Immunometabolic Reprogramming of Natural Killer Cells. Front Immunol (2018) 9:2517–. doi: 10.3389/fimmu.2018.02517 PMC6235907 30467503

[374] Hodgins JJ Khan ST Park MM Auer RC Ardolino M. Killers 2.0: NK Cell Therapies at the Forefront of Cancer Control. J Clin Invest (2019) 129:3499–510. doi: 10.1172/JCI129338 PMC6715409 31478911

[375] Spörri R Joller N Hilbi H Oxenius A. A Novel Role for Neutrophils as Critical Activators of NK Cells. J Immunol (2008) 181:7121–30. doi: 10.4049/jimmunol.181.10.7121 18981133

[376] Jaeger BN Donadieu J Cognet C Bernat C Ordoñez-Rueda D Barlogis V. Neutrophil Depletion Impairs Natural Killer Cell Maturation, Function, and Homeostasis. J Exp Med (2012) 209:565–80. doi: 10.1084/jem.20111908 PMC3302230 22393124

[377] Paul S Lal G. The Molecular Mechanism of Natural Killer Cells Function and Its Importance in Cancer Immunotherapy. Front Immunol (2017) 8:1124. doi: 10.3389/fimmu.2017.01124 28955340 PMC5601256

[378] Ma T Renz BW Ilmer M Koch D Yang Y Werner J. Myeloid-Derived Suppressor Cells in Solid Tumors. Cells (2022) 11:310–32. doi: 10.3390/cells11020310 PMC8774531 35053426

[379] Liu C Yu S Kappes J Wang J Grizzle WE Zinn KR. Expansion of Spleen Myeloid Suppressor Cells Represses NK Cell Cytotoxicity in Tumor-Bearing Host. Blood (2007) 109:4336–42. doi: 10.1182/blood-2006-09-046201 PMC1885503 17244679

[380] Stiff A Trikha P Mundy-Bosse B McMichael E Mace TA Benner B. Nitric Oxide Production by Myeloid-Derived Suppressor Cells Plays a Role in Impairing Fc Receptor-Mediated Natural Killer Cell Function. Clin Cancer Res (2018) 24:1891–904. doi: 10.1158/1078-0432.CCR-17-0691 PMC7184799 29363526

[381] Greene S Robbins Y Mydlarz WK Huynh AP Schmitt NC Friedman J. Inhibition of MDSC Trafficking With SX-682, a CXCR1/2 Inhibitor, Enhances NK-Cell Immunotherapy in Head and Neck Cancer Models. Clin Cancer Res (2020) 26:1420–31. doi: 10.1158/1078-0432.CCR-19-2625 PMC7073293 31848188

[382] Costantini C Cassatella MA. The Defensive Alliance Between Neutrophils and NK Cells as a Novel Arm of Innate Immunity. J Leukoc Biol (2011) 89:221–33. doi: 10.1189/jlb.0510250 20682626

[383] Ueda R Narumi K Hashimoto H Miyakawa R Okusaka T Aoki K. Interaction of Natural Killer Cells With Neutrophils Exerts a Significant Antitumor Immunity in Hematopoietic Stem Cell Transplantation Recipients. Cancer Med (2016) 5:49–60. doi: 10.1002/cam4.550 26589884 PMC4708905

[384] Chen F Yao C Feng Y Yu Y Guo H Yan J. The Identification of Neutrophils-Mediated Mechanisms and Potential Therapeutic Targets for the Management of Sepsis-Induced Acute Immunosuppression Using Bioinformatics. Med (Baltimore) (2021) 100:e24669. doi: 10.1097/MD.0000000000024669 PMC9282053 33761636

[385] Marçais A Viel S Grau M Henry T Marvel J Walzer T. Regulation of Mouse NK Cell Development and Function by Cytokines. Front Immunol (2013) 4:450. doi: 10.3389/fimmu.2013.00450 24376448 PMC3859915

[386] Prlic M Blazar BR Farrar MA Jameson SC. *In Vivo* Survival and Homeostatic Proliferation of Natural Killer Cells. J Exp Med (2003) 197:967–76. doi: 10.1084/jem.20021847 PMC2193876 12695488

[387] Zanoni I Spreafico R Bodio C Di Gioia M Cigni C Broggi A. IL-15 Cis Presentation is Required for Optimal NK Cell Activation in Lipopolysaccharide-Mediated Inflammatory Conditions. Cell Rep (2013) 4:1235–49. doi: 10.1016/j.celrep.2013.08.021 24055061

[388] Reading PC Whitney PG Barr DP Wojtasiak M Mintern JD Waithman J. IL-18, But Not IL-12, Regulates NK Cell Activity Following Intranasal Herpes Simplex Virus Type 1 Infection. J Immunol (2007) 179:3214–21. doi: 10.4049/jimmunol.179.5.3214 17709537

[389] Leung BP Culshaw S Gracie JA Hunter D Canetti CA Campbell C. A Role for IL-18 in Neutrophil Activation. J Immunol (2001) 167:2879–86. doi: 10.4049/jimmunol.167.5.2879 11509635

[390] Gebhardt C Riehl A Durchdewald M Németh J Fürstenberger G Müller-Decker K. RAGE Signaling Sustains Inflammation and Promotes Tumor Development. J Exp Med (2008) 205:275–85. doi: 10.1084/jem.20070679 PMC2271015 18208974

[391] Lindemann RA Lala A Miyasaki KT. The *In Vitro* Effect of Human Polymorphonuclear Leukocyte Azurophil Granule Components on Natural Killer Cell Cytotoxicity. Oral Microbiol Immunol (1994) 9:186–92. doi: 10.1111/j.1399-302X.1994.tb00057.x 7936726

[392] Yamazaki T Aoki Y. Cathepsin G Enhances Human Natural Killer Cytotoxicity. Immunology (1998) 93:115–21. doi: 10.1046/j.1365-2567.1998.00397.x PMC1364114 9536127

[393] Shau H Kim A Golub SH. Modulation of Natural Killer and Lymphokine-Activated Killer Cell Cytotoxicity by Lactoferrin. J Leukoc Biol (1992) 51:343–9. doi: 10.1002/jlb.51.4.343 1564398

[394] Rezvani K Rouce RH. The Application of Natural Killer Cell Immunotherapy for the Treatment of Cancer. Front Immunol (2015) 6:578. doi: 10.3389/fimmu.2015.00578 26635792 PMC4648067

[395] Hu W Wang G Huang D Sui M Xu Y. Cancer Immunotherapy Based on Natural Killer Cells: Current Progress and New Opportunities. Front Immunol (2019) 10:1205. doi: 10.3389/fimmu.2019.01205 31214177 PMC6554437

[396] Kubota A Kubota S Lohwasser S Mager DL Takei F. Diversity of NK Cell Receptor Repertoire in Adult and Neonatal Mice. J Immunol (1999) 163:212–6. 10384118

[397] Raulet DH Vance RE. Self-Tolerance of Natural Killer Cells. Nat Rev Immunol (2006) 6:520–31. doi: 10.1038/nri1863 16799471

[398] Liu RB Engels B Arina A Schreiber K Hyjek E Schietinger A. Densely Granulated Murine NK Cells Eradicate Large Solid Tumors. Cancer Res (2012) 72:1964–74. doi: 10.1158/0008-5472.CAN-11-3208 PMC3680344 22374983

[399] Agarwal R Chaudhary M Bohra S Bajaj S. Evaluation of Natural Killer Cell (CD57) as a Prognostic Marker in Oral Squamous Cell Carcinoma: An Immunohistochemistry Study. J Oral Maxillofac Pathol (2016) 20:173–7. doi: 10.4103/0973-029X.185933 PMC4989542 27601804

[400] Godfrey DI MacDonald HR Kronenberg M Smyth MJ Kaer LV. NKT Cells: What’s in a Name? Nat Rev Immunol (2004) 4:231–7. doi: 10.1038/nri1309 15039760

[401] Toura I Kawano T Akutsu Y Nakayama T Ochiai T Taniguchi M. Cutting Edge: Inhibition of Experimental Tumor Metastasis by Dendritic Cells Pulsed With Alpha-Galactosylceramide. J Immunol (1999) 163:2387–91. 10452972

[402] Kawano T Cui J Koezuka Y Toura I Kaneko Y Sato H. Natural Killer-Like Nonspecific Tumor Cell Lysis Mediated by Specific Ligand-Activated Valpha14 NKT Cells. Proc Natl Acad Sci U S A (1998) 95:5690–3. doi: 10.1073/pnas.95.10.5690 PMC20440 9576945

[403] Wang H Feng D Park O Yin S Gao B. Invariant NKT Cell Activation Induces Neutrophil Accumulation and Hepatitis: Opposite Regulation by IL-4 and IFN-γ. Hepatology (2013) 58:1474–85. doi: 10.1002/hep.26471 PMC3758807 23686838

[404] Li L Huang L Sung SS Lobo PI Brown MG Gregg RK. NKT Cell Activation Mediates Neutrophil IFN-Gamma Production and Renal Ischemia-Reperfusion Injury. J Immunol (2007) 178:5899–911. doi: 10.4049/jimmunol.178.9.5899 17442974

[405] Hwang SJ Kim S Park WS Chung DH. IL-4-Secreting NKT Cells Prevent Hypersensitivity Pneumonitis by Suppressing IFN-Gamma-Producing Neutrophils. J Immunol (2006) 177:5258–68. doi: 10.4049/jimmunol.177.8.5258 17015711

[406] Huang E Liu R Lu Z Liu J Liu X Zhang D. NKT Cells Mediate the Recruitment of Neutrophils by Stimulating Epithelial Chemokine Secretion During Colitis. Biochem Biophys Res Commun (2016) 474:252–8. doi: 10.1016/j.bbrc.2016.04.024 27063801

[407] Meira LB Bugni JM Green SL Lee CW Pang B Borenshtein D. DNA Damage Induced by Chronic Inflammation Contributes to Colon Carcinogenesis in Mice. J Clin Invest (2008) 118:2516–25. doi: 10.1172/JCI35073 PMC2423313 18521188

[408] Song L Asgharzadeh S Salo J Engell K Wu HW Sposto R. Valpha24-Invariant NKT Cells Mediate Antitumor Activity *via* Killing of Tumor-Associated Macrophages. J Clin Invest (2009) 119:1524–36. doi: 10.1172/JCI37869 PMC2689106 19411762

[409] Molling JW Langius JA Langendijk JA Leemans CR Bontkes HJ van der Vliet HJ. Low Levels of Circulating Invariant Natural Killer T Cells Predict Poor Clinical Outcome in Patients With Head and Neck Squamous Cell Carcinoma. J Clin Oncol (2007) 25:862–8. doi: 10.1200/JCO.2006.08.5787 17327607

[410] Singh AK Shukla NK Das SN. Altered Invariant Natural Killer T Cell Subsets and its Functions in Patients With Oral Squamous Cell Carcinoma. Scand J Immunol (2013) 78:468–77. doi: 10.1111/sji.12104 23980793

[411] Stasikowska-Kanicka O Wągrowska-Danilewicz M Danilewicz M. CD8+ and CD163+ Infiltrating Cells and PD-L1 Immunoexpression in Oral Leukoplakia and Oral Carcinoma. Apmis (2018) 126:732–8. doi: 10.1111/apm.12881 30160018

[412] Mori K Haraguchi S Hiori M Shimada J Ohmori Y. Tumor-Associated Macrophages in Oral Premalignant Lesions Coexpress CD163 and STAT1 in a Th1-Dominated Microenvironment. BMC Cancer (2015) 15:573. doi: 10.1186/s12885-015-1587-0 26242181 PMC4525742

[413] Bondad-Palmario GG. Histological and Immunochemical Studies of Oral Leukoplakia: Phenotype and Distribution of Immunocompetent Cells. J Philipp Dent Assoc (1995) 47:3–18. 9227108

[414] De Costa AM Schuyler CA Walker DD Young MR. Characterization of the Evolution of Immune Phenotype During the Development and Progression of Squamous Cell Carcinoma of the Head and Neck. Cancer Immunol Immunother (2012) 61:927–39. doi: 10.1007/s00262-011-1154-8 PMC5925419 22116344

[415] Woodford D Johnson SD De Costa AM Young MR. An Inflammatory Cytokine Milieu is Prominent in Premalignant Oral Lesions, But Subsides When Lesions Progress to Squamous Cell Carcinoma. J Clin Cell Immunol (2014) 5:230–37. doi: 10.4172/2155-9899.1000230 PMC4240319 25419481

[416] Johnson SD De Costa AM Young MR. Effect of the Premalignant and Tumor Microenvironment on Immune Cell Cytokine Production in Head and Neck Cancer. Cancers (Basel) (2014) 6:756–70. doi: 10.3390/cancers6020756 PMC4074802 24698959

[417] Chaudhary M Gadbail AR Vidhale G Mankar Gadbail MP Gondivkar SM Gawande M. Comparison of Myofibroblasts Expression in Oral Squamous Cell Carcinoma, Verrucous Carcinoma, High Risk Epithelial Dysplasia, Low Risk Epithelial Dysplasia and Normal Oral Mucosa. Head Neck Pathol (2012) 6:305–13. doi: 10.1007/s12105-012-0335-x PMC3422591 22392407

[418] Trellakis S Bruderek K Dumitru CA Gholaman H Gu X Bankfalvi A. Polymorphonuclear Granulocytes in Human Head and Neck Cancer: Enhanced Inflammatory Activity, Modulation by Cancer Cells and Expansion in Advanced Disease. Int J Cancer (2011) 129:2183–93. doi: 10.1002/ijc.25892 21190185

[419] Mohtasham N Babakoohi S Shiva A Shadman A Kamyab-Hesari K Shakeri MT. Immunohistochemical Study of P53, Ki-67, MMP-2 and MMP-9 Expression at Invasive Front of Squamous Cell and Verrucous Carcinoma in Oral Cavity. Pathol Res Pract (2013) 209:110–4. doi: 10.1016/j.prp.2012.11.002 23273944

[420] Vasquez-Dunddel D Pan F Zeng Q Gorbounov M Albesiano E Fu J. STAT3 Regulates Arginase-I in Myeloid-Derived Suppressor Cells From Cancer Patients. J Clin Invest (2013) 123:1580–9. doi: 10.1172/JCI60083 PMC3613901 23454751

[421] Zeng Q Fu J Korrer M Gorbounov M Murray PJ Pardoll D. Caspase-1 From Human Myeloid-Derived Suppressor Cells Can Promote T Cell-Independent Tumor Proliferation. Cancer Immunol Res (2018) 6:566–77. doi: 10.1158/2326-6066.CIR-17-0543 PMC9336537 29653983

[422] Weed DT Vella JL Reis IM de la Fuente AC Gomez C Sargi Z. Tadalafil Reduces Myeloid-Derived Suppressor Cells and Regulatory T Cells and Promotes Tumor Immunity in Patients With Head and Neck Squamous Cell Carcinoma. Clin Cancer Res (2015) 21:39–48. doi: 10.1158/1078-0432.CCR-14-1711 25320361 PMC4322895

[423] Mao L Fan TF Wu L Yu GT Deng WW Chen L. Selective Blockade of B7-H3 Enhances Antitumour Immune Activity by Reducing Immature Myeloid Cells in Head and Neck Squamous Cell Carcinoma. J Cell Mol Med (2017) 21:2199–210. doi: 10.1111/jcmm.13143 PMC5571514 28401653

[424] Younis RH Han KL Webb TJ. Human Head and Neck Squamous Cell Carcinoma-Associated Semaphorin 4d Induces Expansion of Myeloid-Derived Suppressor Cells. J Immunol (2016) 196:1419–29. doi: 10.4049/jimmunol.1501293 PMC4722498 26740106

[425] Tamagnone L Comoglio PM. To Move or Not to Move? Semaphorin Signalling in Cell Migration. EMBO Rep (2004) 5:356–61. doi: 10.1038/sj.embor.7400114 PMC1299025 15060572

[426] Chabbert-de Ponnat I Marie-Cardine A Pasterkamp RJ Schiavon V Tamagnone L Thomasset N. Soluble CD100 Functions on Human Monocytes and Immature Dendritic Cells Require Plexin C1 and Plexin B1, Respectively. Int Immunol (2005) 17:439–47. doi: 10.1093/intimm/dxh224 15746246

[427] Zhou H Yang YH Binmadi NO Proia P Basile JR. The Hypoxia-Inducible Factor-Responsive Proteins Semaphorin 4D and Vascular Endothelial Growth Factor Promote Tumor Growth and Angiogenesis in Oral Squamous Cell Carcinoma. Exp Cell Res (2012) 318:1685–98. doi: 10.1016/j.yexcr.2012.04.019 PMC3389129 22652457

[428] Ni YH Ding L Huang XF Dong YC Hu QG Hou YY. Microlocalization of CD68+ Tumor-Associated Macrophages in Tumor Stroma Correlated With Poor Clinical Outcomes in Oral Squamous Cell Carcinoma Patients. Tumour Biol (2015) 36:5291–8. doi: 10.1007/s13277-015-3189-5 25666753

[429] Jiang C Yuan F Wang J Wu L. Oral Squamous Cell Carcinoma Suppressed Antitumor Immunity Through Induction of PD-L1 Expression on Tumor-Associated Macrophages. Immunobiology (2017) 222:651–7. doi: 10.1016/j.imbio.2016.12.002 28017495

[430] Kubota K Moriyama M Furukawa S Rafiul H Maruse Y Jinno T. CD163(+)CD204(+) Tumor-Associated Macrophages Contribute to T Cell Regulation *via* Interleukin-10 and PD-L1 Production in Oral Squamous Cell Carcinoma. Sci Rep (2017) 7:1755. doi: 10.1038/s41598-017-01661-z 28496107 PMC5431876

[431] Costa NL Valadares MC Souza PP Mendonça EF Oliveira JC Silva TA. Tumor-Associated Macrophages and the Profile of Inflammatory Cytokines in Oral Squamous Cell Carcinoma. Oral Oncol (2013) 49:216–23. doi: 10.1016/j.oraloncology.2012.09.012 23089461

[432] Jablonska E Garley M Surazynski A Grubczak K Iwaniuk A Borys J. Neutrophil Extracellular Traps (NETs) Formation Induced by TGF-β in Oral Lichen Planus - Possible Implications for the Development of Oral Cancer. Immunobiology (2020) 225:151901. doi: 10.1016/j.imbio.2019.151901 31882256

[433] Kasten KR Muenzer JT Caldwell CC. Neutrophils are Significant Producers of IL-10 During Sepsis. Biochem Biophys Res Commun (2010) 393:28–31. doi: 10.1016/j.bbrc.2010.01.066 20097159 PMC2830356

[434] Zhao S Wu D Wu P Wang Z Huang J. Serum IL-10 Predicts Worse Outcome in Cancer Patients: A Meta-Analysis. PLoS One (2015) 10:e0139598. doi: 10.1371/journal.pone.0139598 26440936 PMC4595202

[435] Yao JG Gao LB Liu YG Li J Pang GF. Genetic Variation in Interleukin-10 Gene and Risk of Oral Cancer. Clin Chim Acta (2008) 388:84–8. doi: 10.1016/j.cca.2007.10.012 17980158

[436] Vairaktaris E Yapijakis C Serefoglou Z Derka S Vassiliou S Nkenke E. The Interleukin-10 (-1082A/G) Polymorphism is Strongly Associated With Increased Risk for Oral Squamous Cell Carcinoma. Anticancer Res (2008) 28:309–14. 18383862

[437] Huang W Song J Jia XW Chen YX Shi J Jiang X. Interleukin-10 Rs1800896 Polymorphism is Associated With Increased Head and Neck Cancer Risk But Not Associated With its Clinical Stages. Oncotarget (2017) 8:37217–24. doi: 10.18632/oncotarget.16660 PMC5514904 28410223

[438] Wagner S Wittekindt C Reuschenbach M Hennig B Thevarajah M Würdemann N. CD56-Positive Lymphocyte Infiltration in Relation to Human Papillomavirus Association and Prognostic Significance in Oropharyngeal Squamous Cell Carcinoma. Int J Cancer (2016) 138:2263–73. doi: 10.1002/ijc.29962 26662627

[439] Stabile H Fionda C Gismondi A Santoni A. Role of Distinct Natural Killer Cell Subsets in Anticancer Response. Front Immunol (2017) 8:293. doi: 10.3389/fimmu.2017.00293 28360915 PMC5352654

[440] Moy JD Moskovitz JM Ferris RL. Biological Mechanisms of Immune Escape and Implications for Immunotherapy in Head and Neck Squamous Cell Carcinoma. Eur J Cancer (2017) 76:152–66. doi: 10.1016/j.ejca.2016.12.035 PMC5459368 28324750

[441] Zingoni A Ardolino M Santoni A Cerboni C. NKG2D and DNAM-1 Activating Receptors and Their Ligands in NK-T Cell Interactions: Role in the NK Cell-Mediated Negative Regulation of T Cell Responses. Front Immunol (2012) 3:408. doi: 10.3389/fimmu.2012.00408 23316196 PMC3540764

[442] Bisheshar SK De Ruiter EJ Devriese LA Willems SM. The Prognostic Role of NK Cells and Their Ligands in Squamous Cell Carcinoma of the Head and Neck: A Systematic Review and Meta-Analysis. Oncoimmunology (2020) 9(1):e1747345. doi: 10.1080/2162402X.2020.1747345 PMC7185215 32363116

[443] Hall LJ Murphy CT Quinlan A Hurley G Shanahan F Nally K. Natural Killer Cells Protect Mice From DSS-Induced Colitis by Regulating Neutrophil Function *via* the NKG2A Receptor. Mucosal Immunol (2013) 6:1016–26. doi: 10.1038/mi.2012.140 23340823

[444] Zou Z Zuo D Yang J Fan H. The ANXA1 Released From Intestinal Epithelial Cells Alleviate DSS-Induced Colitis by Improving NKG2A Expression of Natural Killer Cells. Biochem Biophys Res Commun (2016) 478:213–20. doi: 10.1016/j.bbrc.2016.07.066 27435504

